# Hyperspectral Fluorescence Imaging with a New Polarity‐Ultrasensitive Fluorescent Probe

**DOI:** 10.1002/advs.202508792

**Published:** 2025-06-23

**Authors:** Ri Zhou, Hao Sha, Shengjie Fu, Guannan Liu, Jinbei Wei, Chenguang Wang, Shangguo Hou, Geyu Lu

**Affiliations:** ^1^ State Key Laboratory of Integrated Optoelectronics (JLU Region) Key Laboratory of Advanced Gas Sensors of Jilin Province College of Electronic Science and Engineering Jilin University Changchun 130012 China; ^2^ School of Computer Science and Technology Harbin Institute of Technology (Shenzhen) Shenzhen 518006 China; ^3^ Institute of Systems and Physical Biology Shenzhen Bay Laboratory Shenzhen 518132 China

**Keywords:** fluorescent probe, hyperspectral fluorescence imaging, polarity mapping, polarity‐ultrasensitive, wavelength/polarity encoding

## Abstract

Hyperspectral fluorescence imaging (HSFI) could simultaneously offer morphological visualization and microenvironmental information through fluorescence wavelength‐shifting of microenvironmental‐sensitive fluorescent probes, yet its advancement is directly hindered by the scarcity of probes with sufficient spectral sensitivity. Herein, this study focuses on the development of an innovative fluorescent probe that exhibits heightened spectral sensitivity to microenvironmental changes and its advanced HSFI application. First, the comprehensive investigation of various donor‐π‐acceptor molecules uncovers the critical role of π‐spacer on the wavelength sensitivity to microenvironment polarity. Based on this insight, a new fluorescent probe **Lipi‐PS** of whose wavelength sensitivity represents the highest level so far is rationally developed. Further combining with the features of lipid droplets (LDs) targeting and high photostability of **Lipi‐PS**, as well as the newly established wavelength and polarity encoding method, HSFI is successfully realized in cells, tissues, and zebrafishes. Accordingly, the structural morphologies, fluorescence wavelengths, and polarities are intuitively visualized in the HSFI images. Notably, a special HSFI of single LD is achieved using **Lipi‐PS** in a custom‐built 3D‐SpecDIM system, allowing 5D tracking (*xyzλt*) of individual LDs for the first time. This enables unprecedented correlation of polarity‐dynamic behavior at the single‐LD level.

## Graphical abstract

Based on the critical role of the π‐spacer in determining wavelength sensitivity to microenvironmental polarity, a new fluorescent probe, **Lipi‐PS** is rationally developed, which exhibits the highest polarity‐sensitivity so far. Its integration with hyperspectral fluorescence imaging enables multiscale polarity mapping across cells, tissues, and zebrafish, as well as the first dynamic tracking of individual LDs in 5D (*xyzλt*).

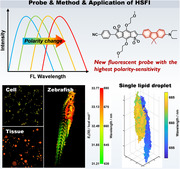

## 1 Introduction

**Figure 1:**
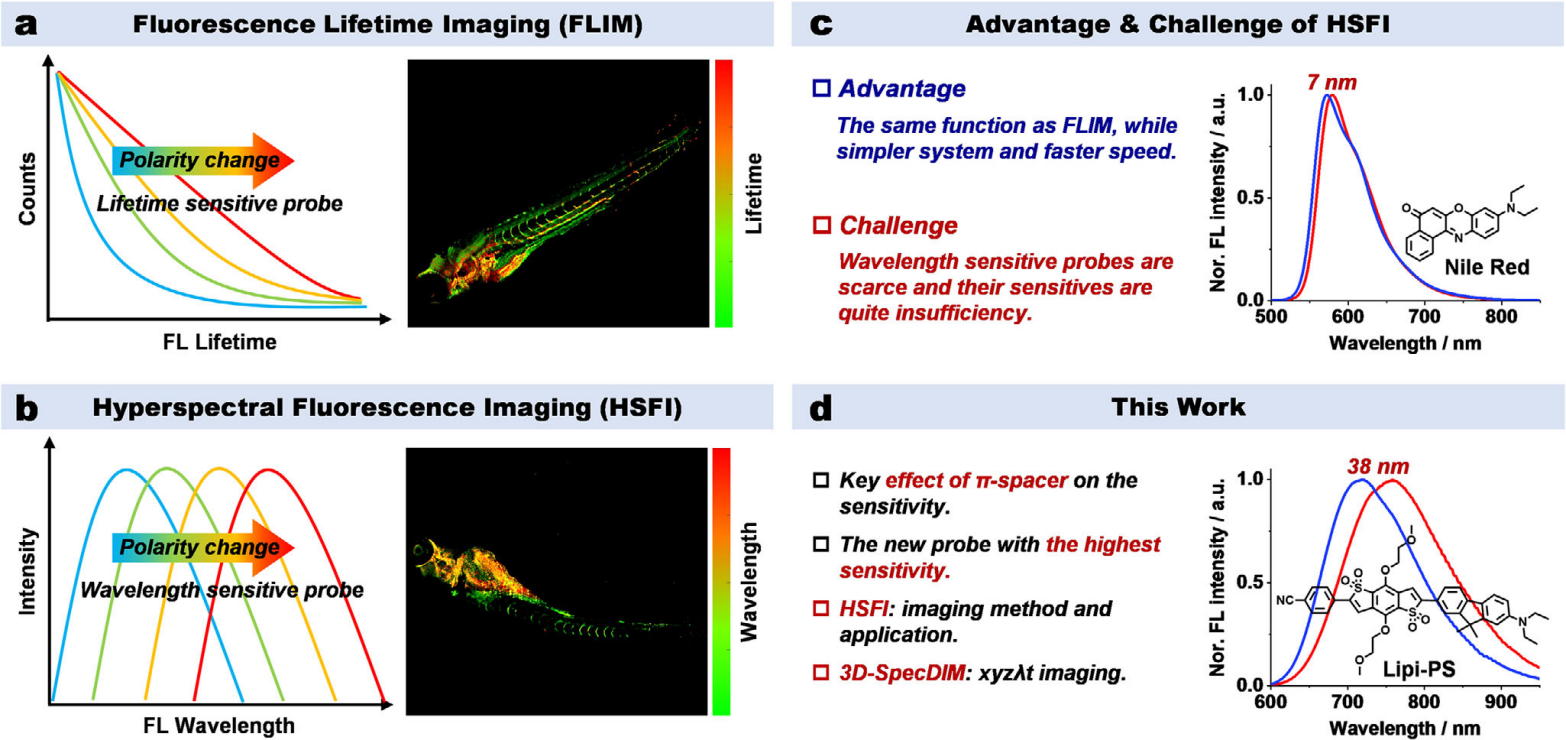
Overview of FLIM, HSFI, and this work. a) Fluorescence lifetime‐sensitive probe and FLIM. b) Fluorescence wavelength‐sensitive probe and HSFI. c) The advantages and challenges of HSFI. d) The development of a new fluorescent probe **Lipi‐PS** with wavelength‐ultrasensitive character and its HSFI application in this work.

Due to the advantages of non‐invasive nature, high sensitivity and spatiotemporal resolution, the fluorescence imaging has become a powerful tool in biomedical research.^[^ ^1^ ^]^ The traditional fluorescence imaging techniques, such as wide‐field, confocal and two‐photon microscopy, enable observation of the morphological structures of tissues, cells and subcellular components in different gray brightness for each pixel. While in recent years, the fluorescence lifetime imaging microscopy (FLIM) has built upon the traditional fluorescence imaging techniques to further provide the fluorescence lifetime for each pixel.^[^ ^2^, ^3^, ^4^ ^]^ Combining with the fluorescent probes whose lifetimes are sensitive to the microenvironment (e.g., polarity, viscosity or pressure), the FLIM can not only visualize the morphological structures but also reflect the microenvironmental information of biological samples through changes in fluorescence lifetime (**Figure** **1**a).^[^ ^5^, ^6^, ^7^, ^8^, ^9^ ^]^ Undoubtedly, the FLIM adding a new dimension (i.e., fluorescence lifetime) to the traditional fluorescence imaging (i.e., fluorescence brightness) is of great importance to this filed.

Similar to the FLIM, the hyperspectral fluorescence imaging (HSFI) extends another new dimension of the fluorescence wavelength for each pixel to the traditional fluorescence imaging.^[^ ^10^, ^11^, ^12^, ^13^, ^14^, ^15^, ^16^ ^]^ Employing with the fluorescent probes whose wavelengths are sensitive to the microenvironment, HSFI can also observe the morphological structures and provide the information on microenvironmental changes (Figure 1b). Furthermore, in comparison to FLIM, HSFI offers the great potential advantages of simpler imaging equipment and faster imaging speed. However, the development of HSFI lags dramatically behind that of FLIM, and the biological application of HSFI is very limited.^[^ ^17^, ^18^, ^19^, ^20^ ^]^ This is because of that the lifetime‐sensitive fluorescent probes have been widely developed, while the wavelength‐sensitive ones are relatively scarce and particularly the wavelength sensitivity is extremely not sufficient.^[^ ^21^, ^22^, ^23^, ^24^, ^25^, ^26^, ^27^, ^28^, ^29^, ^30^, ^31^, ^32^, ^33^ ^]^ For example, the shift of fluorescence wavelength of Nile Red (the representative wavelength‐sensitive probe toward the microenvironmental polarity) is only 7 nm upon changing the solvent polarity from toluene to dioxane (Figure 1c). Considering the microenvironmental polarity change in biological systems is usually even smaller, the shift of fluorescence wavelength less than several nanometers would be extremely challenging to be detected by HSFI. Therefore, the development of new fluorescent probes featuring with the ultrahigh wavelength sensitivity toward the microenvironment is highly crucial for HSFI application and advancement.

In this context, we herein have focused on developing a new ultrasensitive fluorescent probe and demonstrating its HSFI application. We have initially investigated three series of donor‐π‐acceptor (D‐π‐A) type molecules and discovered that the π‐spacer plays a key role in the wavelength sensitivity toward the microenvironment polarity. After that, we have rationally developed a new fluorescent probe **Lipi‐PS** (means lipid droplets polarity sensor) of whose wavelength sensitivity is up to six times of Nile Red and represents the highest sensitivity so far (Figure 1d). Further combining with the features of strong red to near‐infrared (NIR) emission, desired cellular lipid droplets (LDs) staining selectivity as well as high photostability, **Lipi‐PS** has been successfully applied in HSFI of cells, tissues, and zebrafishes. As a result, the LDs polarities and morphologies have been intuitively visualized in the HSFI images, and their changing processes have been in situ monitored via time‐lapse HSFI in living cells. Moreover, a special HSFI of even single LD has been impressively realized by using **Lipi‐PS** in the 3D single molecule fluorescence spectral dynamics imaging microscopy (3D‐SpecDIM).^[^ ^34^ ^]^ Consequently, the LD has been tracked up to 5 dimensions (*xyzλt*) for the first time, enabling to study of single LD at the unprecedented level.

## 2 Results

### 2.1 The effects of Different Moieties on the Polarity Sensitivities of D‐π‐A Type Molecules

To develop wavelength‐ultrasensitive fluorescent probes toward microenvironment polarity for HSFI, we have synthesized 12 distinct D‐π‐A type molecules and systematically modulated the donor, π‐spacer, and acceptor groups to investigate their respective impacts on the polarity sensitivity of molecules (Schemes S1, S2, Supporting Information). The detailed synthesis and structural characterization are described in the Supporting Information.

**Figure 2:**
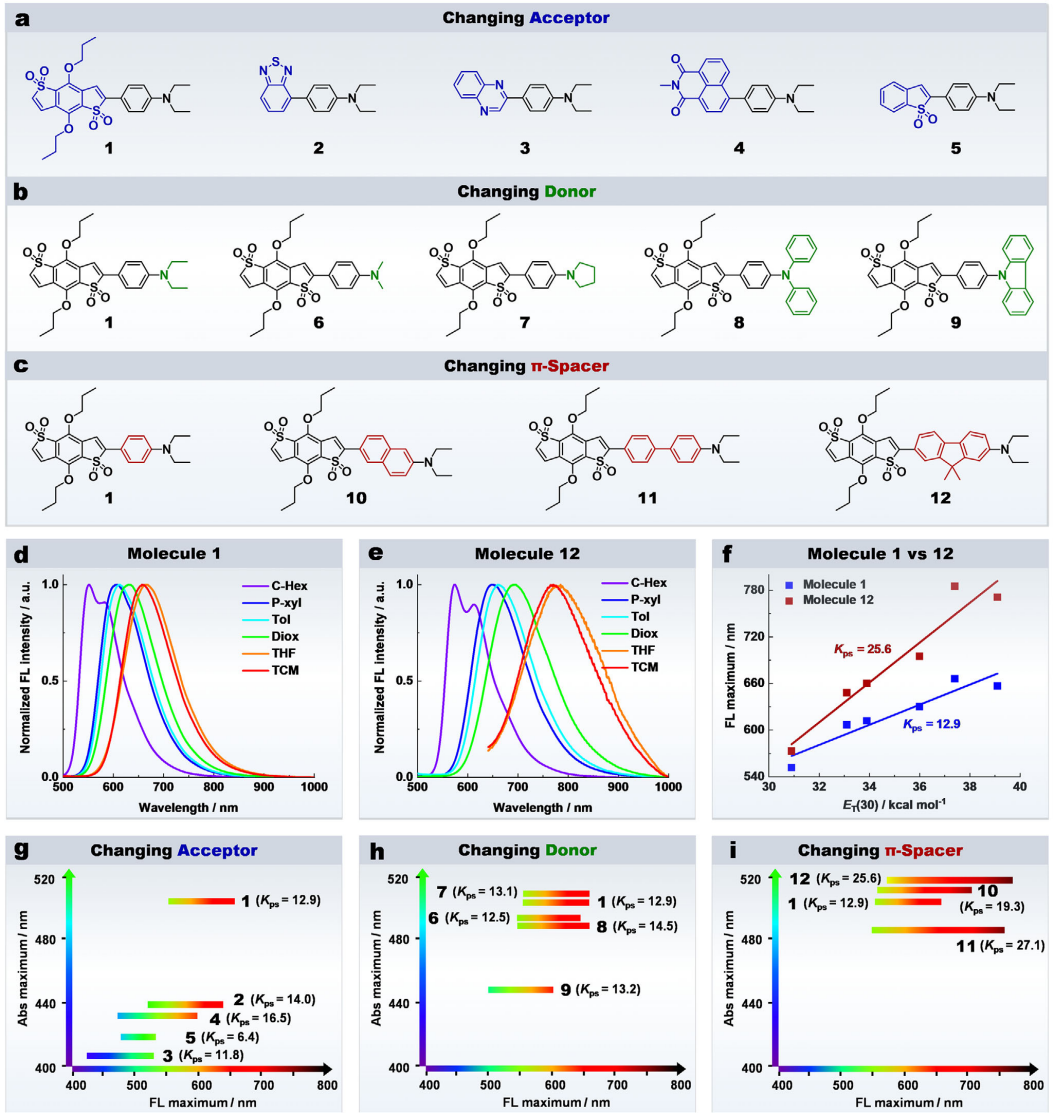
The effects of different moieties on the polarity sensitivities of D‐π‐A type molecules. a–c) The D‐π‐A type molecules employing with various acceptor, donor, and π‐spacer groups. d,e) Normalized fluorescence spectra of molecules **1** and **12** in various organic solvents. f) Linear fitting between the polarity index *E*_T_(30) values of various solvents and the fluorescence maxima of molecules **1** and **12**. g–i) The fluorescence maxima shift ranges from cyclohexane to chloroform, and the absorption maxima in toluene of molecules **1** to **12**.

First, we have explored the impact of acceptor groups. As shown in **Figure** **2**a, molecules **1** to **5** share the same donor (NEt_2_) and π‐spacer (phenyl) but feature different acceptor groups. Comparison of their emission spectra reveals that the acceptor group significantly influences the polarity sensitivity of molecules (Figure S1 and Table S1, Supporting Information). For example, when the solvent polarity increases from cyclohexane to chloroform, the bathochromic shift of the emission of molecule **1** (with a benzodithiophene tetra‐oxide acceptor) is 105 nm (from 552 to 657 nm) (Figure 2d). In contrast, molecule **5** (with a benzothiophene dioxide acceptor) exhibits a much smaller shift of only 55 nm (from 480 to 535 nm, Figure S1e, Supporting Information). To further quantify the sensitivity of these molecules to the environmental polarity, the fluorescence wavelength maximum has been plotted as a function of the solvent polarity index *E*_T_(30) and the polarity slope (*K*_ps_) has been thus determined (Figure S2, Supporting Information). Overall, molecules **1** to **4** exhibit relatively high *K*_ps_ values (11.8–16.5), while molecule **5** has a much lower *K*_ps_ of only 6.4 (Figure 2g). Given that the absorption peak of molecule **1** (*λ*_abs_ = 506 nm) is significantly longer than those of the other molecules (≤ 440 nm), it is advantageous for excitation with longer‐wavelength lasers, thereby reducing cellular photodamage. Therefore, the benzodithiophene tetra‐oxide acceptor utilised in molecule **1** is an excellent choice for developing polarity‐sensitive fluorescent probe.

Second, we have investigated the effect of different donor groups while keeping the acceptor as benzodithiophene tetra‐oxide and the π‐spacer as phenyl (Figure 2b). As shown in Figure S3 and Table S2 (Supporting Information), various amine substituents have a modest impact on the absorption wavelength but minimal influence on the fluorescence wavelength shift range (i.e., polarity sensitivity). The *K*_ps_ values of molecules **1** and **6** to **9** range from 12.5 to 14.5 (Figure 2h; Figure S4, Supporting Information). Considering both excitation wavelength and polarity sensitivity, NEt_2_ emerges as the most suitable donor group.

Third, we have explored the impact of various π‐spacers while maintaining benzodithiophene tetra‐oxide as the acceptor and NEt_2_ as the donor (Figure 2c). As shown in Figure S5 and Table S3 (Supporting Information), extending the length of the π‐spacer significantly enhances the emission spectral response of molecules. For example, molecule **10** (with a naphthalene π‐spacer) exhibits a *K*_ps_ of 19.3 (Figure S6, Supporting Information), which is notably higher than that of molecule **1** (*K*_ps_ = 12.1). Further enhancement is observed with biphenyl and fluorene π‐spacers in molecules **11** and **12**, with *K*_ps_ values of 27.1 and 25.6, respectively (Figure 2e,i). Given that molecule **12** has a longer absorption wavelength and higher fluorescence quantum yield compared to molecule **11** (Table S3, Supporting Information), the fluorene π‐spacer is identified as the optimal choice for developing a polarity‐sensitive fluorescent probe.

Overall, our systematic investigation establishes that the acceptor, donor, and π‐spacer groups collectively modulate the polarity sensitivity of D‐π‐A type molecules. Crucially, the π‐spacer–a historically underestimated moiety–emerges as the dominant sensitivity amplifier. Transitioning the π‐spacer from the commonly used phenyl (molecule **1**) to the fluorene (molecule **12**) induces a 2‐fold enhancement in polarity sensitivity (Figure 2f). This discovery redefines the molecular design strategy for polarity‐sensitive fluorophores.

### 2.2 Why the π‐Spacer Dramatically Effects the Polarity Sensitivity

Initially, we have analyzed the distributions of HOMOs (highest occupied molecular orbitals) and LUMOs (lowest unoccupied molecular orbitals) for molecules **1**, **10**, **11** and **12** (Figure S7, Supporting Information). In overall, these molecules exhibit significant spatial separation between their HOMOs and LUMOs, with the HOMOs primarily localize on the donor and π‐spacer regions, while the LUMOs predominantly reside on the acceptor parts. To quantify the degree of orbital separation, we have calculated the overlap integrals of HOMOs‒LUMOs.^[^ ^35^ ^]^ The overlap integrals for molecules **1**, **10**, **11**, and **12** are 0.60, 0.51, 0.37, and 0.43, respectively. Smaller overlap integrals indicate more pronounced separation between HOMOs and LUMOs, which correlates with the heightened polarity sensitivity observed in these molecules.

**Figure 3:**
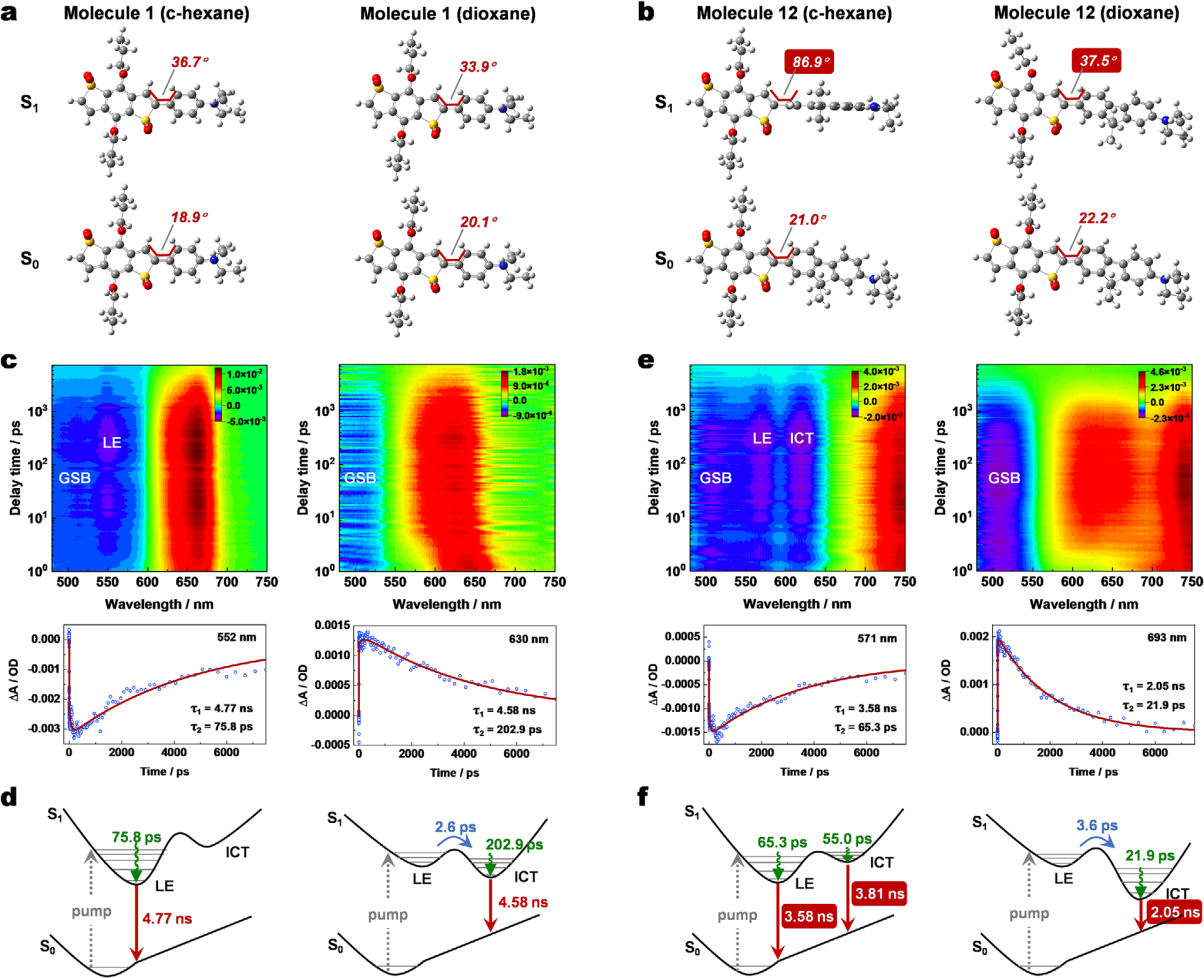
The mechanism of π‐spacer significantly affects polarity sensitivity. a,b) The optimal ground and excited state configurations of molecules **1** and **12** in cyclohexane and dioxane. c,e) Fs‐TA spectra and corresponding kinetic fitting analysis of molecules **1** and **12** in cyclohexane and dioxane. d,f) The excited‐state transform pathways of molecules **1** and **12** in cyclohexane and dioxane.

Second, we have investigated the ground and excited‐state configurations of molecules **1** and **12** in solvents of varying polarities (cyclohexane and dioxane) to elucidate the mechanism by which the π‐spacer influences polarity sensitivity. **Figure** **3**a,b illustrates that in the ground state (S_0_), the configurations of molecules **1** and **12** remain largely unchanged with increasing solvent polarity, consistent with their absorption spectra being independent of solvent polarity. In the excited state (S_1_), molecule **1** exhibits minimal configurational changes across various solvents, while molecule **12** shows significant variations. For instance, the twist angle between acceptor and π‐spacer of molecule **12** largely decreases from 86.9° in cyclohexane to 37.5° in dioxane, whereas the twist angle of molecule **1** changes only slightly (21.0° to 22.2°). The pronounced polarity sensitivity of molecule **12** is successfully elucidated by the dramatically polarity‐dependent excited‐state configuration twisting, where increasing solvent polarity decreases the twist angle and thus improves the π‐conjugation and shifts the emission to bathochromic region.

Third, femtosecond transient absorption (fs‐TA) spectroscopies have been employed to further investigate the excited‐state properties of molecules **1** and **12** in solvents of different polarities. For molecule **1** in cyclohexane, in addition to the ground state bleaching (GSB) band, a significant negative band at ≈552 nm is observed and attributed to the locally excited (LE) state (Figure 3c; Figure S8, Supporting Information). Global fitting and kinetic analysis reveal the following excited‐state pathway for molecule **1** in cyclohexane: a vibration relaxation of 75.8 ps in LE state and a decay process of 4.77 ns (Figure 3d). In dioxane, the intramolecular charge transfer (ICT) band (≈630 nm) is overlapped by the excited state absorption (ESA) band, making the corresponding negative band unobservable (Figure 3c). However, kinetic fitting analysis indicates the following pathway: a 2.6 ps transition from LE state to ICT state, a 202.9 ps vibration relaxation of ICT state, and a decay process of 4.58 ns (Figure 3d; Figure S9, Supporting Information). For molecule **12** in cyclohexane, two distinct negative bands corresponding to LE state (571 nm) and ICT state (621 nm) are captured simultaneously (Figure 3e; Figure S10, Supporting Information). Fitting analysis at these two wavelengths reveals comparable contributions from LE and ICT states. Thus, molecule **12** exhibits two pathways from S_1_ to S_0_: pathway 1 involves a 65.3 ps LE vibration relaxation and a 3.58 ns LE decay process, while pathway 2 included a 55.0 ps ICT vibration relaxation and a 3.81 ns ICT decay process (Figure 3f). In dioxane, the ICT band (≈693 nm) is again overlapped by the ESA band. However, a new signal peak emerges at 693 nm in the time‐resolved fs‐TA spectra (Figure S11, Supporting Information). Fitting analysis shows that the direct decay of LE state disappears, leaving only the ICT decay process with significantly reduced fluorescence lifetime: a 3.64 ps transition from LE state to ICT state, a 21.9 ps vibration relaxation of ICT state, and a decay process of 2.05 ns. Comparing the excited‐state dynamics of molecules **1** and **12** in cyclohexane and dioxane reveals that the S_1_ of molecule **12** is highly polarity‐dependent. Increasing solvent polarity significantly reduces its excited‐state lifetime and changes its decay pathways from two to one. In contrast, the S_1_ of molecule **1** is insensitive to solvent polarity, with its excited‐state lifetime remaining consistent.

Overall, the excited‐state configuration calculation and the fs‐TA study have demonstrated that molecule **12**′s excited‐state structure is highly sensitive to solvent polarity. Increasing solvent polarity reduces molecular twisting and improves π‐conjugation, leading to pronounced bathochromic shift of emission. In contrast, molecule **1**′s excited‐state configuration is relatively insensitive to solvent polarity.

### 2.3 The Rational Development of Target Fluorescent Probe and its Photophysical Property

**Figure 4:**
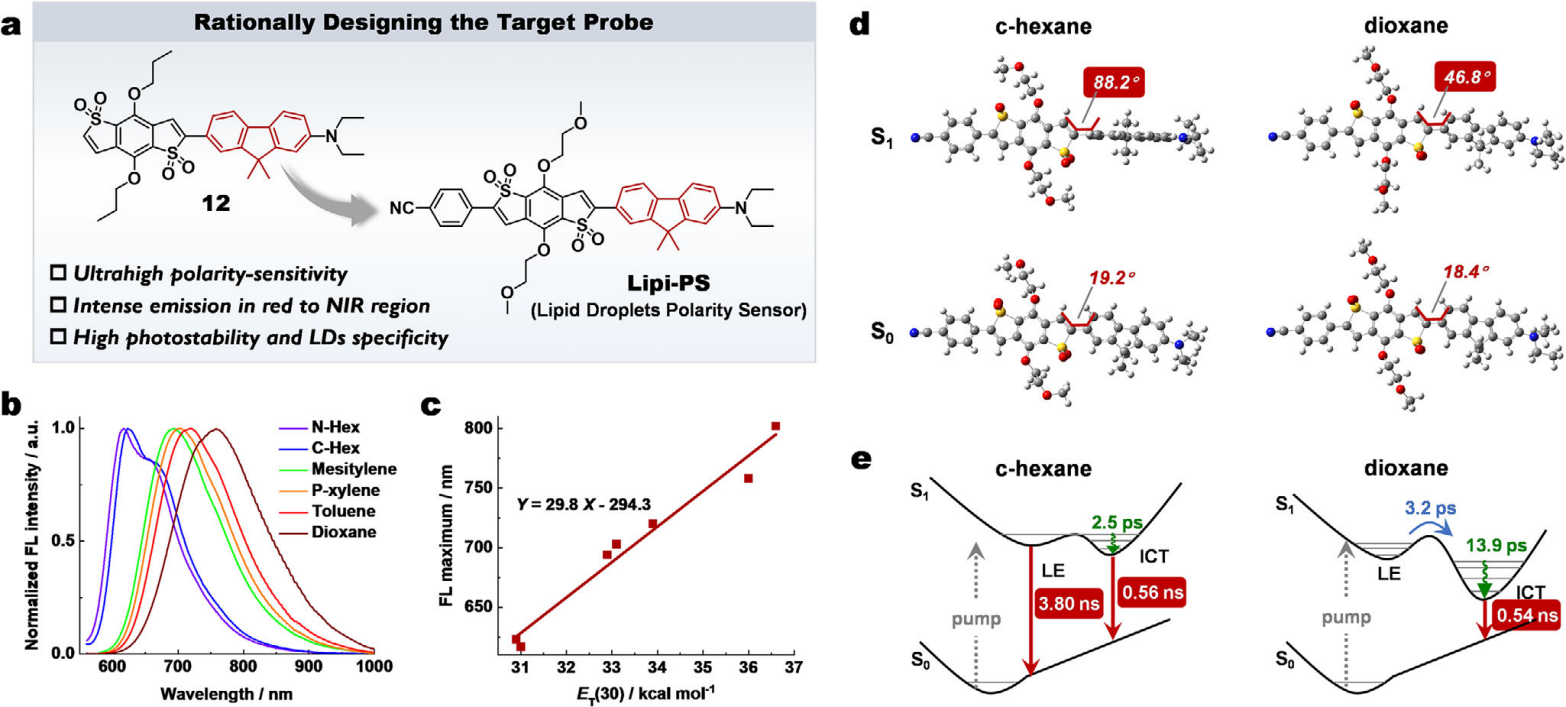
Rational design of the target fluorescent probe **Lipi‐PS** and its photophysical property. a) Molecule design strategy of **Lipi‐PS**. b) Normalized fluorescence spectra of **Lipi‐PS** in various organic solvents. c) Linear fitting between the polarity index *E*_T_(30) values of various solvents and the corresponding fluorescence maxima of **Lipi‐PS**. d) The optimal ground and excited state configurations of **Lipi‐PS** in *c*‐hexane and dioxane. e) The excited‐state transform pathways of **Lipi‐PS** in *c*‐hexane and dioxane.

Based on the before‐mentioned molecule structure‐photophysical property relationship, we have thereafter designed a next‐generation polarity sensor using molecule **12** as the π‐conjugation scaffold (**Figure** **4**a). To achieve longer absorption and emission wavelengths along with enhanced photostability, a cyano‐phenyl group is introduced at the active site of the benzodithiophene tetra‐oxide acceptor. Additionally, to modulate the hydrophobicity of the molecule and impart LDs targeting capability, the alkyl side chains are adjusted to obtain the target fluorescent probe **Lipi‐PS**. The detailed synthesis and structural characterization of **Lipi‐PS** are described in the Supporting Information.

The photophysical properties of **Lipi‐PS** in solvents of varying polarities have been thoroughly characterized (Figure 4b; Figure S12 and Table S4, Supporting Information). The absorption spectra of **Lipi‐PS** are insensitive to solvent polarities, with an absorption peak ≈550 nm. In contrast, the emission spectra exhibit a pronounced solvatochromic effect. The emission wavelength maximum *λ*_em_ in *n*‐hexane is 617 nm, which red‐shifts to 758 nm in dioxane and further to 888 nm in dichloromethane as solvent polarity increasing. To quantify the polarity sensitivity of **Lipi‐PS**, a plot of *λ*_em_ versus the solvent polarity index *E*_T_(30) is constructed, and a linear fitting yields a polarity slope *K*_ps_ of 29.8 (Figure 4c). This value is ≈6.1 times higher than that of Nile Red (*K*_ps_ = 4.9) and represents the highest sensitivity among the reported polarity‐sensitive fluorescent probes to date (Table S5, SupportingbInformation). This indicates that **Lipi‐PS** exhibits ultrahigh sensitivity to spectral changes induced by polarity variations. Additionally, **Lipi‐PS** displays high fluorescence quantum yields, such as 38.6% in *n*‐hexane (*λ*_em_ = 617 nm), 30.1% in toluene (*λ*_em_ = 720 nm) and 7.7% in dioxane (*λ*_em_ = 758 nm). Achieving such high quantum yields in the NIR region is particularly noteworthy.

The HOMO and LUMO distributions of **Lipi‐PS** have been calculated (Figure S7, Supporting Information). Similar to molecule **12**, the HOMO of **Lipi‐PS** is primarily localized on the donor and π‐spacer regions, while the LUMO is predominantly situated on the acceptor. The overlap integral of the HOMO‐LUMO orbitals is 0.40, comparable to that of molecule **12**. The ground and excited‐state configurations of **Lipi‐PS** in cyclohexane and dioxane have been also calculated (Figure 4d). Consistent with the theoretical results for molecule **12**, the excited‐state configuration of **Lipi‐PS** exhibits high polarity sensitivity, with the twist angle (*θ*) between the acceptor and π‐spacer decreasing from 88.2° in cyclohexane to 46.8° in dioxane.

Fs‐TA spectroscopy has been performed on **Lipi‐PS** in cyclohexane and dioxane (Figure S13, Supporting Information), and the excited‐state relaxation pathways have been analyzed using global fitting and kinetic analysis (Figures S14 and S15, Supporting Information). In cyclohexane, two decay pathways are observed: pathway 1 involves a 3.8 ns LE decay process, while pathway 2 includes a 2.5 ps vibration relaxation of the ICT state, followed by a 0.56 ns ICT decay (Figure 4e). In dioxane, the direct decay of LE state is absent, leaving only the ICT decay process with significantly reduced lifetime: a 3.2 ps transition from LE state to ICT state, a 13.9 ps ICT vibration relaxation, and a 0.54 ns decay. These results are similar to those obtained for molecule **12**.

In short, the engineered probe **Lipi‐PS** establishes unprecedented polarity sensitivity benchmarks, demonstrating a 6.1‐fold enhancement over the gold‐standard Nile Red and surpassing all reported solvatochromic probes to date. This supersensitive response originates from unique excited‐state conformational dynamics, specifically polarity‐dependent modulation of twisted dihedral angles. Complementing its sensitivity, **Lipi‐PS** maintains exceptional NIR fluorescence quantum yields, delivering imaging‐capable brightness while minimizing phototoxicity.

### 2.4 LDs Staining Property of Lipi‐PS

Co‐localization fluorescence imaging of HeLa cells stained with **Lipi‐PS** and the representative LDs probe BODIPY 493/503 demonstrates that **Lipi‐PS** indeed selectively labels cellular LDs (Figure S16, Supporting Information). The LDs staining specificity of **Lipi‐PS**, in terms of the imaging signal‐to‐noise ratio (SNR), has been further evaluated and compared with Nile Red and BODIPY 493/503 (Figure S17, Supporting Information). Statistical analysis of multiple datasets (*n* = 30) reveals that Nile Red has a SNR of 2.6 ± 2.2, BODIPY 493/503 has a SNR of 3 ± 2.5, while **Lipi‐PS** exhibits a significantly higher SNR of 34.1 ± 13.9, which is over ten‐fold higher than the two classic probes. This clearly demonstrates that **Lipi‐PS** can specifically label LDs without nonspecific staining of other cellular fibrous structures, the working mechanism of selective labeling of LDs by organic fluorescent probe is based on the “similarity and compatibility principle,” the CLogP value of 6.8 for **Lipi‐PS** matches the appropriate LDs labeling values reported in the literature.^[^ ^36^, ^37^, ^38^ ^]^ Additionally, we have assessed the photostability of **Lipi‐PS**, which is crucial for obtaining high‐quality imaging results (Figure S18, Supporting Information). Using the same intense excitation laser (561 nm), we have compared the continuous imaging performance of **Lipi‐PS** and Nile Red. The fluorescence intensity of Nile Red decreases sharply with increasing numbers of exposures, retaining less than 3% of its initial signal after 100 consecutive confocal images. In contrast, **Lipi‐PS** maintains over 92% of its initial fluorescence intensity after 100 consecutive images, highlighting its exceptional photostability. The cytotoxicity of **Lipi‐PS** has been evaluated by MTT assay (Figure S19, Supporting Information). After incubating HeLa cells with 10 µm **Lipi‐PS** for 24 hours, the cell viability remains above 85%, indicating good biocompatibility. In fact, a staining concentration of 2 µm **Lipi‐PS** for 2 h is sufficient to provide high fluorescence intensity for imaging.

### 2.5 HSFI‐Based Polarity Mapping Methodology

**Figure 5:**
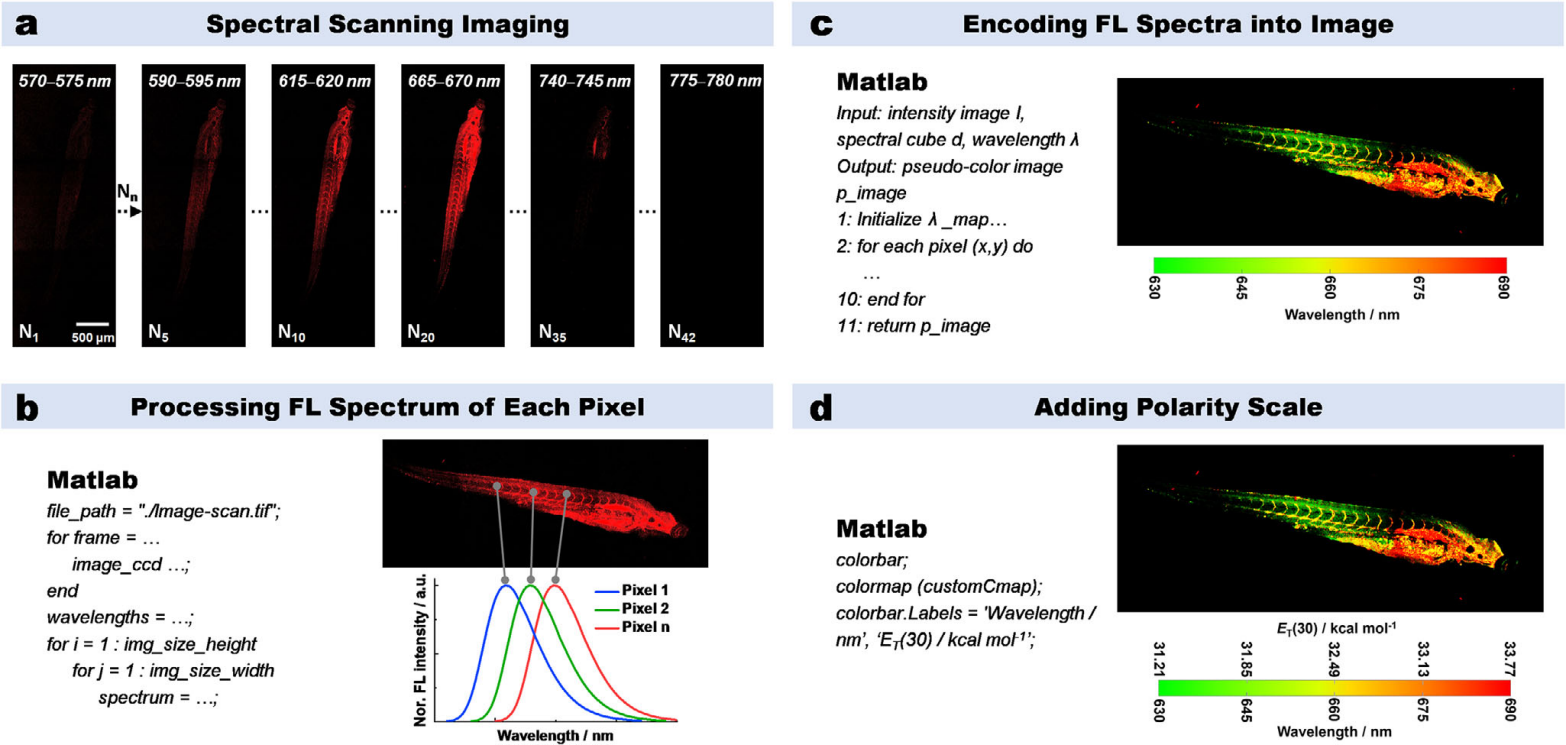
HSFI‐based polarity mapping methodology. a) Wavelength scanning imaging with confocal microscopy. b) The calculation of fluorescence spectra and peak wavelengths for each pixel by MATLAB. c) Encode the peak wavelengths of each pixel into the fluorescence intensity image. d) Add the polarity (*E*_T_(30)) scale to the HSFI image.

HSFI extends beyond the traditional fluorescence imaging by providing the spectral information for each pixel in addition to morphological details. By analyzing the shifts in fluorescence wavelength, HSFI can reflect the changes in polarity of the microenvironment surrounding the probe. In this study, polarity mapping using HSFI involves the following four steps. 1) Wavelength scanning imaging with confocal microscopy: the system performs continuous scans across the wavelength range of 570–780 nm with a step size of 5 nm or 10 nm (**Figure** **5**a). This process generates a series of images with varying fluorescence intensities, capturing the spectral information at each pixel. 2) The acquired wavelength‐scanned images are processed using MATLAB to determine the fluorescence spectra and peak wavelengths for each pixel (Figure 5b). This step extracts detailed spectral information from the raw imaging data. 3) The peak wavelengths of each pixel are encoded into the fluorescence intensity image in pseudocolor format (Figure 5c). This integration of spectral and morphological information results in HSFI, which simultaneously displays both structural and wavelength information. 4) To further enhance HSFI for polarity mapping, the wavelength and intensity detection errors of the confocal microscopy and the fluorescence spectrometer are calibrated (Table S6 and Figures S20–S22, Supporting Information). Based on the calibration and the fitted curve between fluorescence wavelength and polarity *E*_T_(30) obtained from the fluorescence spectrometer (Figure 4c), the wavelength scale is converted into a polarity index *E*_T_(30) scale (Figure 5d). This conversion enables direct visualization of polarity changes within the sample. Undoubtedly, given the subtle polarity changes in biological systems, it is essential that the spectral response of fluorescent probe is highly sensitive to these changes to achieve precise mapping of minute polarity variations. This is precisely the significance of polarity‐ultrasensitive probe **Lipi‐PS**.

### 2.6 HSFI Mapping LDs Polarity in Cells

**Figure 6:**
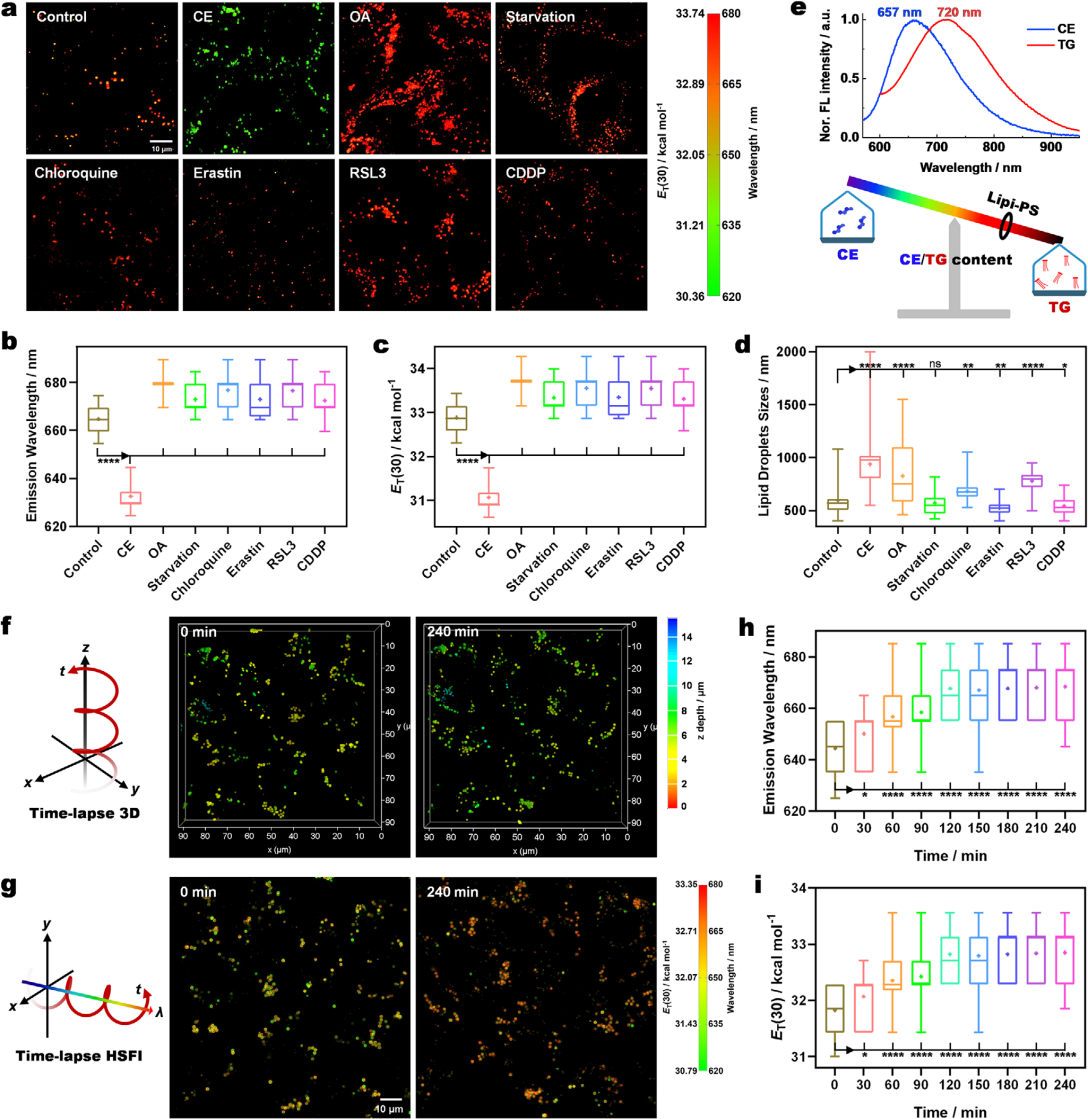
The polarity mapping of cellular LDs by HSFI. a) HSFI images of HeLa cells stained with **Lipi‐PS** under various stimulations; different colors represent different wavelengths/ polarities; scale bar: 10 µm. b–d) The statistic results of emission wavelength, polarity, and size of LDs in HeLa cells under various stimulations. e) Normalized fluorescence spectra of **Lipi‐PS** in CE and TG, as well as the mechanism diagram of the relationship between LDs composition and polarity/wavelength. f) Time‐lapse 3D confocal imaging (*xyzt*) of living HeLa cells stained with **Lipi‐PS** under the stimulation of OA; different colors represent different *Z*‐axis depths. g) Time‐lapse HSFI (*xyλt*) of living HeLa cells stained with **Lipi‐PS** under the stimulation of OA; different colors represent different wavelengths/polarities; scale bar: 10 µm. h,i) The statistic results of emission wavelength and polarity of LDs in time‐lapse HSFI. The sign of stars represents *P* value in *T*‐test.

HeLa cells stained with **Lipi‐PS** have been imaged using the HSFI polarity mapping method described above to obtain RGB fluorescence images, where different colors represent distinct wavelengths/polarities (**Figure** **6**a; Figures S23, S24, Supporting Information). In untreated HeLa cells, the majority of LDs exhibit fluorescence wavelengths *λ*_em_ ≈660 nm. Subsequently, various stimulations, including cholesterol, oleic acid (OA), and starvation, have been applied to alter the internal polarity of LDs, thereby verifying the sensitivity of **Lipi‐PS** to polarity changes in cellular LDs. The results show that after cholesterol treatment, the fluorescence wavelength of **Lipi‐PS** in LDs is significantly blue‐shifted, indicating a decrease in polarity. In contrast, other stimulations induce varying degrees of red‐shifts in fluorescence spectra, corresponding to increased polarity. To quantitatively assess the polarity changes, statistical analysis has been performed on multiple datasets (*n* = 40) from different treatments (Figure 6b,c). After cholesterol treatment, the fluorescence wavelength is shifted significantly from 664.8 ± 6.4 nm (control) to 632.5 ± 5.0 nm, with the polarity decreasing from 32.88 ± 0.36 to 31.06 ± 0.28 kcal mol^−1^. In contrast, OA treatment results in a redshift to 679.6 ± 4.5 nm, with an increased polarity of 33.72 ± 0.25 kcal mol^−1^. Other treatments, such as starvation (*λ*_em_ = 672.9 ± 6.0 nm, *E*_T_(30) = 33.34 ± 0.34 kcal mol^−1^), chloroquine (*λ*_em_ = 676.8 ± 6.8 nm, *E*_T_(30) = 33.56 ± 0.38 kcal mol^−1^), ferroptosis inducers Erastin (*λ*_em_ = 673.0 ± 7.3 nm, *E*_T_(30) = 33.35 ± 0.41 kcal mol^−1^), RSL3 (*λ*_em_ = 676.5 ± 6.8 nm, *E*_T_(30) = 33.55 ± 0.38 kcal mol^−1^) and CDDP (*λ*_em_ = 672.4 ± 6.7 nm, *E*_T_(30) = 33.31 ± 0.38 kcal mol^−1^), all induce red‐shifts and increased polarity. Regarding LDs size, the control group has the diameter of 596 ± 147 nm, which is significantly increased to 938 ± 251 nm after cholesterol treatment, 826 ± 285 nm after OA treatment, and 779 ± 90 nm after RSL3 treatment (Figure 6d). Other stimulations induce less significant changes in LDs size, with the smallest diameter observed after Erastin treatment (523 ± 70 nm).

To elucidate the causes of wavelength/polarity changes in LDs under different stimulations, we have simulated the LDs environment using cholesterol oleyl carbonate (cholesterol ester, CE) and trilaurin (triglyceride, TG). In CE, **Lipi‐PS** exhibits a fluorescence wavelength *λ*_em_ of 657 nm (*E*_T_(30) = 31.92 kcal mol^−1^), while in TG, its *λ*_em_ is red‐shifted to 720 nm (*E*_T_(30) = 34.04 kcal mol^−1^) (Figure 6e). Despite the small polarity difference between CE and TG, **Lipi‐ PS**’s high sensitivity to polarity changes accounts for the significant shift of *λ*_em_ up to 63 nm. These experiments demonstrate that the shift of *λ*_em_ of **Lipi‐PS** in HSFI could be able to reflect the changes in LDs composition: blue‐shifts indicate increased proportions of CEs, while red‐shifts correspond to increased proportions of TGs. This trend is consistent with the cellular HSFI results, where cholesterol treatment induces blue‐shifts and OA treatment induces red‐shifts. Thus, HSFI not only maps polarity changes but also provides insights into the composition of LDs (CE and TG proportions).

Beyond HeLa cells, **Lipi‐PS** has been applied to other cell lines, including HepG2, HT22 and 7702 cells (Figure S25a, Supporting Information). HSFI RGB images reveal that cancer cells (HeLa and HepG2) exhibit a predominantly yellow‐green fluorescence, while normal cells (HT22 and 7702) display an orange‐red fluorescence. Statistical analysis shows that the *λ*_em_ in LDs of HeLa, HepG2, HT22, and 7702 cells are 663.0 ± 6.3, 665.0 ± 10.5, 676.0 ± 12.9 and 677.0 ± 4.2 nm, respectively, with corresponding polarities of 32.62 ± 0.27, 32.71 ± 0.45, 33.18 ± 0.55 and 33.22 ± 0.18 kcal mol^−1^ (Figure S25b,c, Supporting Information). These results indicate that LDs in cancer cells have lower polarity compared to normal cells, likely due to higher proportions of CEs. Therefore, HSFI with **Lipi‐PS** holds promise for distinguishing tumor boundaries from normal tissues.

To further explore LDs dynamics, we have performed time‐lapse HSFI (*xyλt*) to track polarity changes in living cells and combined it with time‐lapse 3D imaging (*xyzt*) to quantify LDs number and size. In the experiment, HeLa cells treated with cholesterol for 48 hours were stained with **Lipi‐PS** for 2 hours and then stimulated with OA (400 µm) at the start of imaging. First, the number and size of LDs were tracked using 3D imaging (Figure 6f; Figure S26a, Supporting Information), followed by HSFI to quantify LDs polarity at the mid focal plane of 3D imaging (Figure 6g; Figure S27, Supporting Information). These two imaging modes were alternated every 30 min over a 4 h period (nine cycles, 657 frames), with no significant photobleaching observed, highlighting **Lipi‐PS**’s excellent photostability. Statistical analysis of the time‐lapse 3D imaging data reveals that the number of LDs is increased significantly within the first 60 min of OA stimulation due to the formation of new small LDs (Figure S26b,c, Supporting Information). Subsequently, the number is fluctuated slightly, likely due to the fusion or fission events. Regarding LDs size, the average diameter is decreased from 1.3 ± 0.6 to 1.2 ± 0.5 µm within the first 60 min and then stabilized ≈1.1 ± 0.5 µm. Concurrently, time‐lapse HSFI is conducted to track the wavelength/polarity changes of LDs. Initially, the RGB images are predominantly green, with the *λ*_em_ of 644.3 ± 10.2 nm and the *E*_T_(30) value of 31.83 ± 0.43 kcal mol^−1^ (Figure 6h,i). As adding OA, the images are gradually turned red, with the *λ*_em_ red‐shifting and polarity increasing. After 120 min, the *λ*_em_ is stabilized at 667.7 ± 9.8 nm, with a polarity of 32.82 ± 0.42 kcal mol^−1^. These results indicate that OA rapidly increases the proportion of TGs in LDs, with the CE/TG ratio stabilizing after 2 h. In overall, by integrating two 4D imaging approaches (*xyzt* and *xyλt*), we have simultaneously tracked the changes in LDs number, size, polarity, and composition in living cells, thus providing a powerful tool for elucidating LDs metabolism and dynamics.

### 2.7 HSFI Mapping LDs Polarity in Tissues

HSFI with **Lipi‐PS** has been employed to map the polarity of LDs in liver tissues of mouse models of non‐alcoholic fatty liver disease (NAFLD). The liver tissue sections from normal mice and mice fed a high‐fat diet for two and four weeks have been imaged by HSFI (Figure S28a, Supporting Information). Statistical analysis reveals that LDs in normal liver tissue exhibit a *λ*_em_ of 684.3 ± 13.4 nm, with a polarity of 33.53 ± 0.57 kcal mol^−1^ (Figure S28b,c, Supporting Information). In contrast, LDs in the liver tissues of NAFLD model mice exhibit a gradual blue‐shift in *λ*_em_ and a decrease in polarity: after two weeks, the *λ*_em_ is blue‐shifted to 677.0 ± 4.1 nm with a polarity of 33.22 ± 0.17 kcal mol^−1^; after four weeks, it is further blue‐shifted to 675.0 ± 2.6 nm with a polarity of 33.13 ± 0.11 kcal mol^−1^. Concurrently, the sizes of LDs in liver tissues significantly increase during this process: from 1.2 ± 0.6 µm in normal tissue to 1.9 ± 0.6 µm after two weeks of high‐fat feeding, and to 6.8 ± 3.9 µm after four weeks (Figure S28d, Supporting Information). These findings indicate that as LDs enlarging during NAFLD development, the proportion of CEs would be increased, leading to a gradual decrease in LDs polarity. Further investigation into the correlation between LDs size and polarity in the four‐week NAFLD liver tissues reveals the following: LDs with diameters less than 1 µm exhibit a *λ*_em_ of 680.2 ± 9.2 nm and a polarity of 33.36 ± 0.39 kcal mol^−1^; those with diameters between 1–3 µm show a blue‐shifted *λ*_em_ of 677.0 ± 5.8 nm and a polarity of 33.22 ± 0.25 kcal mol^−1^; and LDs larger than 3 µm have a further blue‐shifted *λ*_em_ of 675.0 ± 0.0 nm and a polarity of 33.13 ± 0.00 kcal mol^−1^ (Figure S28e,f, Supporting Information). This suggests that larger LDs in NAFLD tissues have lower polarity (i.e., higher CEs content) and exhibit more uniform polarity among different LDs. In contrast, smaller LDs have higher polarity (i.e., higher TGs content) with greater variability in polarity among individual LDs. In other words, there is a certain correlation between LDs size and composition.

To further analyze the distribution of LDs in normal liver and NAFLD tissues, two‐photon 3D imaging has been performed using **Lipi‐PS** (Figure S29a,b, Supporting Information). The two‐photon absorption cross‐section of **Lipi‐PS** has been measured to be as high as 1391 GM, significantly higher than most molecular fluorescent probes, indicating its strong two‐photon absorption capability.^[^ ^29^ ^]^ High‐quality 3D image of normal liver tissue has been obtained by two‐photon imaging at 980 nm excitation with 185 *Z*‐stack slices (500 nm step). In a similar manner, NAFLD tissue has been also imaged. Statistical analysis of LDs diameter distribution reveals that most LDs in both types of liver tissues have diameters within the range of 0.2–3 µm (Figure S29c, Supporting Information). However, the proportion of large LDs with diameters greater than 3 µm is significantly higher in NAFLD tissue (Figure S29d, Supporting Information). Consequently, the average volume of LDs in NAFLD tissue (14.10 ± 5.82 µm^3^) is 26 times larger than that in normal liver tissue (0.55 ± 0.05 µm^3^) (Figure S29e, Supporting Information). The density of LDs is also quantified, with 0.59 LD per 1000 µm^3^ in normal tissue and 0.83 LD per 1000 µm^3^ in NAFLD tissue, representing a 1.4‐fold increase (Figure S29f, Supporting Information). Given the increased average volume and density, the total volume fraction of LDs in NAFLD tissue (1.18%) is 37 times higher than that in normal tissue (0.032%) (Figure S29g, Supporting Information). The application of **Lipi‐PS** in both HSFI and two‐photon 3D imaging not only demonstrates its excellent performance but also provides a powerful multi‐dimensional tool for NAFLD research, offering detailed information on LDs polarity, composition, size, number and density.

### 2.8 HSFI Mapping the Polarity of Zebrafish

**Figure 7:**
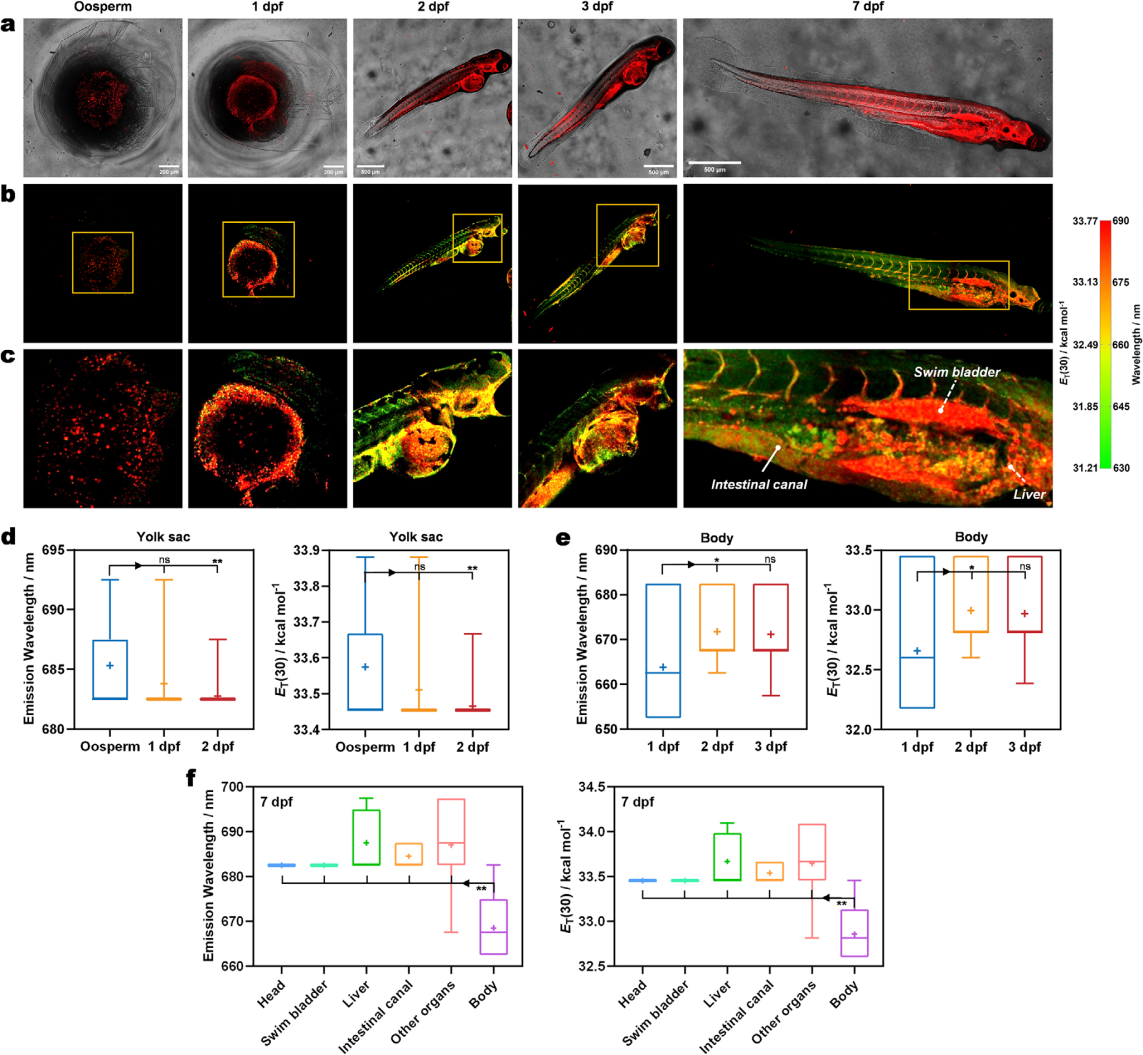
The polarity mapping of zebrafish across different developmental stages by HSFI. a) The merged images of bright field and fluorescence channels of zebrafishes stained by **Lipi‐PS** across different developmental stages. b,c) The corresponding HSFI images of zebrafishes and the enlarged views of square‐marked regions. d,e) The statistic results of emission wavelength and polarity of yolk sac and body in zebrafish on various dpf. f) The statistic results of emission wavelength and polarity of various organs in zebrafish on 7 dpf. The sign of stars represents *P* value in *T*‐test.

The successful application of **Lipi‐PS** in tissue imaging has motivated us to extend its use to intravital HSFI to map the polarity distribution of zebrafish across different developmental stages, from oosperm to juvenile fish and further to the gradual formation of organs. As an organism with high genetic homology to humans, studying the changes in intracorporeal microenvironmental polarity during zebrafish development holds significant importance.^[^ ^39^ ^]^ Here, we first monitored the development of zebrafish from oosperm to 7 days post fertilization (dpf) using bright‐field and fluorescence imaging (**Figure** 7a; Figure S30–S34, Supporting Information). Initially, the spherical oosperms, enclosed by a transparent chorion, is developed a curved body surrounding the yolk sac after one day, with embryonic structures clearly visible. On 2 dpf, most embryos hatch into fishes, with distinct head structures and a predominantly yolk sac‐based abdomen in a straight state. Subsequently, the juvenile fishes grow rapidly in length. On 7 dpf, the yolk sac has significantly diminished in size, the abdominal features begin to diversify, the swim bladder gradually inflate, and the intestinal canal become distinguishable. Simultaneously, we visualize the polarity distribution of zebrafishes stained with **Lipi‐PS** at different developmental stages using HSFI, presenting the results in RGB images and conducting statistical analysis of polarity changes in the yolk sac, body, and other organs (Figure 7b,c). From the oosperm (0 dpf) to 2 dpf of development, the yolk sac shrinks considerably, with *λ*_em_ blue‐shifting from 685.3 ± 3.9 to 682.8 ± 1.1 nm, corresponding to a decrease in polarity from 33.58 ± 0.17 to 33.47 ± 0.05 kcal mol^−1^ (Figure 7d). As the yolk sac diminishing, the body of the zebrafishes is developed progressively. Between 1 dpf and 3 dpf, the overall *λ*_em_ of body is red‐shifted from 663.8 ± 12.9 nm (polarity of 32.66 ± 0.55 kcal mol^−1^) to 671.2 ± 8.1 nm (polarity of 32.97 ± 0.35 kcal mol^−1^) (Figure 7e). On 7 dpf, when organ features become more pronounced, the head and swim bladder exhibit uniform polarity values of 33.45 kcal mol^−1^ (682.5 nm), while the liver, intestinal canal, and other organs have slightly higher polarities of 33.67 ± 0.30 kcal mol^−1^ (687.5 ± 7.1 nm), 33.54 ± 0.12 kcal mol^−1^ (684.5 ± 2.7 nm) and 33.65 ± 0.40 kcal mol^−1^ (687.0 ± 9.3 nm), respectively (Figure 7f). In contrast, the body has the lowest polarity at 32.86 ± 0.35 kcal mol^−1^ (668.5 ± 8.2 nm). Overall, throughout the developmental period, the body consistently exhibits the lowest polarity in zebrafishes, remaining lower than that of other organs, while the yolk sac maintains a relatively high polarity. The precise tracking of polarity changes across different developmental stages of zebrafishes underscores the advanced capabilities of **Lipi‐PS** for HSFI and provides valuable insights into the study of in vivo microenvironmental polarity dynamics.

### 2.9 A Special HSFI: *xyzλt* 5D‐Dynamic Tracking of Single LD

**Figure 8:**
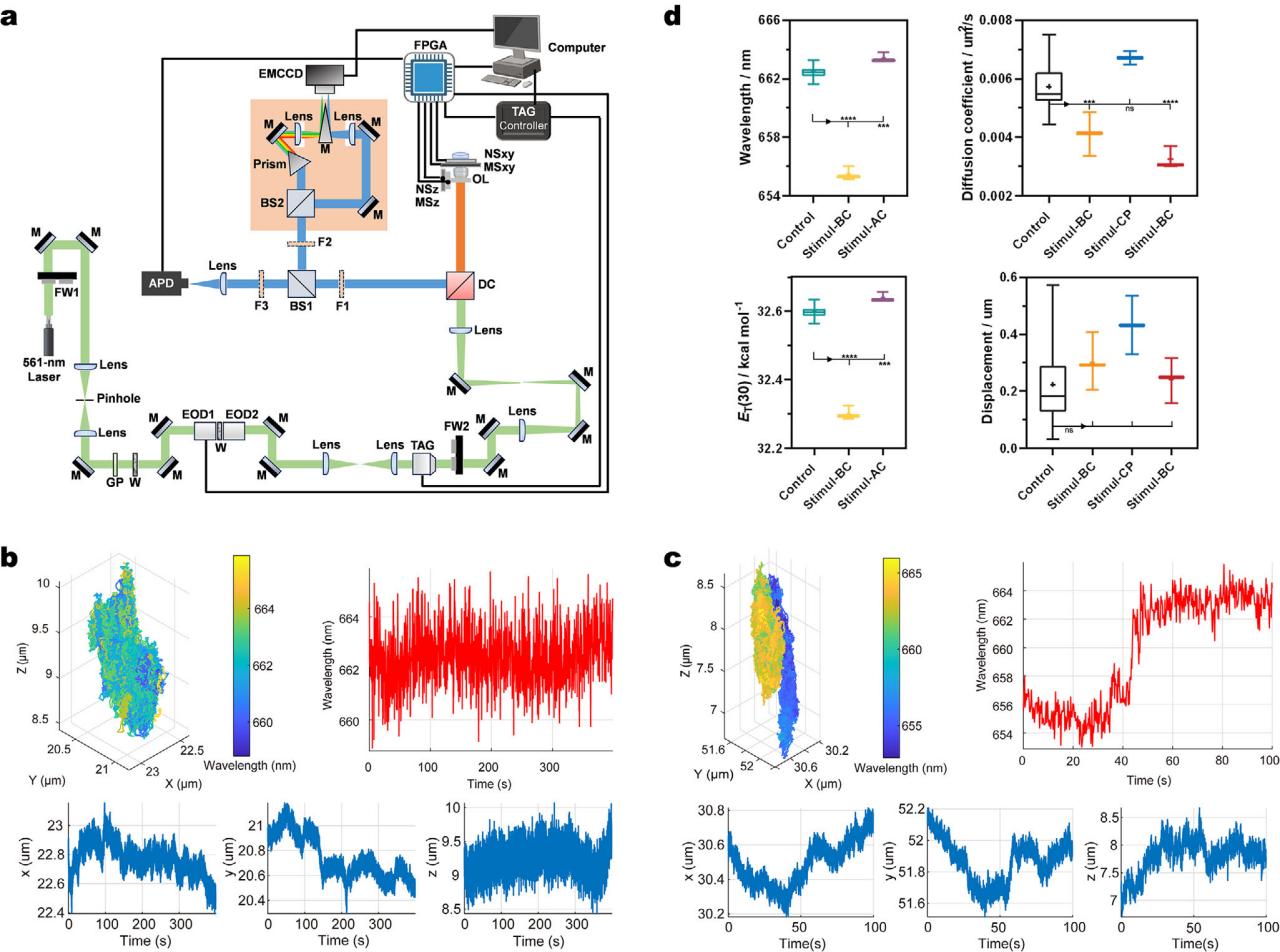
A special HSFI of **Lipi‐PS**: *xyzλt* 5D‐dynamic tracking of single LD. a) The optical layout of 3D‐SpecDIM system. b,c) The 3D trajectory and wavelength variations of single LD of living HeLa cells: control (left) and stimulated (right) groups. The 3D trajectory is integrated from the *xyz* positions over time. The wavelength information is encoded into pseudo‐colors and displayed in 3D trajectory. d) The statistic results of emission wavelength, polarity, average diffusion coefficient and average diffusion displacement of single LD: control group and stimulated group of various stages (BC: before change; CP: change process; AC: after change). The sign of stars represents *P* value in *T*‐test.

Recently, we have developed a 3D single molecule fluorescence spectral dynamics imaging microscopy (3D‐SpecDIM) capable of rapidly, accurately and sensitively tracking both 3D positional and fluorescence spectral changes simultaneously.^[^ ^34^ ^]^ Given **Lipi‐PS**’s specific LDs labeling ability, high photostability and ultrasensitivity to polarity, we have employed **Lipi‐PS** in 3D‐SpecDIM to realize a special HSFI of single LD. The optical layout of 3D‐SpecDIM is shown in **Figure** **8**a. The target LD is maintained within the excitation volume through the 3D single molecule active real‐time tracking system (3D‐SMART), avoiding issues such as defocusing and signal loss caused by rapid LDs movement during imaging. We have tracked LDs in two groups, the control group (HeLa cells without any stimulation) and the stimulated group (HeLa cells stimulated with cholesterol for 48 hours and then treated with OA before imaging), to investigate the effects of cholesterol and OA stimulation on the movement and polarity of individual LDs. Multiple LDs have been tracked in both groups, recording changes in *xyz* position and in *λ*_em_ over time. Then the positional data have been integrated into 3D trajectories, with *λ*_em_ changes encoded in pseudocolor, achieving the special HSFI of single LD. To the best of our knowledge, this represents the first‐ever 5D tracking at the single‐LD level.

Analysis of the tracking data reveals that LDs in the control group exhibit free diffusion within a confined region (Figure 8b; Figure S35a–c, Supporting Information), with an average diffusion coefficient of 5.7 × 10⁻^3^ ± 7.2 × 10⁻⁴ µm^2^ s^−1^ and an average diffusion distance of 0.22 ± 0.14 µm (Figure 8d). The *λ*_em_ of these LDs remain stable at 662.5 ± 0.4 nm, corresponding to a polarity of 32.60 ± 0.02 kcal mol^−1^, with no changes in wavelength or polarity during diffusion. In contrast, for the stimulated group, as shown in Figure 8c and Figure S35d–f (Supporting Information), a rapid increase in *λ*_em_ from 655.5 ± 0.5 to 663.4 ± 0.3 nm is observed within ≈20 s, accompanied by a corresponding increase in polarity from 32.30 ± 0.02 to 32.64 ± 0.01 kcal mol^−1^. Concurrent with this abrupt change in wavelength/polarity, a significant shift in the diffusion region is observed, indicating movement from one area to another.

These results highlight two important insights. First, the diffusion of OA or OA‐induced TGs from the cytoplasm to LDs is an extremely rapid process, occurring within 20 s. This finding contrasts with macroscopic observations (i.e., from a large number of LDs), suggesting that diffusion at the single‐LD level is fast, while the overall process appears slower due to the asynchronous nature of diffusion events across multiple LDs. Second, changes in LDs composition impart directionality to LDs diffusion, leading to shifts in diffusion regions, thereby demonstrating that composition influences dynamic behavior. To further investigate the impact of compositional changes on LDs dynamics, we have analyzed the diffusion coefficients and distances before, during, and after polarity changes in the stimulated group. Before polarity change, LDs exhibit free diffusion with an average diffusion coefficient of 4.1 × 10⁻^3^ ± 7.5 × 10⁻⁴ µm^2^ s^−1^ and an average diffusion distance of 0.30 ± 0.10 µm (Figure 8d). During the polarity change, the average diffusion coefficient significantly increases to 6.7 × 10⁻^3^ ± 3.3 × 10⁻⁴ µm^2^ s^−1^, with an average diffusion distance of 0.43 ± 0.15 µm and more directional diffusion. After the polarity increase, the average diffusion coefficient decreases to 3.3 × 10⁻^3^ ± 3.9 × 10⁻⁴ µm^2^ s^−1^, and the average diffusion distance reduces to 0.24 ± 0.08 µm, returning to a free diffusion state. These findings demonstrate that changes in LDs polarity (i.e., internal composition) significantly affect their diffusion behavior. The special HSFI of 3D‐SpecDIM with **Lipi‐PS** enables to study the correlation between LDs polarity and dynamic behavior at the single LD level. This approach provides a powerful tool for elucidating the interplay between LDs composition and movement, offering new insights into the biophysical properties of LDs in living cells.

## 3 Discussion

HSFI achieves functional equivalence to FLIM in microenvironmental polarity mapping while circumventing FLIM's limitations in system complexity and temporal resolution. Nevertheless, HSFI's development has been impeded by critical deficiencies in fluorescent probes—particularly the suboptimal spectral responsivity of existing polarity‐sensitive probes. We overcome this barrier through the creation of **Lipi‐PS**, a polarity‐ultrasensitive probe demonstrating unprecedented wavelength‐shift amplification. Its integration with HSFI enables multiscale polarity mapping across cellular, tissue, and whole‐organism levels (zebrafish models), establishing a robust methodological framework to unlock HSFI's full potential in dynamic biosystem analysis.

The main conclusions of this study include three parts. First, we have unraveled that the π‐spacer in D‐π‐A molecules plays a critical role in governing polarity sensitivity. For example, extending the π‐spacer from the commonly used phenyl group (molecule **1**) to a fluorene moiety (molecule **12**) can amplify polarity sensitivity by two‐fold. Theoretical calculations and fs‐TA studies have revealed that this enhanced sensitivity is attributed to the greater responsiveness of the excited‐state twisted conformation to solvent polarity. This finding provides important guidance for the development of new polarity‐sensitive probes, the introduction of new strong donor and acceptor groups, as well as the adjustment of the bonding modes and spatial configurations of molecules, is of great significance.

Second, we have successfully developed a novel fluorescent probe, **Lipi‐PS**, which exhibits the highest polarity sensitivity to date. Through systematic studies of the π‐spacer effect, careful selection of donor and acceptor groups, and consideration of LDs targeting specificity and photostability, we have rationally designed and synthesized the target fluorescent probe **Lipi‐PS**. Its polarity sensitivity is 6.1 times higher than that of the representative polarity‐sensitive probe Nile Red. Consequently, the *λ*_em_ of **Lipi‐PS** undergoes significant shifts in response to polarity changes. For instance, when transitioning from *n*‐hexane to dichloromethane, the *λ*_em_ of **Lipi‐PS** redshifts from 617 to 888 nm, resulting in a shift of up to 271 nm. This remarkable sensitivity allows the probe to detect even minor polarity changes in biological systems, enabling precise detection of polarity variations through HSFI.

Third, we have applied the polarity‐sensitive probe **Lipi‐PS** in HSFI to achieve polarity mapping in cells, tissues and zebrafish. As the first step, a strategy combining wavelength‐scanning imaging with image encoding has been proposed to simultaneously visualize morphological structures, fluorescence wavelengths and polarity, which refer to as HSFI for wavelength and polarity. Subsequently, we have established the relationship between wavelength/polarity and LDs composition, demonstrating that HSFI can also provide information on the compositional changes of CEs and TGs within LDs. After that, we have applied HSFI to cells, tissues and zebrafish models to showcase its utility. For example, time‐lapse HSFI imaging (*xyλt*) enables in situ tracking of polarity changes in LDs within living cells. When combined with time‐lapse 3D imaging (*xyzt*), this approach simultaneously provides information on the number and size of LDs. Additionally, HSFI is conducted to image the polarity of NAFLD liver tissues in mice and combine it with two‐photon 3D imaging to investigate the progression of NAFLD from multiple dimensions, including LDs polarity, composition, size, number and density. Furthermore, applying HSFI to zebrafish imaging allows us to precisely map polarity changes across different growth stages of zebrafish, providing valuable insights into polarity variations during organismal development. Moreover, we have applied **Lipi‐PS** in a special HSFI of 3D‐SpecDIM to achieve real‐time imaging and tracking of the movement trajectories and polarity changes of individual LD. To the best of our knowledge, this represents the first 5D (*xyzλt*) tracking report at the single‐LD level, enabling the study of the correlation between LDs polarity and dynamic behavior at this resolution.

Overall, this work involves the rational design and synthesis of a novel fluorescence wavelength‐ultrasensitive fluorescent probe, **Lipi‐PS**, and its applications in HSFI to simultaneously observe the morphology and reveal changes in LDs polarity and composition. Considering the immense potential of HSFI, which is still in its infancy, this work is expected to significantly advance its development and application, such as the combination with STED super‐resolution imaging and FLIM. We are also pleased to share **Lipi‐PS** in the research community to promote HSFI studies. With the continuous advancement of fluorescence wavelength‐ultrasensitive probes and HSFI methods, we believe HSFI has the potential to become a powerful tool in biomedical research, much like the widely used FLIM technology.

## 4 Experimental Section

Image encoding. Spectral fluorescence images were recorded using a Leica TCS SP8 confocal microscopy or a Zeiss LSM 980 confocal microscopy. After the spectral image sequence information is read using Matlab software, each pixel is traversed to obtain the maximum wavelength peak position information through Gaussian fitting. Subsequently, pseudocolor assignment is carried out. The color mapping and the range of wavelength variation are defined first. Then, a RGB image is created and the intensity information is overlaid. The plotting results are displayed after gamma correction and brightness adjustment, with wavelength and polarity scales added.

Animal experiment. The six‐week‐old normal Balb/c male mice and NAFLD model Balb/c male mice used in the experiment were purchased from Liaoning Changsheng Biotechnology Co. Ltd. The wild type AB strain zebrafishes of different developmental stages (0 dpf, 1 dpf, 2 dpf, 3 dpf, and 7 dpf) were developed from the same batch of oosperm and provided by Changchun Weishi Testing Technology Service Co. Ltd. All animal experiments were performed according to the guidelines of the Institutional Animal Care and Use Committee. The Institutional Animal Ethics Committee of Jilin University approved the animal experiments (license no. SY202502001, SY202503002). Mice were killed by anesthesia followed by cervical dislocation and then the corresponding liver tissues were sampled. The zebrafish after the experiment were killed in an ice bath.

## Conflict of Interest

The authors declare no conflict o f interest.

## Supporting information



Supporting Information

## Data Availability

The data that support the findings of this study are available from the corresponding author upon reasonable request.
