# Supplementary material for: Hyperspectral Fluorescence Imaging with a New Polarity‐Ultrasensitive Fluorescent Probe
Source: Adv Sci (Weinh). 2025 Jun 23;12(35):e08792. doi: 10.1002/advs.202508792 (PMC12463004; doi:10.1002/advs.202508792)
Supplement: Supplementary file 1 — Supporting Information [file ADVS-12-e08792-s001.pdf]

ADVANCED SCIENCE
Open Access

Supporting Information

for *Adv. Sci.*, DOI 10.1002/advs.202508792

Hyperspectral Fluorescence Imaging with a New Polarity-Ultrasensitive Fluorescent Probe

Ri Zhou, Hao Sha, Shengjie Fu, Guannan Liu, Jinbei Wei, Chenguang Wang, Shangguo Hou* and Geyu Lu**

Supporting Information For

Hyperspectral Fluorescence Imaging with A New Polarity-Ultrasensitive Fluorescent Probe

Ri Zhou,[#] Hao Sha,[#] Shengjie Fu,[#] Guannan Liu, Jinbei Wei, Chenguang Wang, Shangguo Hou,* Geyu Lu**

Dr. R. Zhou, S. Fu, Dr. G. Liu, Prof. J. Wei, Prof. C. Wang, Prof. G. Lu
State Key Laboratory of Integrated Optoelectronics (JLU Region), Key Laboratory of Advanced Gas Sensors of Jilin Province, College of Electronic Science and Engineering, Jilin University, Changchun 130012, China
E-mail: wangchenguang@jlu.edu.cn; lugy@jlu.edu.cn
H. Sha
School of Computer Science and Technology, Harbin Institute of Technology (Shenzhen), Shenzhen 518006, China
H. Sha, Prof. S. Hou
Institute of Systems and Physical Biology, Shenzhen Bay Laboratory, Shenzhen 518132, China
E-mail: shangguo.hou@szbl.ac.cn
R. Zhou, H. Sha, and S. Fu contributed equally to this work

This PDF file includes:

1. Experimental Details of Chemical Synthesis
2. Experimental Details of Photophysical Characterization
3. Experimental Details of Theoretical Calculations
4. Experimental Details of Cell, Tissue, and Animal Experiments
5. References of Supporting Information
6. Supporting Schemes, Figures and Tables
7. NMR and MS of Synthesized Molecules

1. Experimental Details of Chemical Synthesis

General. ^{1}H NMR and ^{13}C NMR spectra were recorded with a Zhongke-Niujin AS 400 (400 MHz for ^{1}H and 101 MHz for ^{13}C) or a Bruker AVANCEIII 500 spectrometer (500 MHz for ^{1}H and 126 MHz for ^{13}C) in $CDCl_3$, CD_2Cl_2 or DMSO-d_6. The chemical shifts in NMR spectra are reported in δ ppm using tetramethylsilane as an internal standard. HR-MALDI-TOF mass spectra were collected by an Autoflex III (Bruker Daltonics Inc.) MALDI-TOF spectrometer. All reactions were performed under a N_2 atmosphere, unless otherwise stated. Commercially available solvents and reagents were used without further purification unless otherwise mentioned.

Compound 1. This molecule was synthesized according to the method reported in the literature[1]. ^{1}H NMR (400 MHz, DMSO-d_6): δ 7.74 (d, J = 9.0 Hz, 2H), 7.69 (d, J = 7.0 Hz, 1H), 7.50 (s, 1H), 7.46 (d, J = 7.0 Hz, 1H), 6.81 (d, J = 9.1 Hz, 2H), 4.32–4.24 (m, 4H), 3.49–3.39 (m, 4H), 1.91–1.76 (m, 4H), 1.13 (t, J = 6.9 Hz, 6H), 1.07–0.98 (m, 6H).

Compound 2. To a suspension of 4-bromo-2,1,3-benzothiadiazole (0.150 g, 0.70 mmol), *N*,*N*-diethyl-4-(4,4,5,5-tetramethyl-1,3,2-dioxaborolan-2-yl)aniline (0.230 g, 0.84 mmol) and K_2CO_3 (0.289 g, 2.10 mmol) in toluene (16 mL), ethanol (2 mL) and water (2 mL) was added $Pd(PPh_3)_4$ (0.040 g, 0.035 mmol), and the resulting mixture was stirred at 90 °C for 18 h. After cooling to room temperature, water (100 mL) was added and the mixture was extracted with dichloromethane (200 mL). The organic layer was washed with brine (50 mL), and then dried over anhydrous $MgSO_4$, and filtered. After concentration of the filtrate under reduced pressure, the resulting mixture was purified by silica gel column chromatography to afford 0.100 g (0.352 mmol, 50%) of compound **2** as orange red powders. ^{1}H NMR (400 MHz, DMSO-d_6): δ 7.99–7.94 (m, 1H), 7.93–7.91 (m, 1H), 7.91–7.89 (m, 1H), 7.78–7.74 (m, 2H), 6.86–6.79 (m, 2H), 3.44 (q, J = 7.0 Hz, 4H), 1.16 (t, J = 7.0 Hz, 6H).

Compound 3. This molecule was synthesized according to the method reported in the literature[2]. ^{1}H NMR (400 MHz, $CDCl_3$): δ 9.31 (s, 1H), 8.27–8.01 (m, 4H), 7.84–7.64 (m, 2H), 6.99–6.69 (m, 2H), 3.59–3.39 (m, 4H), 1.28 (t, J = 6.3 Hz, 6H).

Compound 4. To a suspension of 4-bromo-*N*-methyl-1,8-naphthalimide (0.150 g, 0.52 mmol), *N*,*N*-diethyl-4-(4,4,5,5-tetramethyl-1,3,2-dioxaborolan-2-yl)aniline (0.171 g, 0.62 mmol) and K_2CO_3 (0.215 g, 1.56 mmol) in toluene (16 mL), ethanol (2 mL) and water (2 mL) was added $Pd(PPh_3)_4$ (0.030 g, 0.026 mmol), and the resulting mixture was stirred at 90 °C for 18 h. After cooling to room temperature, water (100 mL) was added and the mixture was extracted with dichloromethane (200 mL). The organic layer was washed with brine (50 mL),

and then dried over anhydrous $MgSO_4$, and filtered. After concentration of the filtrate under reduced pressure, the resulting mixture was purified by silica gel column chromatography to afford 0.125 g (0.35 mmol, 68%) of compound **4** as orange powders. ^{1}H NMR (400 MHz, DMSO-d_6): δ 8.57–8.49 (m, 2H), 8.45 (d, J = 8.4 Hz, 1H), 7.85 (t, J = 7.8 Hz, 1H), 7.76 (d, J = 7.6 Hz, 1H), 7.42 (d, J = 8.5 Hz, 2H), 6.88 (d, J = 8.6 Hz, 2H), 3.50–3.41 (m, 7H), 1.18 (t, J = 6.9 Hz, 6H).

Compound 5a. This molecule was synthesized according to the method reported in the literature[3]. ^{1}H NMR (400 MHz, DMSO-d_6): δ 8.00 (d, J = 7.5 Hz, 1H), 7.95 (s, 1H), 7.76–7.70 (m, 1H), 7.66–7.59 (m, 2H).

Compound 5. To a suspension of compound **5a** (0.150 g, 0.61 mmol), *N*,*N*-diethyl-4-(4,4,5,5-tetramethyl-1,3,2-dioxaborolan-2-yl)aniline (0.202 g, 0.73 mmol) and K_2CO_3 (0.253 g, 1.83 mmol) in toluene (16 mL), ethanol (2 mL) and water (2 mL) was added $Pd(PPh_3)_4$ (0.035 g, 0.031 mmol), and the resulting mixture was stirred at 90 °C for 18 h. After cooling to room temperature, water (100 mL) was added and the mixture was extracted with dichloromethane (200 mL). The organic layer was washed with brine (50 mL), and then dried over anhydrous $MgSO_4$, and filtered. After concentration of the filtrate under reduced pressure, the resulting mixture was purified by silica gel column chromatography to afford 0.086 g (0.27 mmol, 45%) of compound **5** as yellow powders. ^{1}H NMR (400 MHz, DMSO-d_6): δ 7.88–7.84 (m, 1H), 7.71–7.64 (m, 3H), 7.58 (s, 1H), 7.57–7.49 (m, 2H), 6.85–6.79 (m, 2H), 3.44 (q, J = 7.1 Hz, 4H), 1.14 (t, J = 7.0 Hz, 6H).

Compound C_1. This molecule was synthesized according to the method reported in the literature[4]. ^{1}H NMR (400 MHz, DMSO-d_6): δ 8.06 (s, 2H), 4.27 (t, J = 6.4 Hz, 4H), 1.85–1.75 (m, 4H), 1.00 (t, J = 7.4 Hz, 6H).

Compound 6. To a suspension of compound **C_1** (0.150 g, 0.28 mmol), *N*,*N*-dimethyl-4-(4,4,5,5-tetramethyl-1,3,2-dioxaborolan-2-yl)aniline (0.070 g, 0.34 mmol) and K_2CO_3 (0.170 g, 1.23 mmol) in toluene (16 mL), ethanol (2 mL) and water (2 mL) was added $Pd(PPh_3)_4$ (0.016 g, 0.014 mmol), and the resulting mixture was stirred at 90 °C for 18 h. After cooling to room temperature, water (100 mL) was added and the mixture was extracted with dichloromethane (200 mL). The organic layer was washed with brine (50 mL), and then dried over anhydrous $MgSO_4$, and filtered. After concentration of the filtrate under reduced pressure, the resulting mixture was purified by silica gel column chromatography to afford 0.042 g (0.086 mmol, 30%) of compound **6** as red powders. ^{1}H NMR (400 MHz, DMSO-d_6): δ 7.80 (d, J = 8.9 Hz, 2H), 7.72 (d, J = 7.0 Hz, 1H), 7.58 (s, 1H), 7.49 (d, J = 7.0 Hz, 1H), 6.86 (d, J = 9.1 Hz, 2H), 4.30 (t, J = 6.4 Hz, 4H), 3.05 (s, 6H), 1.91–1.80 (m, 4H), 1.08–1.01 (m, 6H).

Compound 7. To a suspension of compound $\mathbf{C_1}$ (0.150 g, 0.28 mmol), 1-(4-(4,4,5,5-tetramethyl-1,3,2-dioxaborolan-2-yl)phenyl)pyrrolidine (0.085 g, 0.31 mmol) and K_2CO_3 (0.170 g, 1.23 mmol) in toluene (16 mL), ethanol (2 mL) and water (2 mL) was added $Pd(PPh_3)_4$ (0.016 g, 0.014 mmol), and the resulting mixture was stirred at 90 °C for 18 h. After cooling to room temperature, water (100 mL) was added and the mixture was extracted with dichloromethane (200 mL). The organic layer was washed with brine (50 mL), and then dried over anhydrous $MgSO_4$, and filtered. After concentration of the filtrate under reduced pressure, the resulting mixture was purified by silica gel column chromatography to afford 0.035 g (0.068 mmol, 24%) of compound **7** as red powders. ^{1}H NMR (400 MHz, $CDCl_3$): δ 7.71 (d, J = 8.8 Hz, 2H), 7.38 (d, J = 6.9 Hz, 1H), 7.11 (s, 1H), 6.68–6.60 (m, 3H), 4.46–4.34 (m, 4H), 3.38 (t, J = 6.3 Hz, 4H), 2.06 (t, J = 6.4 Hz, 4H), 1.99–1.85 (m, 4H), 1.17–1.05 (m, 6H).

Compound 8. To a suspension of compound $\mathbf{C_1}$ (0.150 g, 0.28 mmol), *N*,*N*-diphenyl-4-(4,4,5,5-tetramethyl-1,3,2-dioxaborolan-2-yl)aniline (0.116 g, 0.31 mmol) and K_2CO_3 (0.117 g, 0.85 mmol) in toluene (16 mL), ethanol (2 mL) and water (2 mL) was added $Pd(PPh_3)_4$ (0.016 g, 0.014 mmol), and the resulting mixture was stirred at 90 °C for 18 h. After cooling to room temperature, water (100 mL) was added and the mixture was extracted with dichloromethane (200 mL). The organic layer was washed with brine (50 mL), and then dried over anhydrous $MgSO_4$, and filtered. After concentration of the filtrate under reduced pressure, the resulting mixture was purified by silica gel column chromatography to afford 0.068 g (0.11 mmol, 39%) of compound **8** as red powders. ^{1}H NMR (400 MHz, DMSO-d_6): δ 7.83 (d, J = 8.7 Hz, 2H), 7.74 (d, J = 7.0 Hz, 2H), 7.53 (d, J = 7.0 Hz, 1H), 7.41 (t, J = 7.8 Hz, 4H), 7.23–7.13 (m, 6H), 7.02 (d, J = 8.7 Hz, 2H), 4.31 (t, J = 6.2 Hz, 4H), 1.91–1.78 (m, 4H), 1.04 (t, J = 7.4 Hz, 6H).

Compound 9. To a suspension of compound $\mathbf{C_1}$ (0.150 g, 0.28 mmol), 9-(4-(4,4,5,5-tetramethyl-1,3,2-dioxaborolan-2-yl)phenyl)-9H-carbazole (0.114 g, 0.31 mmol) and K_2CO_3 (0.117 g, 0.85 mmol) in toluene (16 mL), ethanol (2 mL) and water (2 mL) was added $Pd(PPh_3)_4$ (0.016 g, 0.014 mmol), and the resulting mixture was stirred at 90 °C for 18 h. After cooling to room temperature, water (100 mL) was added and the mixture was extracted with dichloromethane (200 mL). The organic layer was washed with brine (50 mL), and then dried over anhydrous $MgSO_4$, and filtered. After concentration of the filtrate under reduced pressure, the resulting mixture was purified by silica gel column chromatography to afford 0.061 g (0.098 mmol, 35%) of compound **9** as orange powders. ^{1}H NMR (400 MHz, $CDCl_3$): δ 8.20 (d, J = 7.7 Hz, 2H), 8.13 (d, J = 8.5 Hz, 2H), 7.80 (d, J = 8.4 Hz, 2H), 7.59 (s, 1H), 7.54 (d, J = 8.2 Hz, 2H), 7.48 (t, J = 7.4 Hz, 3H), 7.37 (t, J = 7.4 Hz, 2H), 6.77 (d, J = 6.9 Hz, 1H), 4.52 (q, J = 6.2 Hz, 4H), 2.05–1.95 (m, 4H), 1.19 (q, J = 7.2 Hz, 6H).

Compound 10a. This molecule was synthesized according to the method reported in the literature[5]. ^{1}H NMR (400 MHz, DMSO-d_6): δ 7.90 (d, J = 1.7 Hz, 1H), 7.68 (d, J = 9.1 Hz, 1H), 7.58 (d, J = 8.8 Hz, 1H), 7.41–7.35 (m, 1H), 7.21–7.15 (m, 1H), 6.89 (d, J = 2.3 Hz, 1H), 3.44 (q, J = 7.0 Hz, 4H), 1.14 (t, J = 7.0 Hz, 6H).

Compound 10-pin. To a suspension of 6-bromo-*N*,*N*-diethyl-2-naphthalenamine (1.00 g, 3.59 mmol), bis(pinacolato)diborane (1.10 g, 4.33 mmol), potassium acetate (1.60 g, 16.3 mmol) in dry 1,4-dioxane (50 mL) was added Pd(dppf)Cl_2 (0.132 g, 0.18 mmol), and the resulting mixture was stirred at 90 °C for 18 h. After cooling to room temperature, water (100 mL) was added and the mixture was extracted with dichloromethane (200 mL). The organic layer was washed with brine (50 mL), and then dried over anhydrous $MgSO_4$, and filtered. After concentration of the filtrate under reduced pressure, the resulting mixture was purified by silica gel column chromatography to afford 0.665 g (2.05 mmol, 57%) of compound **10-pin** as white powders. ^{1}H NMR (400 MHz, $CDCl_3$): δ 8.21 (s, 1H), 7.73 (t, J = 8.2 Hz, 2H), 7.60 (d, J = 8.1 Hz, 1H), 7.10 (d, J = 8.8 Hz, 1H), 6.86 (s, 1H), 3.50 (q, J = 6.7 Hz, 4H), 1.41 (s, 12H), 1.26 (t, J = 6.8 Hz, 6H).

Compound 10. To a suspension of compound **C_1** (0.150 g, 0.28 mmol), compound **10-pin** (0.100 g, 0.31 mmol) and K_2CO_3 (0.117 g, 0.85 mmol) in toluene (16 mL), ethanol (2 mL) and water (2 mL) was added $Pd(PPh_3)_4$ (0.016 g, 0.014 mmol), and the resulting mixture was stirred at 90 °C for 18 h. After cooling to room temperature, water (100 mL) was added and the mixture was extracted with dichloromethane (200 mL). The organic layer was washed with brine (50 mL), and then dried over anhydrous $MgSO_4$, and filtered. After concentration of the filtrate under reduced pressure, the resulting mixture was purified by silica gel column chromatography to afford 0.062 g (0.11 mmol, 38%) of compound **10** as brown powders. ^{1}H NMR (400 MHz, DMSO-d_6): δ 8.23 (s, 1H), 7.92–7.85 (m, 3H), 7.81–7.73 (m, 2H), 7.53 (d, J = 6.8 Hz, 1H), 7.27–7.22 (m, 1H), 6.98 (s, 1H), 4.38–4.32 (m, 4H), 3.56–3.49 (m, 4H), 1.94–1.83 (m, 4H), 1.20 (t, J = 6.9 Hz, 6H), 1.08 (t, J = 7.3 Hz, 6H).

Compound 11a. This molecule was synthesized according to the method reported in the literature[6]. ^{1}H NMR (400 MHz, DMSO-d_6): δ 7.57–7.51 (m, 4H), 7.48 (d, J = 8.8 Hz, 2H), 6.72 (d, J = 8.8 Hz, 2H), 3.37 (q, J = 7.0 Hz, 4H), 1.10 (t, J = 7.0 Hz, 6H).

Compound 11-pin. To a suspension of 4′-bromo-*N*,*N*-diethyl[1,1′-biphenyl]-4-amine (1.00 g, 3.29 mmol), bis(pinacolato)diborane (1.00 g, 3.95 mmol), potassium acetate (1.60 g, 16.3 mmol) in dry 1,4-dioxane (50 mL) was added Pd(dppf)Cl_2 (0.132 g, 0.18 mmol), and the resulting mixture was stirred at 90 °C for 18 h. After cooling to room temperature, water (100 mL) was added and the mixture was extracted with dichloromethane (200 mL). The organic

layer was washed with brine (50 mL), and then dried over anhydrous $MgSO_4$, and filtered. After concentration of the filtrate under reduced pressure, the resulting mixture was purified by silica gel column chromatography to afford 0.589 g (1.68 mmol, 51%) of compound **11-pin** as white powders. ^{1}H NMR (400 MHz, DMSO-d_6): δ 7.68 (d, J = 8.0 Hz, 2H), 7.59 (d, J = 8.1 Hz, 2H), 7.53 (d, J = 8.8 Hz, 2H), 6.73 (d, J = 8.8 Hz, 2H), 3.37 (q, J = 6.9 Hz, 4H), 1.30 (s, 12H), 1.11 (t, J = 7.0 Hz, 6H).

Compound 11. To a suspension of compound **C$_1$** (0.150 g, 0.28 mmol), compound **11-pin** (0.109 g, 0.31 mmol) and K_2CO_3 (0.117 g, 0.85 mmol) in toluene (16 mL), ethanol (2 mL) and water (2 mL) was added $Pd(PPh_3)_4$ (0.016 g, 0.014 mmol), and the resulting mixture was stirred at 90 °C for 18 h. After cooling to room temperature, water (100 mL) was added and the mixture was extracted with dichloromethane (200 mL). The organic layer was washed with brine (50 mL), and then dried over anhydrous $MgSO_4$, and filtered. After concentration of the filtrate under reduced pressure, the resulting mixture was purified by silica gel column chromatography to afford 0.053 g (0.087 mmol, 31%) of compound **11** as brown powders. ^{1}H NMR (400 MHz, DMSO-d_6): δ 8.01–7.93 (m, 3H), 7.82 (d, J = 8.6 Hz, 2H), 7.76 (d, J = 6.9 Hz, 1H), 7.65 (d, J = 8.8 Hz, 2H), 7.55 (d, J = 7.2 Hz, 1H), 6.79 (d, J = 8.4 Hz, 2H), 4.38–4.30 (m, 4H), 3.46–3.39 (m, 4H), 1.92–1.82 (m, 4H), 1.15 (t, J = 6.9 Hz, 6H), 1.07 (t, J = 7.3 Hz, 6H).

Compound 12-pin. This molecule was synthesized according to the method reported in the literature[7]. ^{1}H NMR (400 MHz, $CDCl_3$): δ 7.79 (s, 1H), 7.75 (d, J = 7.5 Hz, 1H), 7.57 (t, J = 7.1 Hz, 2H), 6.71 (d, J = 2.0 Hz, 1H), 6.68–6.64 (m, 1H), 3.43 (q, J = 7.0 Hz, 4H), 1.47 (s, 6H), 1.37 (s, 12H), 1.21 (t, J = 7.0 Hz, 6H).

Compound 12. To a suspension of compound **C$_1$** (0.150 g, 0.28 mmol), *N*,*N*-diethyl-9,9-dimethyl-7-(4,4,5,5-tetramethyl-1,3,2-dioxaborolan-2-yl)-9H-fluoren-2-amine (0.121 g, 0.31 mmol) and K_2CO_3 (0.117 g, 0.85 mmol) in toluene (16 mL), ethanol (2 mL) and water (2 mL) was added $Pd(PPh_3)_4$ (0.016 g, 0.014 mmol), and the resulting mixture was stirred at 90 °C for 18 h. After cooling to room temperature, water (100 mL) was added and the mixture was extracted with dichloromethane (200 mL). The organic layer was washed with brine (50 mL), and then dried over anhydrous $MgSO_4$, and filtered. After concentration of the filtrate under reduced pressure, the resulting mixture was purified by silica gel column chromatography to afford 0.047 g (0.073 mmol, 26%) of compound **12** as atropurpureus powders. ^{1}H NMR (400 MHz, CD_2Cl_2): δ 7.82 (s, 2H), 7.67 (s, 2H), 7.48 (d, J = 7.3 Hz, 2H), 6.86–6.66 (m, 3H), 4.45 (t, J = 6.2 Hz, 4H), 3.51 (s, 4H), 2.04–1.96 (m, 4H), 1.56 (s, 6H), 1.27 (t, J = 6.6 Hz, 6H), 1.20–1.15 (m, 6H).

Compound C_2. This molecule was synthesized according to the method reported in the literature[1]. ^{1}H NMR (400 MHz, $CDCl_3$): δ 7.65 (s, 2H), 4.53 (s, 4H), 3.76 (s, 4H), 3.44 (s, 6H).

Compound Lipi-PS. To a suspension of compound **C_2** (0.650 g, 1.16 mmol), compound **12-pin** (0.499 g, 1.28 mmol) and K_2CO_3 (0.480 g, 3.48 mmol) in toluene (32 mL), ethanol (4 mL) and water (4 mL) was added $Pd(PPh_3)_4$ (0.032 g, 0.028 mmol), and the resulting mixture was stirred at 90 °C for 12 h. After cooling to room temperature, 4-(4,4,5,5-tetramethyl-1,3,2-dioxaborolane-2-yl)benzonitrile (0.069 g, 0.30 mmol), K_2CO_3 (0.084 g, 0.60 mmol) and $Pd(PPh_3)_4$ (0.016 g, 0.014 mmol) were added and the mixture was stirred at 90 °C for 18 h. After cooling to room temperature, water (100 mL) was added and the mixture was extracted with dichloromethane (200 mL). The organic layer was washed with brine (50 mL), and then dried over anhydrous $MgSO_4$, and filtered. After concentration of the filtrate under reduced pressure, the resulting mixture was purified by silica gel column chromatography to afford 0.083 g (0.11 mmol, 9%) of compound **Lipi-PS** as black powders. ^{1}H NMR (500 MHz, CD_2Cl_2): δ 7.99 (s, 1H), 7.98–7.95 (m, 2H), 7.86–7.82 (m, 3H), 7.82–7.76 (m, 2H), 7.69 (d, J = 7.3 Hz, 1H), 7.64 (d, J = 7.6 Hz, 1H), 6.80–6.69 (m, 2H), 4.68–4.60 (m, 4H), 3.89–3.84 (m, 4H), 3.57–3.45 (m, 10H), 1.55 (s, 6H), 1.26 (t, J = 7.1 Hz, 6H). HRMS (MALDI-TOF): *m/z* calcd. for $C_{42}H_{42}N_2O_8S_2$: 766.2377; found: 766.2381.

2. Experimental Details of Photophysical Characterization

Measurements. UV-vis absorption spectra of solutions were measured with a Shimadzu UV-2550 spectrometer. Emission spectra of solutions were measured with an Ocean QE Pro fiber optic spectrometer, a Leica TCS SP8 confocal microscopy or a Zeiss LSM 980 confocal microscopy. Absolute fluorescence quantum yields (Φ_F) of solutions were determined with an Ocean QE Pro fiber optic spectrometer equipped with a calibrated integrating sphere system. Femtosecond transient absorption was measured using a HELIOS FIRE transient absorption spectrometer with the excitation of 405 nm and the detection of 420–750 nm.

3. Experimental Details of Theoretical Calculations

Methods. The time-dependent density functional theory (TD-DFT) calculation was performed using Gaussian 16 program at the B3LYP/6-31G* level of theory. The first excited state of molecules **1**, **10**, **11**, **12** and **Lipi-PS** correspond to the transition of HOMOs to LUMOs. The HOMO−LUMO overlap integral was calculated using the Multiwfn package for quantifying the degree of orbital separation.

4. Experimental Details of Cell, Tissue, and Animal Experiments

Cell culture. In general, HeLa, HepG2, and HT22 cells were cultured in Dulbecco's modified Eagle's medium (DMEM, high glucose, pyruvate) containing 10% fetal bovine serum (FBS) and 1% Antibiotic-Antimycotic (AA) at 37 °C in a humidified 5% (vol/vol) CO_2 incubator. The culture condition of 7702 cells was to replace DMEM medium with RPMI (Roswell Park Memorial Institute) 1640 medium, and other conditions remained unchanged.

Cell viability assays. The effect of probe **Lipi-PS** on cell viability was analyzed by using 3-(4,5-dimethylthiazol-2-yl)-2,5-diphenyltetrazolium bromide (MTT). HeLa cells were seeded into a flat-bottomed 96-well plate (1×10^4 cells/well) and incubated in DMEM containing 10% FBS (DMEM+) at 37 °C in a 5% CO_2 incubator for 24 h. The medium was then replaced with a culture medium DMEM+ containing various concentrations of **Lipi-PS** (0, 0.5, 1.0, 2.0, 5.0 and 10.0 μM) and 1% DMSO. After incubation for 24 h, MTT reagent (final concentration, 0.5 mg/mL) was added to each well, and the plates were incubated for another 4 h in a CO_2 incubator. Excess MTT tetrazolium solution was then removed. After the formazan crystals were solubilized in DMSO (100 μL/well) for 30 min at room temperature, the absorbance of each well was measured by a microplate reader (Bio-Tek Instruments, Inc) with an excitation at 530 nm.

Co-staining experiments. HeLa cells (5×10^4) were seeded in glass-bottom dishes two days before imaging. After removal of the medium, the cells were stained in DMEM+ containing probes (2 μM **Lipi-PS**, 2 μM BODIPY 493/503 and 10 μM Hoechst 33342) and 1% DMSO for 2 h in a CO_2 incubator. After washing with fresh medium to remove the free probes, the cells were kept in HBSS for imaging. The confocal images were recorded using a Leica TCS SP8 confocal microscopy with following set: λ_{ex} = 405 nm, λ_{em} = 415–465 nm for Hoechst 33342; λ_{ex} = 488 nm, λ_{em} = 500–550 nm for BODIPY493/503; λ_{ex} = 560 nm, λ_{em} = 650–750 nm for **Lipi-PS**.

General staining procedure of fluorescent probes. Live cells were stained in DMEM+ containing fluorescent probe (**Lipi-PS**, BODIPY493/503, or Nile Red) and 1% DMSO for 2 h in a CO_2 incubator. The probe concentration is 2 μM unless otherwise mentioned. Then, the cells were washed three times with fresh medium to remove the free probes, and kept in HBSS for imaging.

Confocal imaging. For the comparison of LDs labeling selectivity of fluorescent probes, the confocal images were recorded using a Leica TCS SP8 confocal microscopy with following

set: λ_{ex} = 488 nm, λ_{em} = 500–700 nm. For the comparison of the photostability of fluorescent probes, the confocal images were recorded using a Nikon A1 RMP confocal microscopy with an identical power of excitation laser (561 nm). The total signal intensities of each image were measured with ImageJ, normalized to the value of the first image and plotted as a function of the number of recorded confocal images.

Signal-to-noise ratio statistics. For quantifying the signal-to-noise ratios of three fluorescent probes, five frames of LDs fluorescence images were rocorded for each probe, and six sets of positions in each image were selected to calculate the signal-to-noise ratio by ImageJ software. Thus, for each probe, we obtained its average signal-to-noise ratio by statistically analyzing of 30 sets of data.

Image encoding. Spectral fluorescence images were recorded using a Leica TCS SP8 confocal microscopy or a Zeiss LSM 980 confocal microscopy. After the spectral image sequence information is read using Matlab software, each pixel is traversed to obtain the maximum wavelength peak position information through Gaussian fitting. Subsequently, pseudocolor assignment is carried out. The color mapping and the range of wavelength variation are defined first. Then, a RGB image is created and the intensity information is overlaid. The plotting results are displayed after gamma correction and brightness adjustment, with wavelength and polarity scales added.

HSFI of HeLa cells under various stimulation. Live HeLa cells were stained with 2 μM **Lipi-PS** for 2 h after various stimulation: cholesterol (conjugated to methyl β-cyclodextrin with a molar ratio of 1:12.5, 80 μM for 48 h), OA (400 μM for 12 h), starvation (12 h), chloroquine (20 μM for 12 h), erastin (20 μM for 12 h), RSL3 (20 μM for 12 h), CDDP (20 μM for 12 h). The cells were washed three times with fresh medium and then fixed with para-formaldehyde for 15 min. After that, the cells were washed three times with PBS and kept in PBS for imaging. The HSFI were carried out using a Zeiss LSM 980 confocal microscopy with the following set: λ_{ex} = 543 nm, λ_{em} = 592−752 nm, and a λ-step of 5 nm.

HSFI of various live cells. Live cells (HeLa, HepG2, HT22, and 7702) were stained with 2 μM **Lipi-PS** for 2 h. The cells were washed three times with fresh medium and kept in HBSS for imaging. The HSFI were carried out using a Leica TCS SP8 confocal microscopy with the following set: λ_{ex} = 550 nm, λ_{em} = 570−780 nm, and a λ-step of 10 nm.

Time-lapse 3D confocal imaging. The imaging experiments were conducted by a Leica TCS SP8 confocal microscopy with following set: λ_{ex} = 550 nm, λ_{em} = 570–780 nm, a scan speed of 200 Hz, a pixel resolution of 90.2 nm × 90.2 nm, a z-step of 300 nm, a total of 9 sets (interval of 30 min) of 3D imaging. The reconstruction of 3D confocal image was obtained

using a Leica LAS X software. In addition, a microscopy-suited incubator was employed to control the temperature (37 °C) and CO_2 concentration (5%).

Time-lapse HSFI. The imaging experiments were conducted by a Leica TCS SP8 confocal microscopy with following set: λ_{ex} = 550 nm, λ_{em} = 570–780 nm, a scan speed of 200 Hz, a pixel resolution of 90.2 nm × 90.2 nm, a λ-step of 10 nm, a total of 9 sets (interval of 30 min) of HSFI. In addition, a microscopy-suited incubator was employed to control the temperature (37 °C) and CO_2 concentration (5%).

Animal experiment. The six-week-old normal Balb/c male mice and NAFLD model Balb/c male mice used in the experiment were purchased from Liaoning Changsheng Biotechnology Co. Ltd. The wild type AB strain zebrafishes of different developmental stages (0 dpf, 1 dpf, 2 dpf, 3 dpf, and 7 dpf) were developed from the same batch of oosperm and provided by Changchun Weishi Testing Technology Service Co. Ltd. All animal experiments were performed according to the guidelines of the Institutional Animal Care and Use Committee. The Institutional Animal Ethics Committee of Jilin University approved the animal experiments (license no. SY202502001, SY202503002). Mice were killed by anesthesia followed by cervical dislocation and then the corresponding liver tissues were sampled. The zebrafish after the experiment were killed in an ice bath.

HSFI and two-photon 3D imaging of mouse liver tissue. After fresh liver tissues were taken out from normal mouse and NAFLD model mouse, cut them into slices and stained in DMEM+ containing 10 μM **Lipi-PS** for 4 h. After washed three times with PBS, the liver tissues slices were used for HSFI and two-photon 3D imaging. The HSFI were carried out using a Leica TCS SP8 confocal microscopy with the following set: λ_{ex} = 550 nm, λ_{em} = 570−780 nm, and a λ-step of 10 nm. For the two-photon 3D imaging of liver tissues, a Nikon A1 RMP two-photon microscopy was used with the following set: λ_{ex} = 980 nm, λ_{em} = 593–750 nm, and a *z*-step of 500 nm.

HSFI of zebrafish. The zebrafishes of various developmental stages (0 dpf, 1 dpf, 2 dpf, 3 dpf, and 7 dpf) were stained with 10 μM **Lipi-PS** for 4 h. The zebrafishes were washed three times with PBS and kept in PBS for HSFI. The HSFI were carried out using a Leica TCS SP8 confocal microscopy with the following set: λ_{ex} = 550 nm, λ_{em} = 570−780 nm, and a λ-step of 5 nm.

HSFI of *xyzλt* 5D-dynamic tracking of single LD. Live HeLa cells treated with cholesterol for 48 hours were stained with **Lipi-PS** for 2 hours and then stimulated with OA (400 μM) at the start of imaging. The imaging experiments were conducted by the home-made 3D-SpecDIM system[8] with the exciting laser of 561 nm, a pair of achromatic doublet lenses

and a pinhole was utilized for spatial filtering for the exciting laser. Then the laser polarization was tuned by a half waveplate (AHWP05M-580, Tholabs) and cleaned by Glan-Thompson polarizer (GLP10-A, Lbtek) before entering a pair of EODs (Model 310A, Conoptics Inc.). The laser beam was then expended by a pair of lenses (AC254-075-A-ML and AC254-300-A-ML, Thorlabs). A tunable acoustic gradient lens (TAGLENS-T1, Mitutoyo) was used to modulate the axial focus position. Then a pair of lenses (AC254-250-A-ML and AC254-150-A-ML, Thorlabs) were used to relay the laser to objective lens (HC PL APO 100x/1.40 OIL CS2, Leica). A dichroic mirror (ZT405/488/561/640 rpcv2, Chroma) was utilized for separating the excitation and emission light. Subsequently, emission fluorescence was filtered by band-pass filter and split by a beam splitter and directed into tracking and spectral imaging paths. In the tracking path, emission light was focused onto an APD using an achromatic lens (AC254-050-A-ML, Thorlabs). In the spectral path, the fluorescence was further divided by a 30/70 splitter (BSS10R, Thorlabs). A CaF2 prism (PS863, Thorlabs) was used to disperse the fluorescent spectral signal. Finally, the beams were reflected by right-angle prism mirror (MRA25L-E02, Thorlabs) and focused with two achromatic lenses (AC254-150-A-ML, Thorlabs). An EMCCD (iXon Ultra 897, Andor) was used to record the spectral distribution. A two-axis piezo nanopositioner (Nano-PDQ275, Mad City Lab) and a z axis piezo nanopositioner (Nano-OPQ65, Mad City Lab) were used to move the sample and objective, respectively. A FPGA (PCIe-7858, National Instruments Corp.) was utilized to count the fluorescent signal, calculate the molecular position, control the nanopositioners and record the 3D trajectory of molecules. The synchronization of 3D single molecule tracking and spectral imaging was realized by instant triggering EMCCD with tracking signal or by aligning the start time point of tracking and spectral signal.

Statistical analysis. All data were expressed as mean ± S.D., unless otherwise stated. The differences between groups were analyzed by two-way analysis of variance (ANOVA). Tukey's post hoc test was performed by data statistical analysis software (SPSS Statistics, Version number: 18.0.0.282). $p < 0.05$ was considered as statistically significant, expressing as one star, $p < 0.01$ as two stars, $p < 0.001$ as three stars and $p < 0.0001$ as four stars.

5. References of Supporting Information

[1] J. Dai, Z. Wu, D. Li, G. Peng, G. Liu, R. Zhou, C. Wang, X. Yan, F. Liu, P. Sun, J. Zhou, G. Lu, *Biosens. Bioelectron.* **2023**, *229*, 115243.

[2] H. M. Meshram, G. S. Kumar, P. Ramesh, B. C. Reddy, *Tetrahedron Lett.* **2010**, *51*, 2580–2585.

[3] J. Guo, J. Fan, X. Liu, Z. Zhao, B. Z. Tang, *Angew. Chem. Int. Ed.* **2020**, *59*, 8828–8832.

[4] G. Liu, G. Peng, J. Dai, R. Zhou, C. Wang, X. Yan, X. Jia, X. Liu, Y. Gao, L. Wang, G. Lu, *Anal. Chem.* **2021**, *93*, 14784–14791.

[5] T. Fiala, J. Wang, M. Dunn, P. Šebej, S. J. Choi, E. C. Nwadibia, E. Fialova, D. M. Martinez, C. E. Cheetham, K. J. Fogle, M. J. Palladino, Z. Freyberg, D. Sulzer, D. Sames, *J. Am. Chem. Soc.* **2020**, *142*, 9285−9301.

[6] K. Selvakumar, A. Zapf, A. Spannenberg, M. Beller, *Chem. Eur. J.* **2002**, *8*, 3901−3906.

[7] E. M. Santos, W. Sheng, R. E. Salmani, S. T. Nick, A. Ghanbarpour, H. Gholami, C. Vasileiou, J. H. Geiger, B. Borhan, *J. Am. Chem. Soc.* **2021**, *143*, 15091−15102.

[8] H. Sha, Y. Wu, Y. Zhang, X. Feng, H. Li, Z. Wang, X. Zhang, S. Hou, *BioRxiv preprint doi*: https://doi.org/10.1101/2024.09.25.614875.

6. Supporting Schemes, Figures and Tables

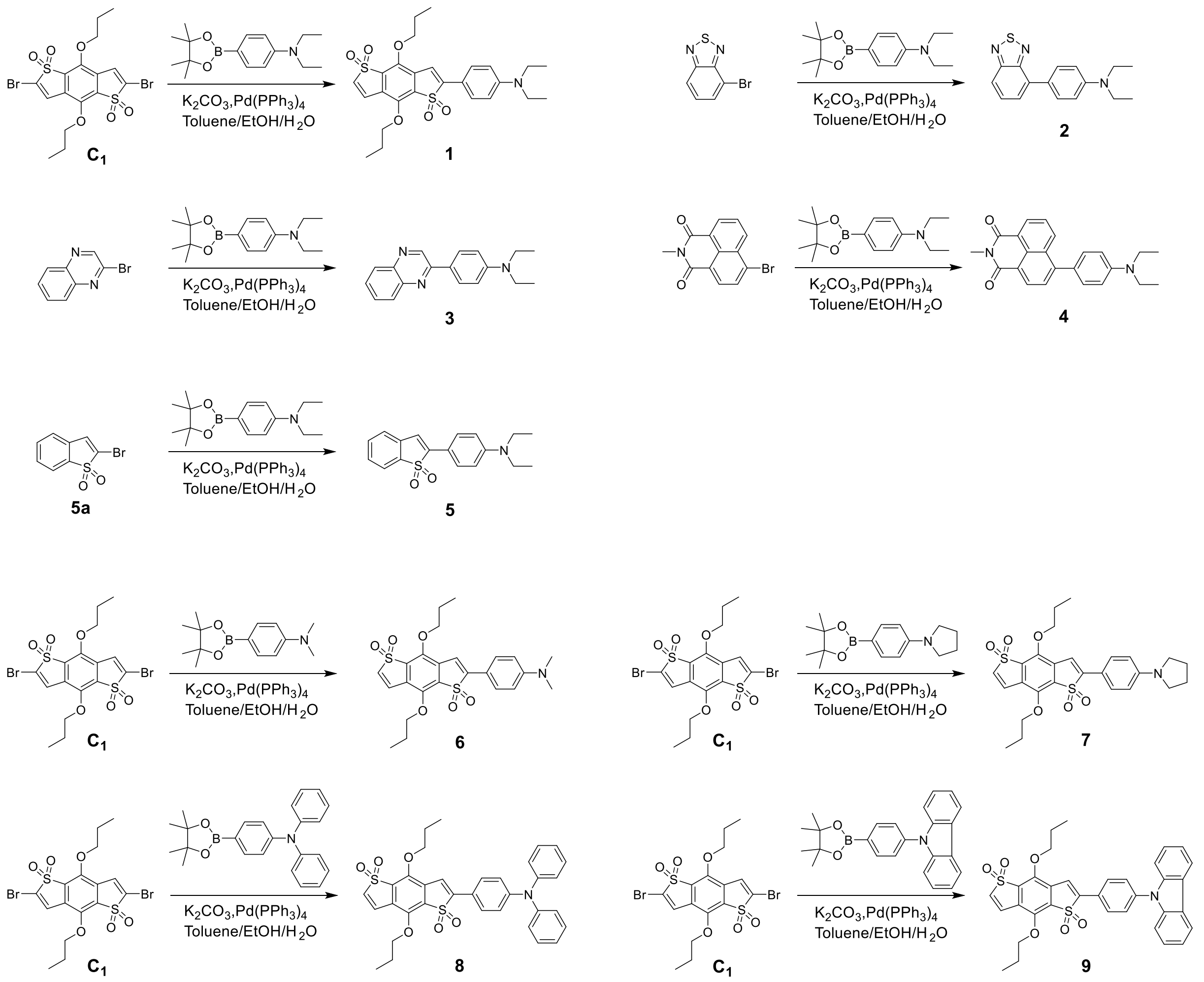

Scheme S1. Synthetic routes of molecules **1–9**.

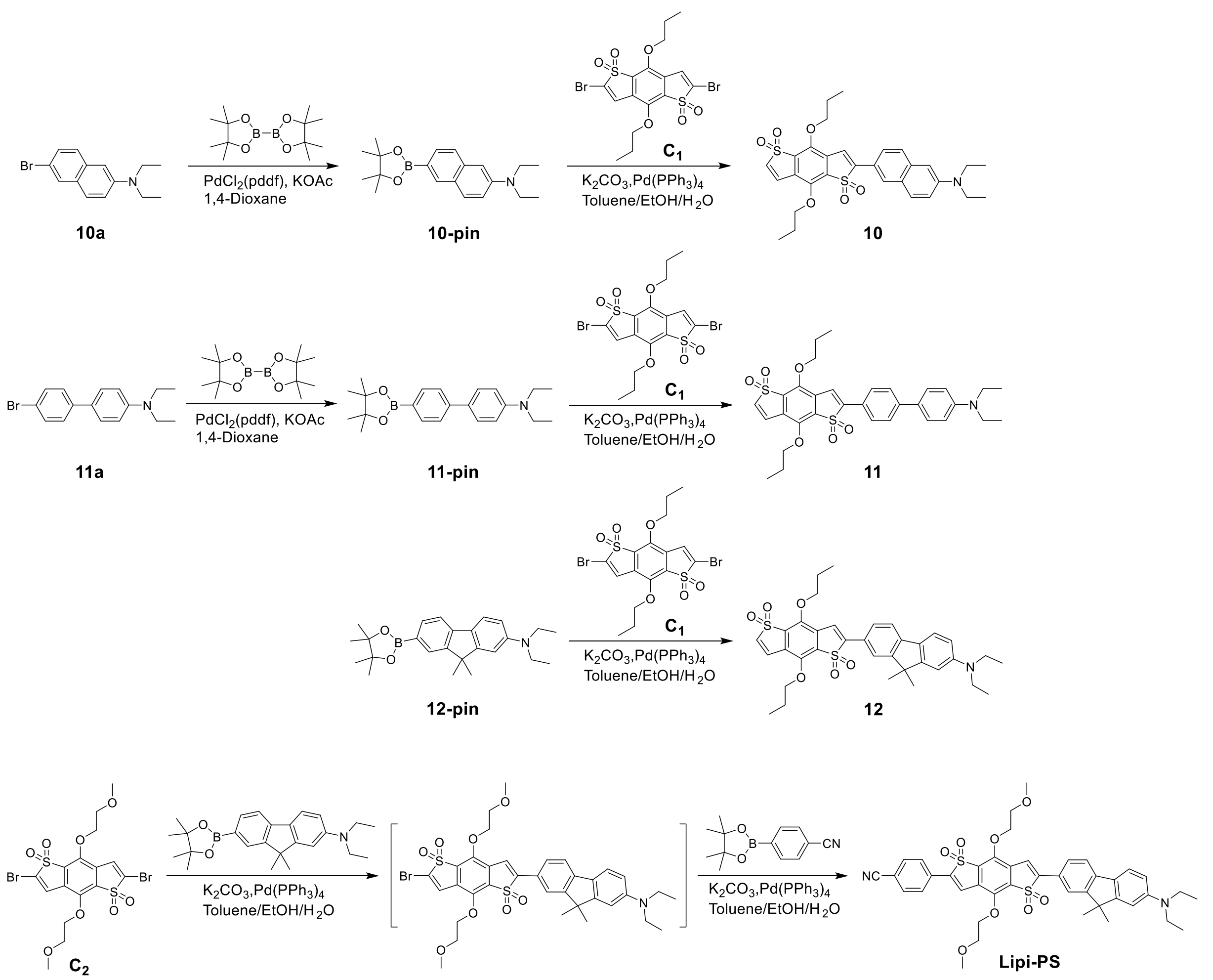

Scheme S2. Synthetic routes of molecules **10–12** and **Lipi-PS**.

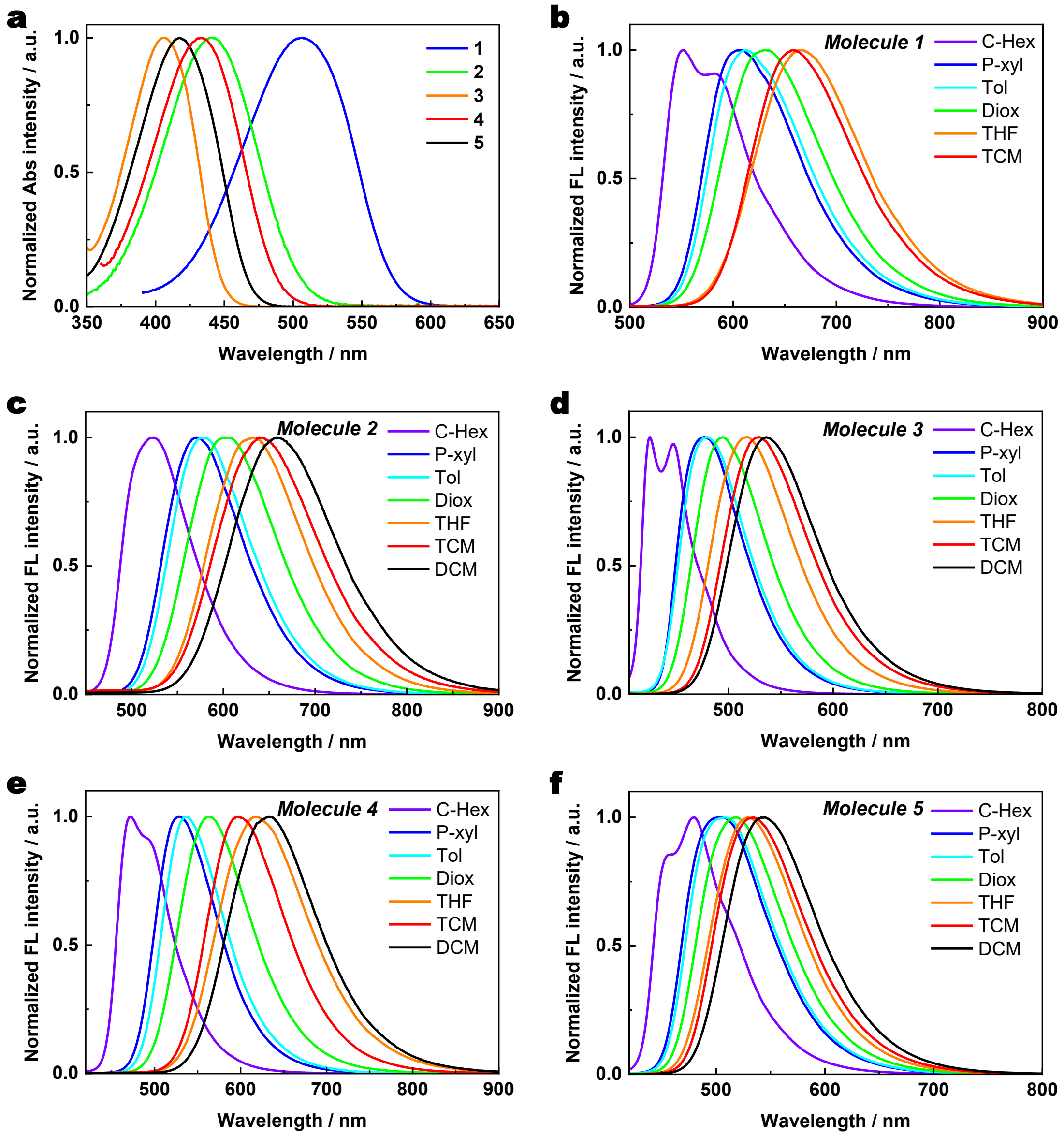

Figure S1. (a) Normalized absorption spectra of molecules **1**–**5** in toluene. (b–f) Normalized fluorescence spectra of molecules **1**–**5** in various organic solvents.

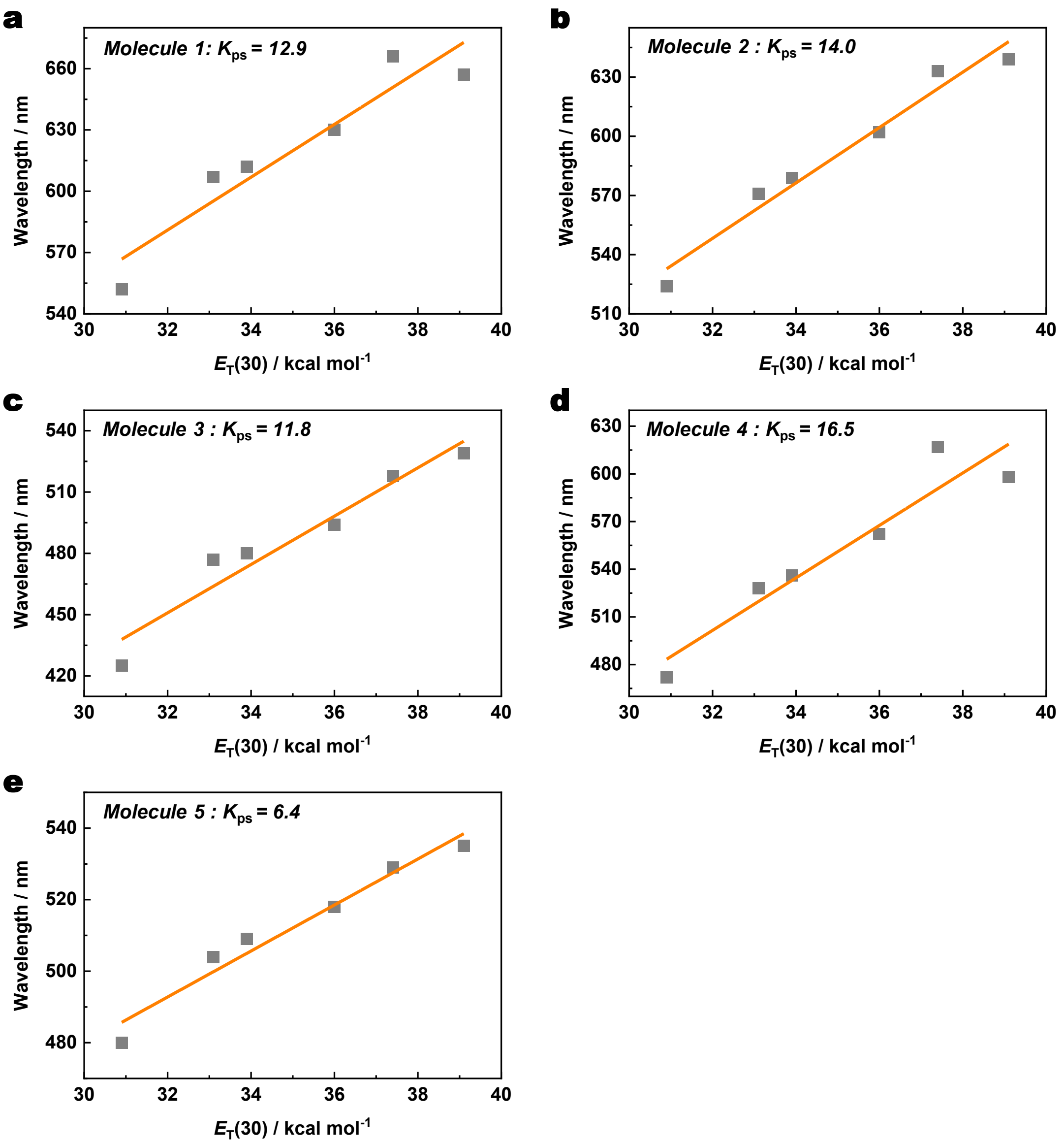

Figure S2. (a–e) The linear relationship between the fluorescence maximum wavelength and the solvent's polarity index $E_T(30)$ of molecules **1–5** in various organic solvents.

Table S1. Photophysical Data of Molecules **1–5** in Various Solutions

Molecule	λ_{abs} / nm	λ_{em} / nm							K_{ps}
	Toluene	C-hex	P-xyl	Tol	Diox	THF	$CHCl_3$	CH_2Cl_2	
**1**	506	552	607	612	630	666	657	681	**12.9**
**2**	440	524	571	579	620	633	639	658	**14.0**
**3**	407	425	477	480	494	518	529	536	**11.8**
**4**	433	472	528	536	562	617	598	632	**16.5**
**5**	418	480	504	509	518	529	535	545	**6.4**

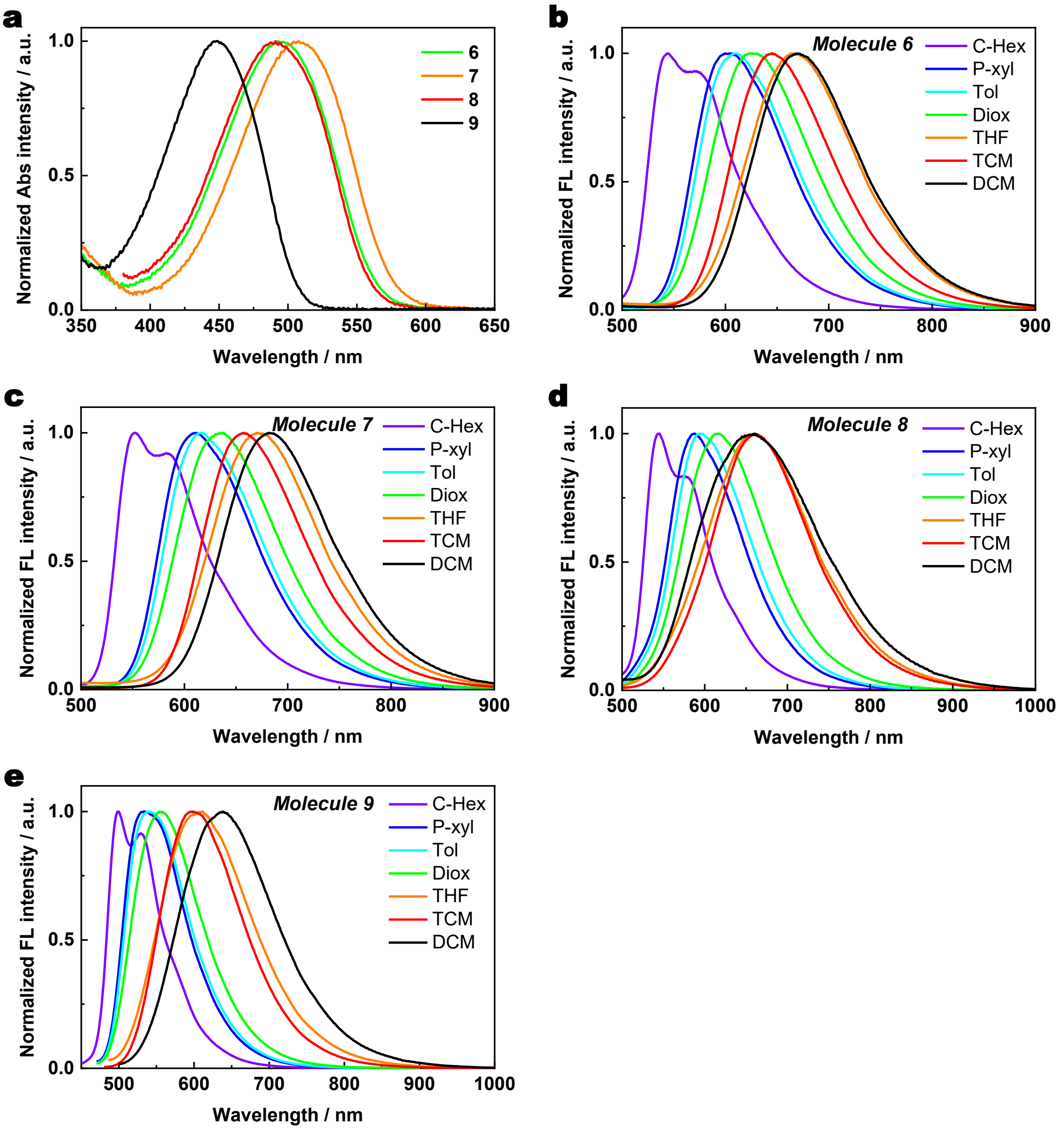

Figure S3. (a) Normalized absorption spectra of molecules **6**–**9** in toluene. (b–e) Normalized fluorescence spectra of molecules **6**–**9** in various organic solvents.

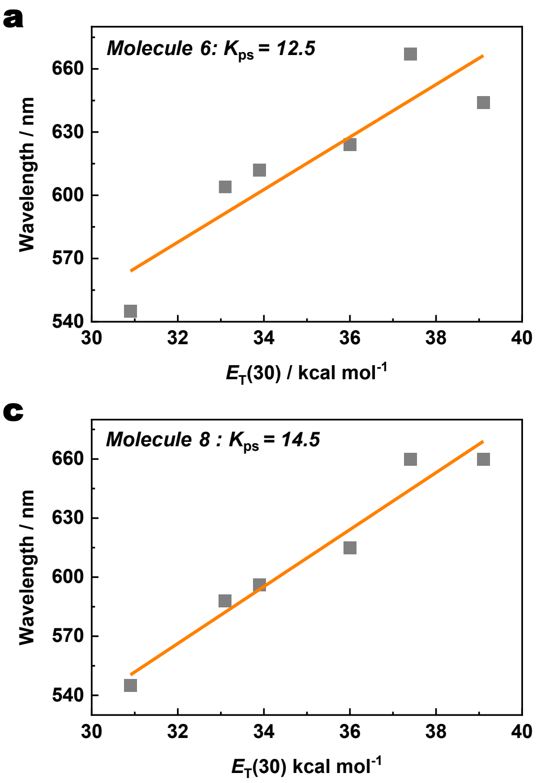

Figure S4. (a–d) The linear relationship between the fluorescence maximum wavelength and the solvent's polarity index $E_T(30)$ of molecules **6–9** in various organic solvents.

Table S2. Photophysical Data of Molecules **6–9** in Various Solutions

Molecule	λ_{abs} / nm	λ_{em} / nm							K_{ps}
	Toluene	C-hex	P-xyl	Tol	Diox	THF	$CHCl_3$	CH_2Cl_2	
**6**	493	545	604	612	624	667	644	670	**12.5**
**7**	508	552	612	615	637	670	658	682	**13.1**
**8**	491	545	588	596	615	660	660	660	**14.5**
**9**	449	500	533	540	554	611	599	638	**13.2**

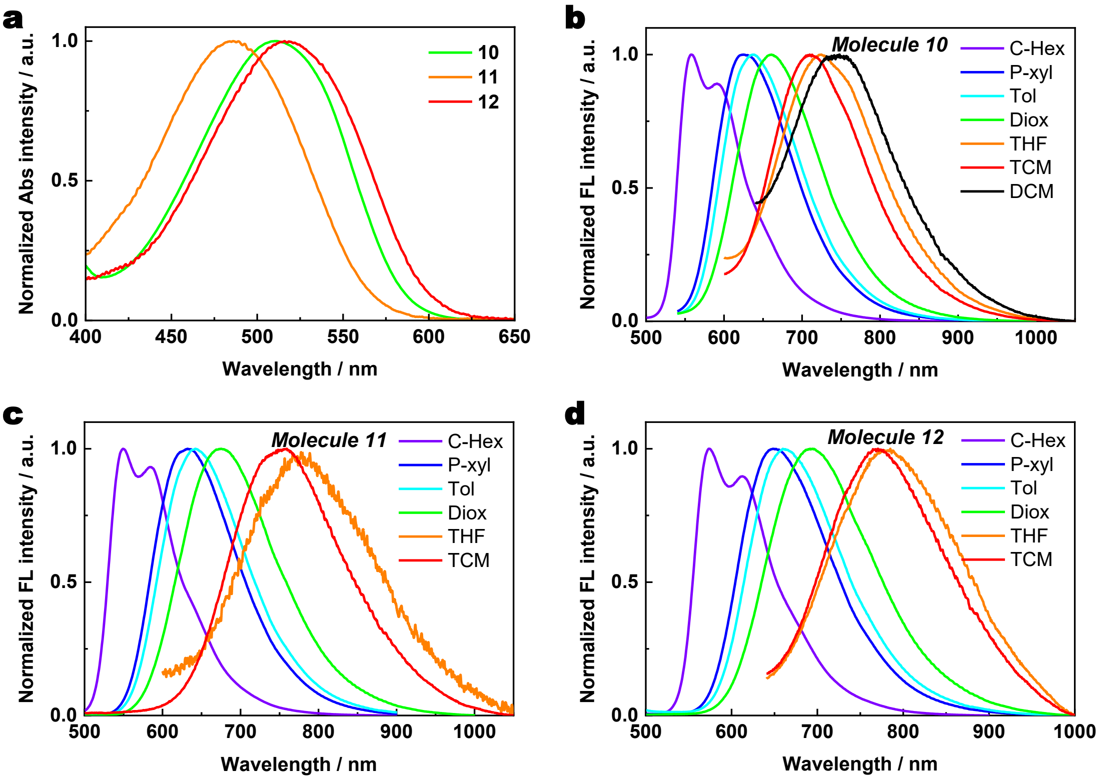

Figure S5. (a) Normalized absorption spectra of molecules **10–12** in toluene. (b–d) Normalized fluorescence spectra of molecules **10–12** in various organic solvents.

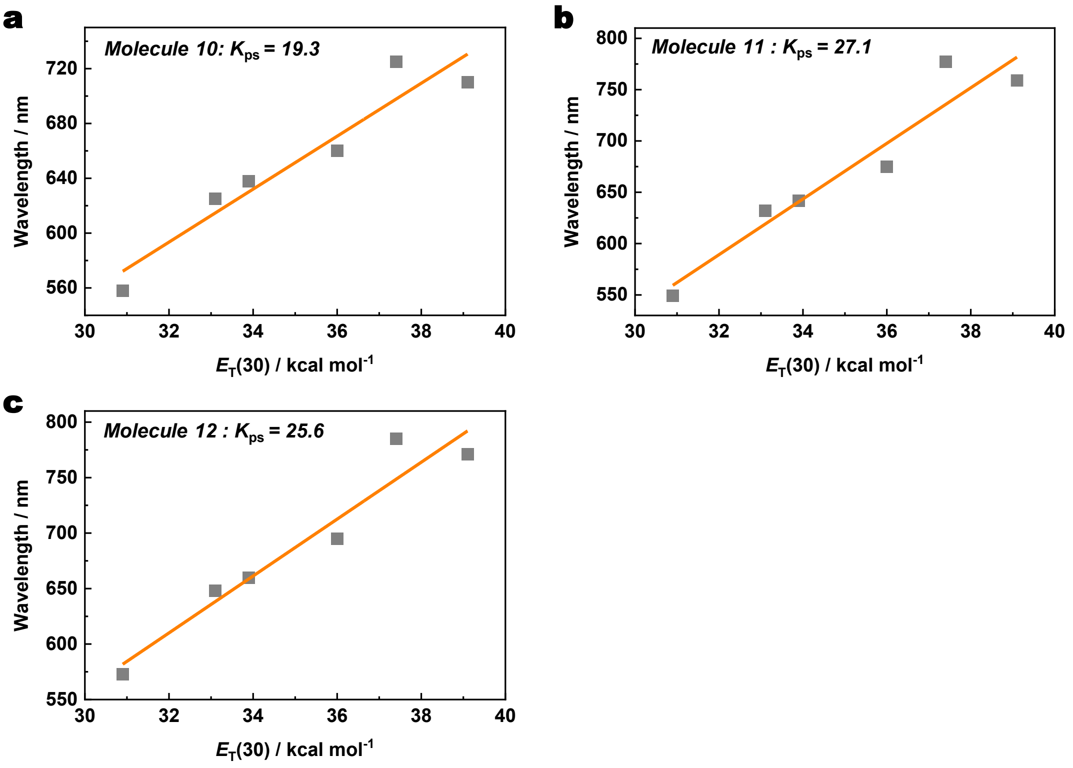

Figure S6. (a–c) The linear relationship between the fluorescence maximum wavelength and the solvent's polarity index $E_T(30)$ of molecules **10–12** in various organic solvents.

Table S3. Photophysical Data of Molecules **1**, **10**, **11** and **12** in Various Solutions

Molecule	λ_{abs} / nm	λ_{em} / nm, Φ_F [a]							K_{ps}
	Toluene	C-hex	P-xyl	Tol	Diox	THF	$CHCl_3$	CH_2Cl_2	
**1**	506	552, 91%	607, 89%	612, 99%	630, 91%	666, 68%	657, 78%	681, 61%	**12.9**
**10**	511	558 84%	625, 90%	638, 87%	660, 66%	725, 13%	710, 24%	748, 8%	**19.3**
**11**	486	549, 41%	632, 84%	642, 78%	675, 38%	777, 1%	759, 4%	–	**27.1**
**12**	516	573, 97%	648, 76%	660, 71%	695, 35%	785, 1%	771, 4%	–	**25.6**

[a] Absolute fluorescence quantum yield determined by a calibrated integrating sphere system.

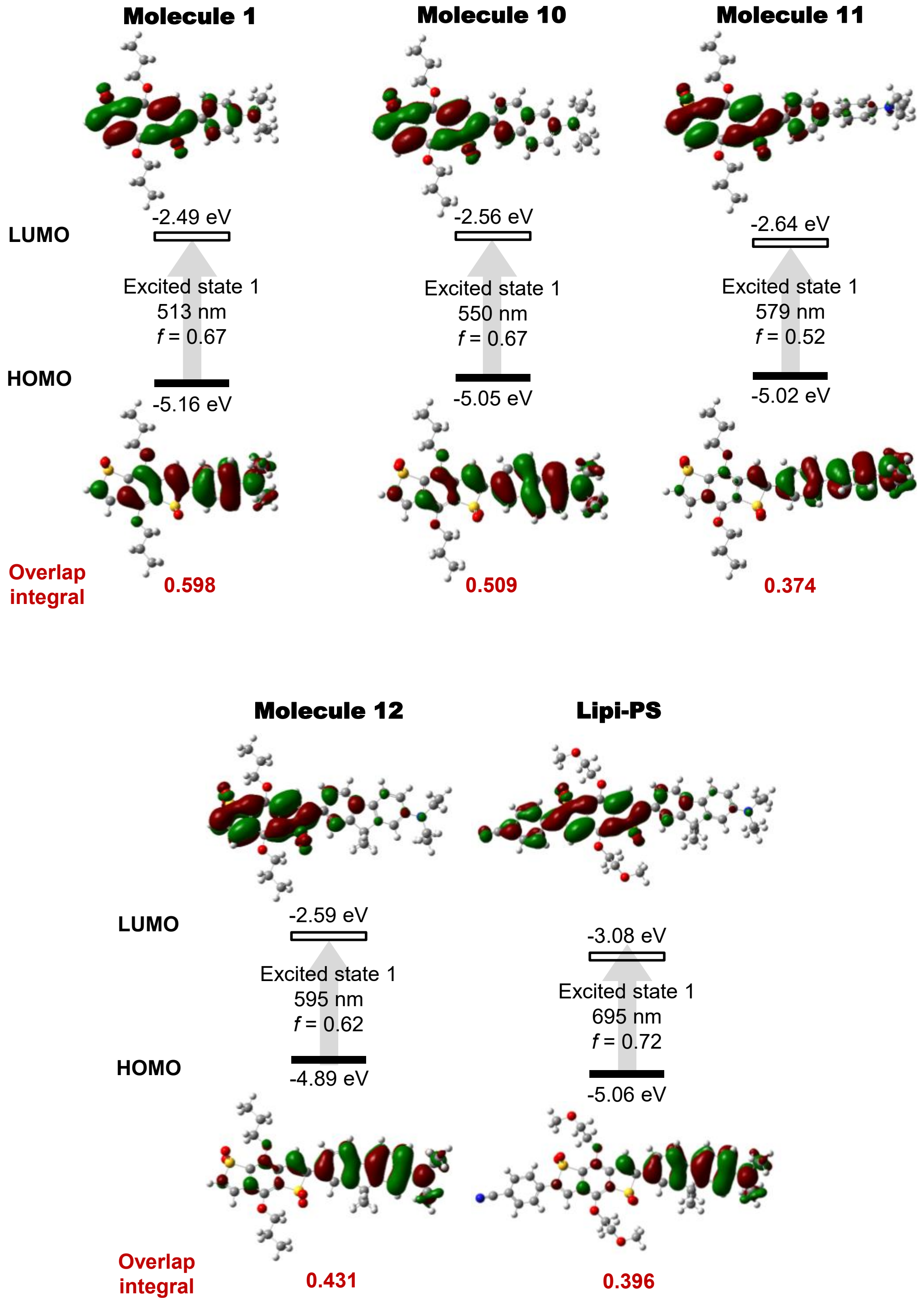

Figure S7. TD-DFT calculation (B3LYP/6-31G*, Gaussian 16) results of molecules **1**, **10**, **11** **12** and **Lipi-PS**: energy diagram, Kohn–Sham HOMO and LUMO, vertical excitation energy, oscillator strength (*f*) and orbital overlap integral between HOMO and LUMO. The first excited state corresponds to the transition of HOMO to LUMO.

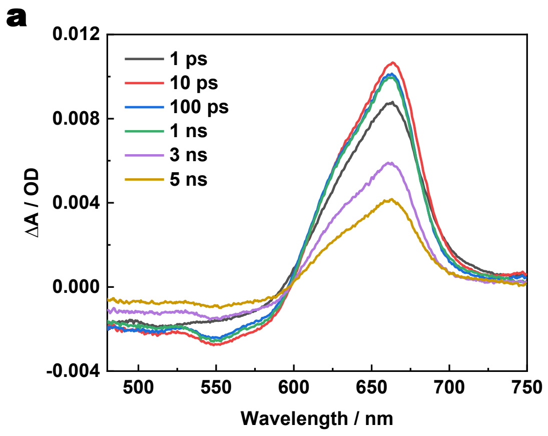

Figure S8. Fs-TA study of molecule **1** in c-hexane: (a) Fs-TA spectra as a function of time delay; (b) the evolution-associated spectra obtained from global fitting analysis.

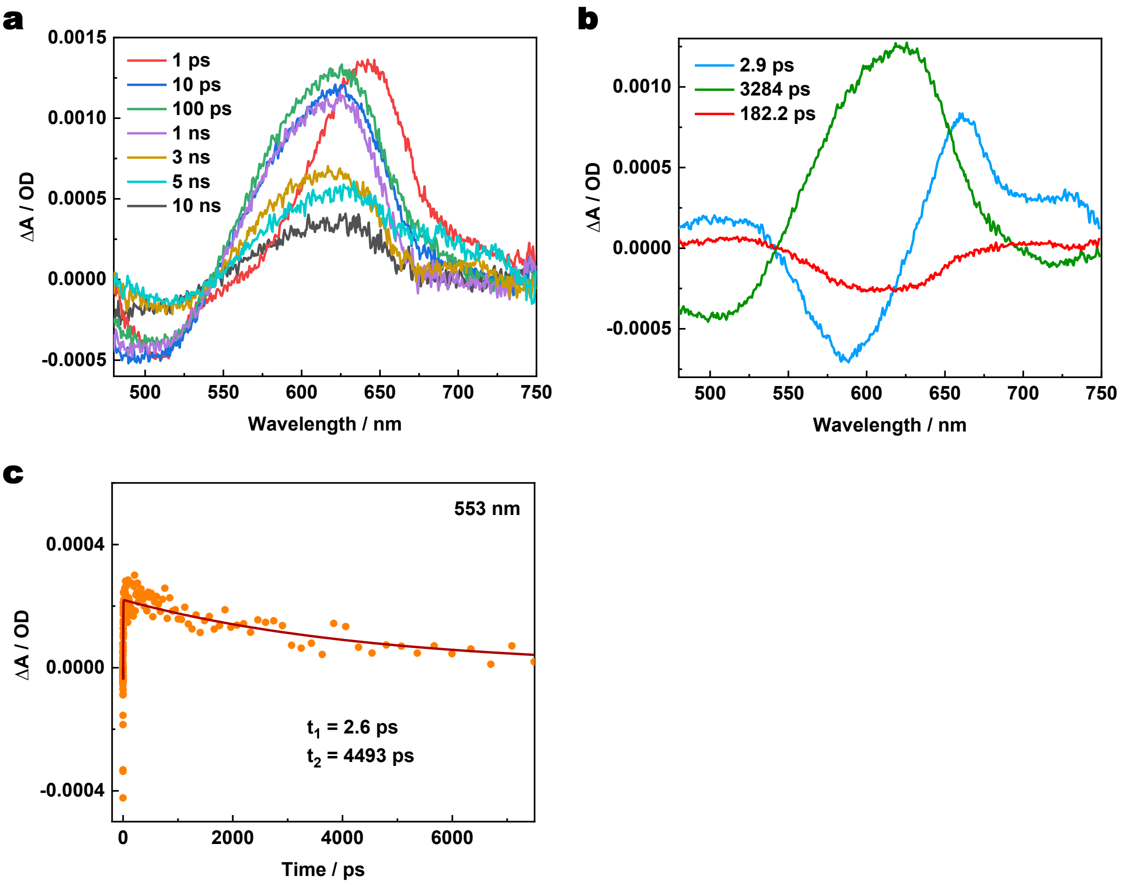

Figure S9. Fs-TA study of molecule **1** in dioxane: (a) Fs-TA spectra as a function of time delay; (b) the evolution-associated spectra obtained from global fitting analysis; (c) fitting kinetics of the corresponding band.

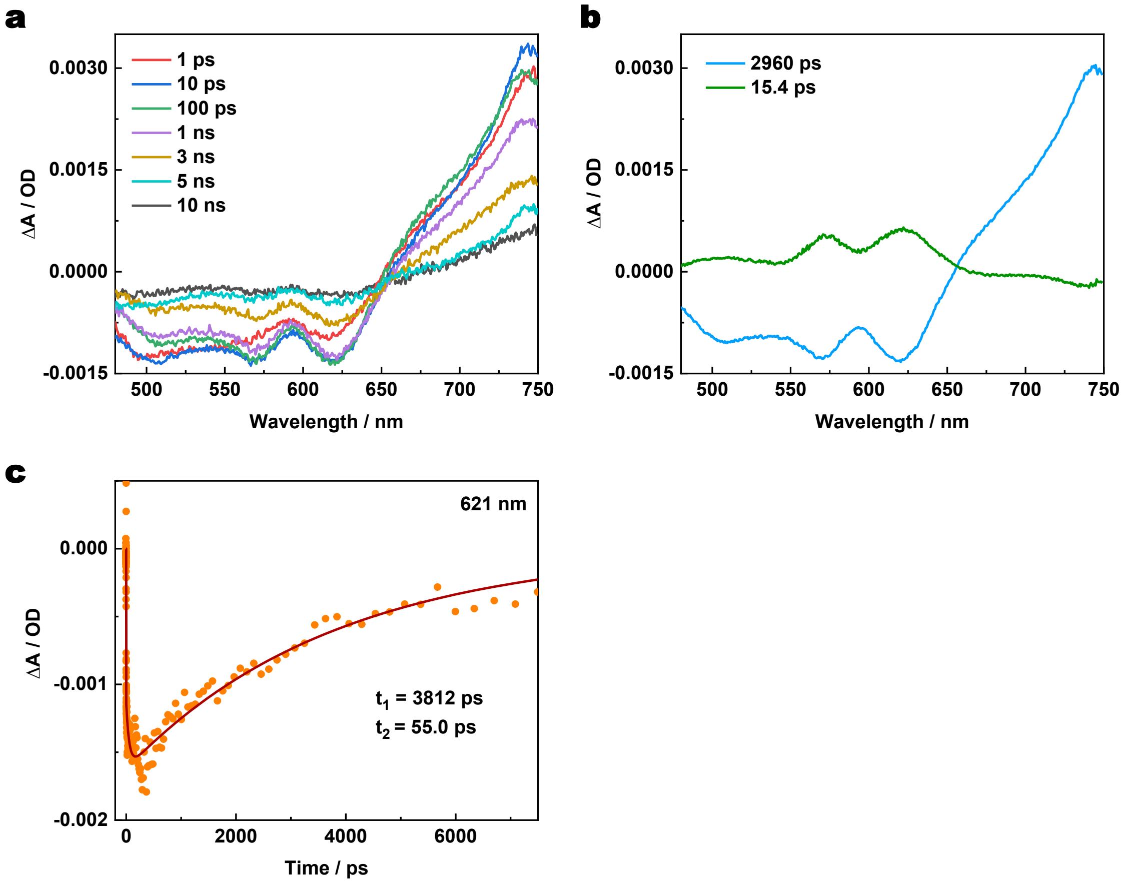

Figure S10. Fs-TA study of molecule **12** in c-hexane: (a) Fs-TA spectra as a function of time delay; (b) the evolution-associated spectra obtained from global fitting analysis; (c) fitting kinetics of the corresponding band.

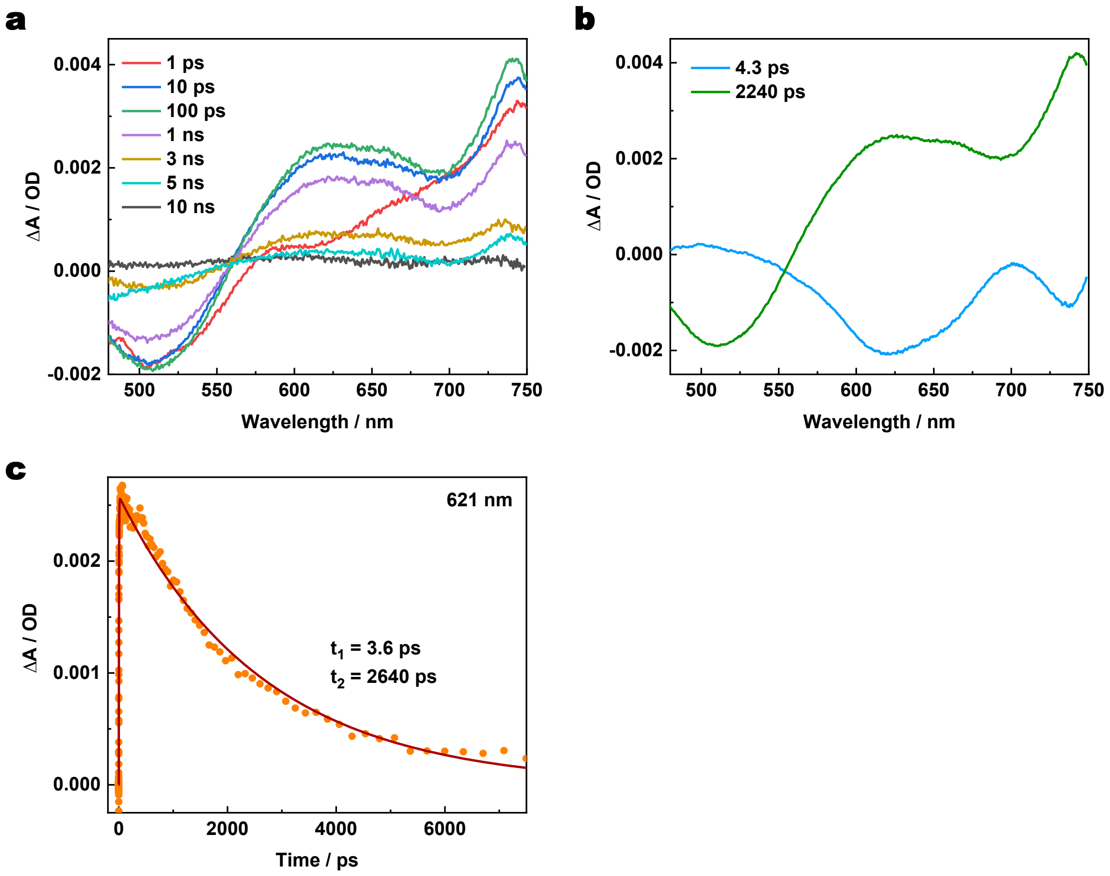

Figure S11. Fs-TA study of molecule **12** in dioxane: (a) Fs-TA spectra as a function of time delay; (b) the evolution-associated spectra obtained from global fitting analysis; (c) fitting kinetics of the corresponding band.

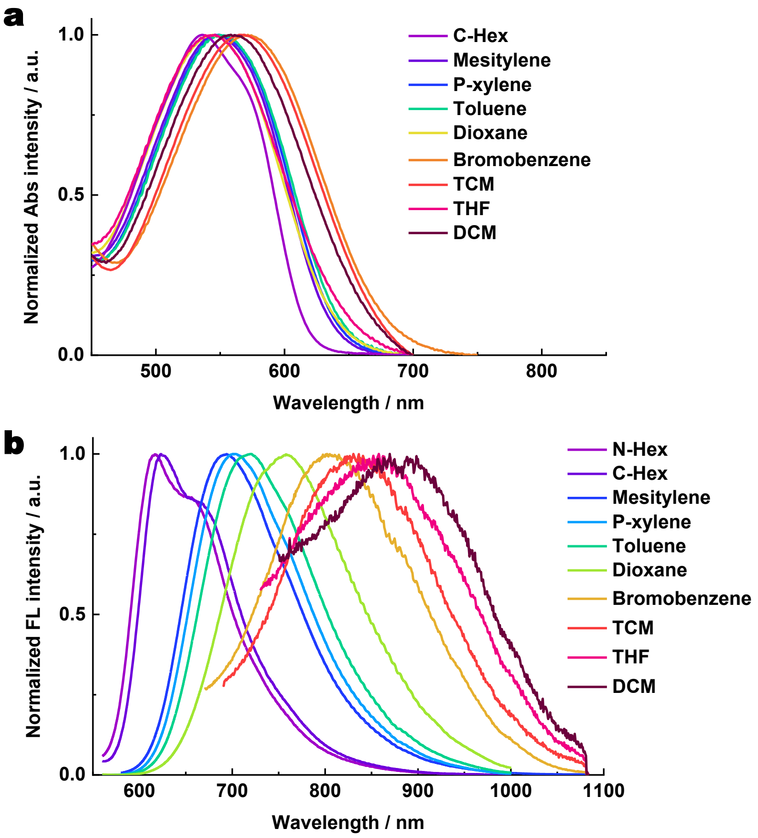

Figure S12. (a–b) Normalized absorption and fluorescence spectra of **Lipi-PS** in various organic solvents.

Table S4. Photophysical Data of **Lipi-PS** in Various Organic Solvents

Solvent	$E_T(30)$ / kcal mol^{-1}	λ_{abs} / nm	λ_{em} / nm	$\Phi_F{}^a$ / %
N-Hexane	31.0	538	617	39
C-Hexane	30.9	534	623	49
Mesi-tylene	32.9	547	694	49
P-xylene	33.1	549	703	38
Toluene	33.9	549	720	30
Dioxane	36.0	544	758	8
Bromobenzene	36.6	572	802	3
THF	37.4	546	858	--
$CHCl_3$	39.1	566	836	--
CH_2Cl_2	40.7	558	888	--

[a] Absolute fluorescence quantum yield determined by a calibrated integrating sphere system.

Table S5. The Polarity Slope K_{ps} of Representative Polarity-Sensitive Fluorescent Probes

Probe	Solvent-Fluorescence maximium wavelength / nm	K_{ps}	Reference
Nile Red	Tol-571, Dio-583, EA-595, THF-595, DCM-601, DMF-618, DMSO-629	**4.9**	*J. Am. Chem. Soc.* **2010**, *132,* 3169−3179.
Prodan	N-Hex-389, Tol-416, Dio-422, THF-430, DCM-440, MeCN-455, DMSO-462	**4.2**	*J. Am. Chem. Soc.* **2012**, *134,* 12418−12421.
FR0	Tol-434, Dio-445, THF-462, DCM-497, MeCN-518, DMF-512	**7.9**	*J. Phys. Chem. Lett.* **2010**, *1*, 616–620.
1	Tol-528, TCM-553, DCM-565, Acet-575, Ethanol-593, DMSO-601	**3.9**	*Angew. Chem. Int. Ed.* **2015,** *54*, 4539−4543.
DAF	Tol-439, EA-465, TCM-491, Ace-493, MeCN-511, DMSO-520	**6.4**	*Anal. Chem.* **2019**, *91*, 1928−1935.
PS-CDs	Dio-549, DMSO-581, H_2O-609	**2.4**	*Light: Sci. & Appl.* **2022**, *11*, 185.
LDs-Red	Tol-631, Dio-642, THF-659, TCM-673, DCM-680, MeCN-717	**7.5**	*Anal. Chem.* **2022**, *94*, 12095−12102.
Lipi-DSB	Tol-540, Dio-551, EA-583, TCM-564, DCM-588, MeCN-636	**8.1**	*ACS Materials Lett.* **2021**, *3*, 516−524.
Lipi-DSBOMe	Tol-566, Dio-570, EA-586, TCM-589, DCM-604, MeCN-641	**6.7**	*Sens. & Actuators: B. Chem.* **2023**, *387*, 133772.
Lipi-Deep Red	P-xyl-638, Tol-644, Dio-664, THF-729, TCM-719, DCM-761, MeCN-774	**12.0**	*Biosens. and Bioelectron.* **2023**, *229*, 115243.
NCIC-Pola	Tol-670, Dio-692, TCM-704, THF-717, DCM-721, MeCN-757, DMF-791	**8.8**	*Biosens. and Bioelectron.* **2023**, *237*, 115453.
LIP-Ser	Tol-637,Dio-634,TCM-684,THF-670,DCM-705,DMSO-731,DMF-706	**8.8**	*Biosens. and Bioelectron.* **2023**, *231*, 115289.
DCIQ	Tol-679, Dio-712, EA-752, THF-765, DCM-775, DMSO-811, MeCN-806	**10.1**	*Biosens. and Bioelectron.* **2023**, *240*, 115646.
TBPCPP	Tol-580, Dio-594, EA-614, DCM-630, DMSO-667, DMF-657	**8.0**	*Anal. Chem.* **2023**, *95*, 15350–15356.
Bu_2N-TTz-Py	N-Hex-450, TCM-532, DCM-515, MeCN-557, MeOH-565	**4.6**	*J. Am. Chem. Soc.* **2019**, *141,* 18780−18790.

DXB-NIR	C-Hex-622, Tol-687, Dio-699, EA-720, Acet-751, MeCN-760, DMF-769, DMSO-778	**9.1**	*J. Phys. Chem. Lett.* **2019**, *10*, 2414−2421.
7AMC	Heptane-388, Tol-432, Dio-451, THF-470, DCM-488, DMSO-545, MeCN-530	**9.8**	*J. Phys. Chem. Lett.* **2012**, *3*, 1011−1016.
**Lipi-PS**	N-Hex-617, C-Hex-623, Mesi-694, P-xyl-703, Tol-720, Dio-758, Bromobenzene-802	**29.8**	**This work**

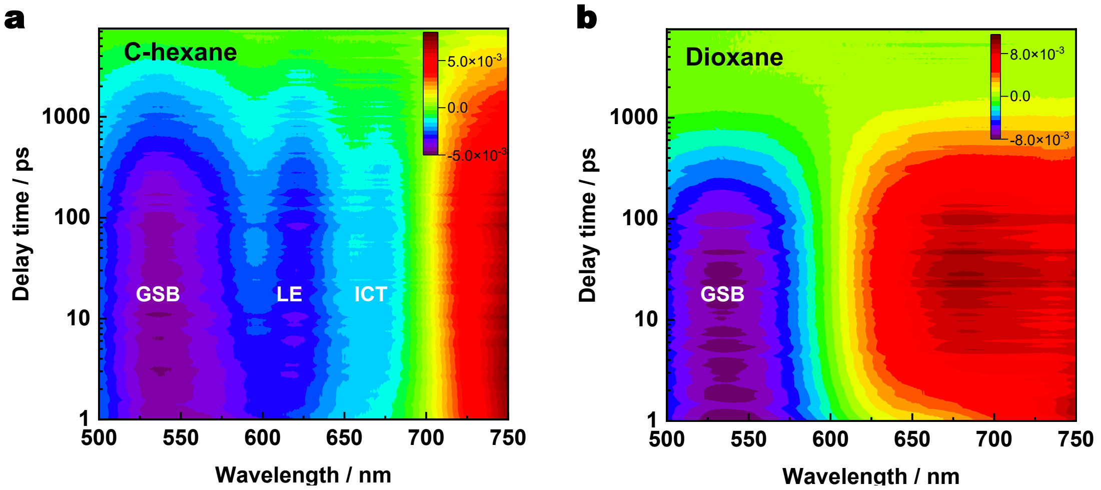

Figure S13. (a–b) Fs-TA spectra of **Lipi-PS** in C-hexane and dioxane.

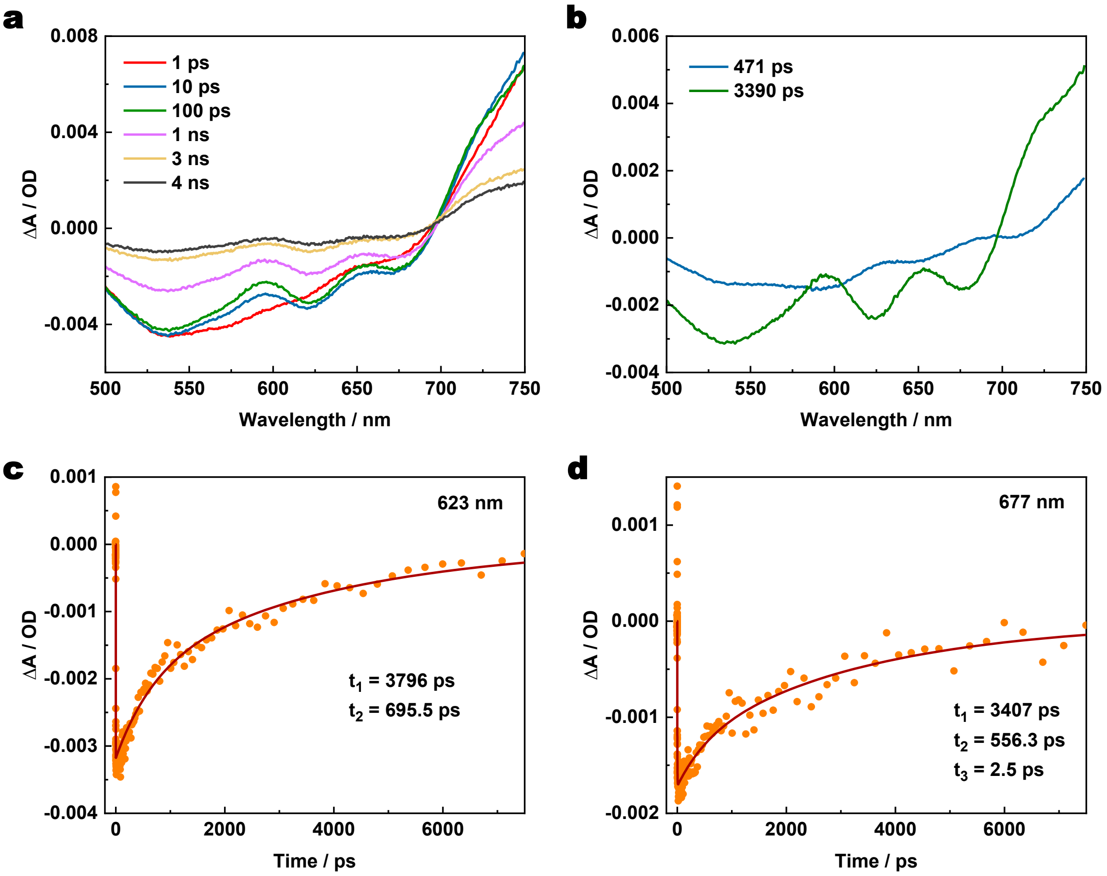

Figure S14. Fs-TA study of **Lipi-PS** in C-hexane: (a) Fs-TA spectra as a function of time delay; (b) the evolution-associated spectra obtained from global fitting analysis; (c–d) fitting kinetics of the corresponding bands.

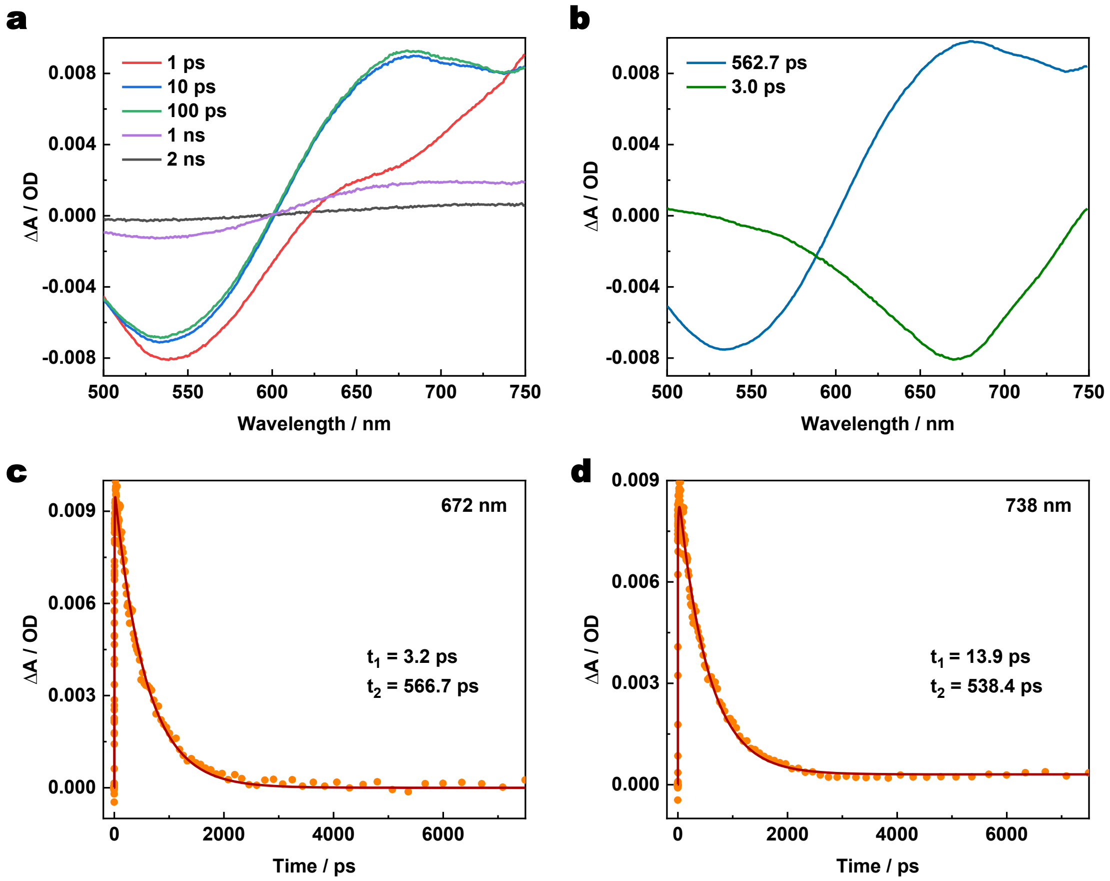

Figure S15. Fs-TA study of **Lipi-PS** in dioxane: (a) Fs-TA spectra as a function of time delay; (b) the evolution-associated spectra obtained from global fitting analysis; (c–d) fitting kinetics of the corresponding bands.

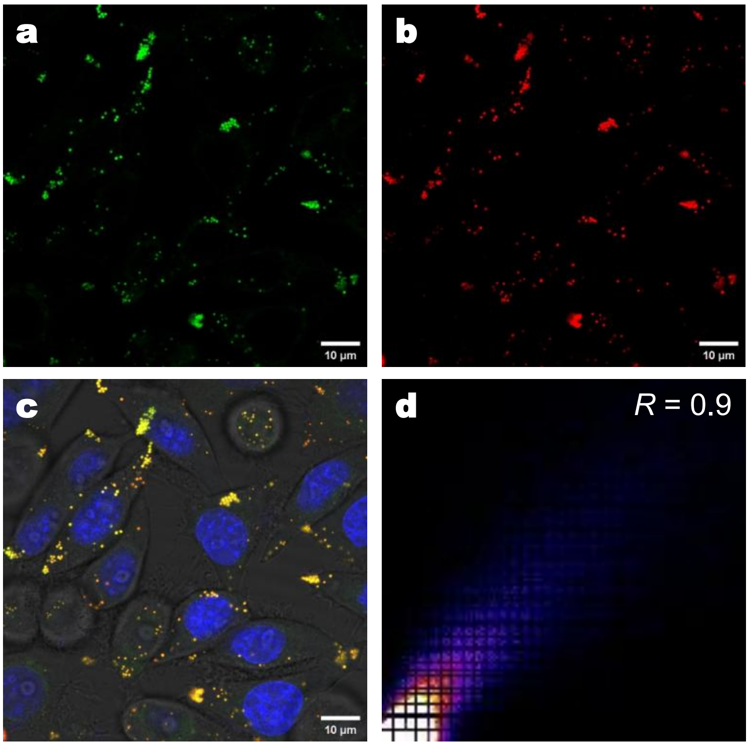

Figure S16. Colocalization confocal imaging of HeLa cells stained with **Lipi-PS**, BODIPY 493/503 and Hoechst 33342: (a) the green imaging channel of BODIPY 493/503 (λ_{ex} = 488 nm, λ_{em} = 500–550 nm); (b) the red imaging channel of **Lipi-PS** (λ_{ex} = 560 nm, λ_{em} = 650–750 nm); (c) the merged image of three fluorescence channels (the blue imaging channel is Hoechst 33342, λ_{ex} = 405 nm, λ_{em} = 415–465 nm) and bright filed; (d) the Pearson's correlation coefficient plot of the green and red fluorescence channels. Scale bar: 10 µm.

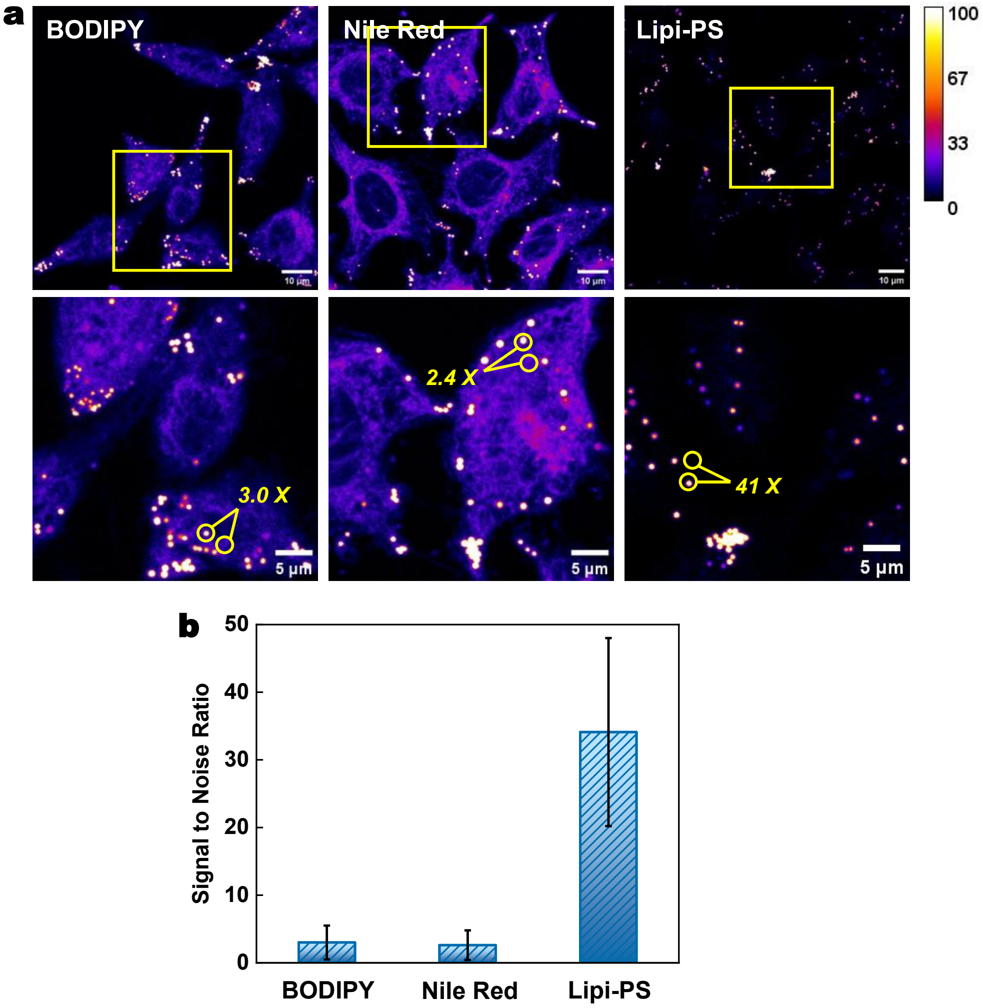

Figure S17. Comparison of the LDs staining specificity between **Lipi-PS** and the representative LDs probe BODIPY 493/503 and Nile Red. (a) The confocal images of living HeLa cells stained with different fluorescent probes (2 μM, 2 h) and the enlarged views of ROIs (marked with yellow squares). Scale bar: 10 μm for images; 5 μm for enlarged views. (b) The statistic results of signal-to-noise ratios of different fluorescent probes.

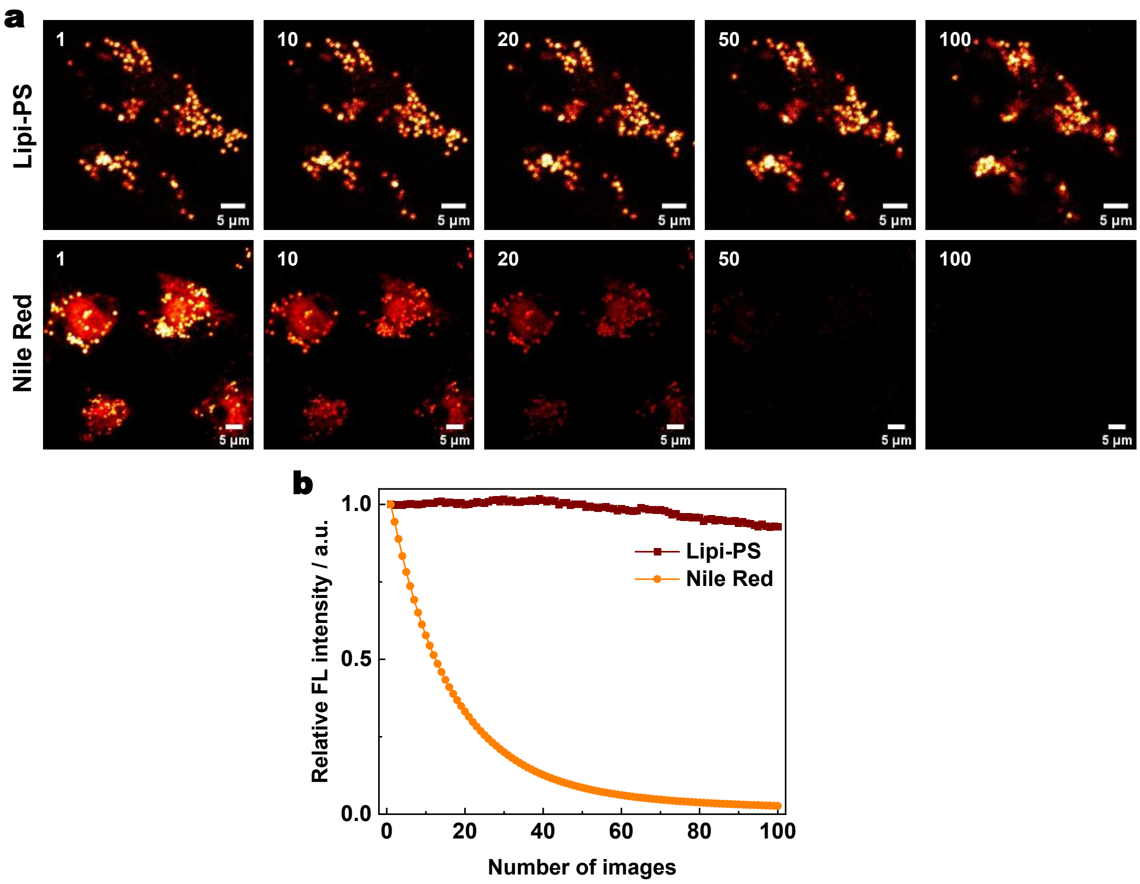

Figure S18. Comparison of the photostability between **Lipi-PS** and Nile Red. (a) The confocal images of HeLa cells stained with the two probes were repeatedly recorded under the identical intense excitation condition. The confocal images of number 1, 10, 20, 50 and 100 were shown. Scale bar: 5 μm. (b) The relative fluorescence intensity of each image was plotted as a function of the image number.

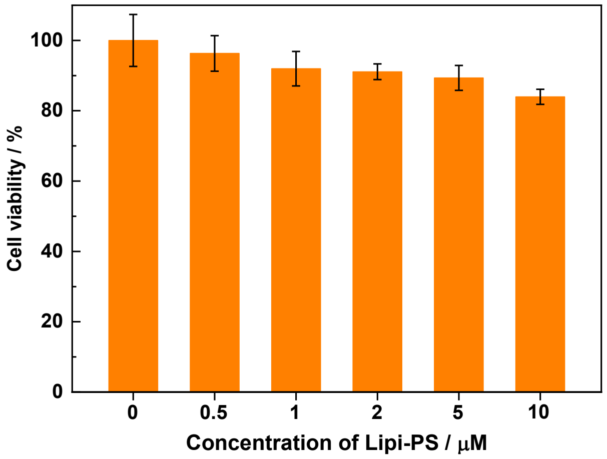

Figure S19. Cell viability results of the HeLa cells stained with **Lipi-PS** by MTT assay. The results are expressed as percentages of the probe-free controls (n = 10).

Table S6. Fluorescence Maxima of **Lipi-PS** in Various Organic Solvents Measured by Spectrometer and Microscopy

Solvent	N-Hexane	C-Hexane	Mesitylene	P-xylene	Toluene	Dioxane
$E_T(30)$ / kcal mol^{-1}	31.0	30.9	32.9	33.1	33.9	36.0
λ_{em} / nm (Spectrometer)	617	623	694	703	720	758
λ_{em} / nm (Leica TCS SP8 Microscopy)	614	619	678	685	696	723
λ_{em} / nm (Zeiss LSM 980 Microscopy)	--	624	677	680	688	696

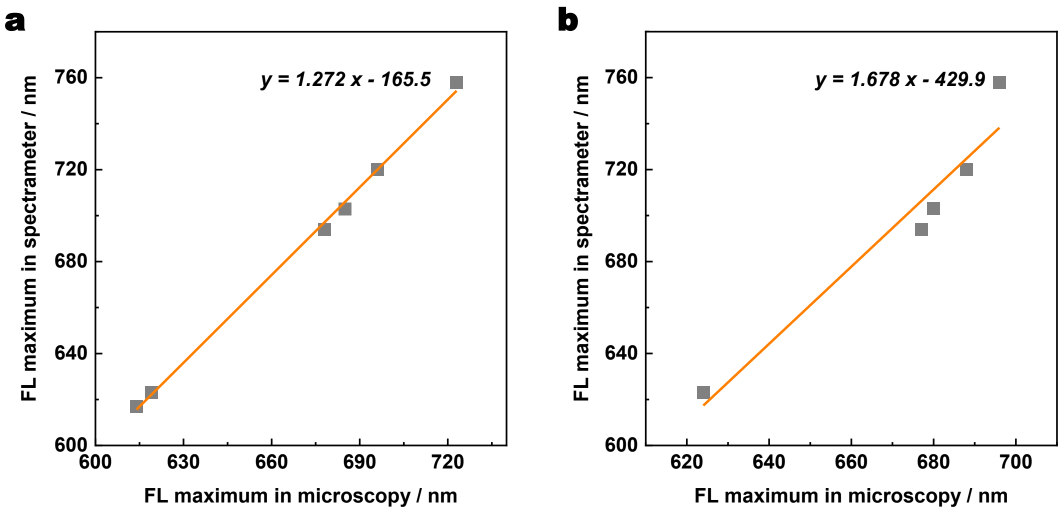

Figure S20. Linear fitting between the fluorescence maxima obtained from spectrometer and various microscopies: (a) Leica SP8 confocal microscopy; (b) Zeiss LSM 980 confocal microscopy.

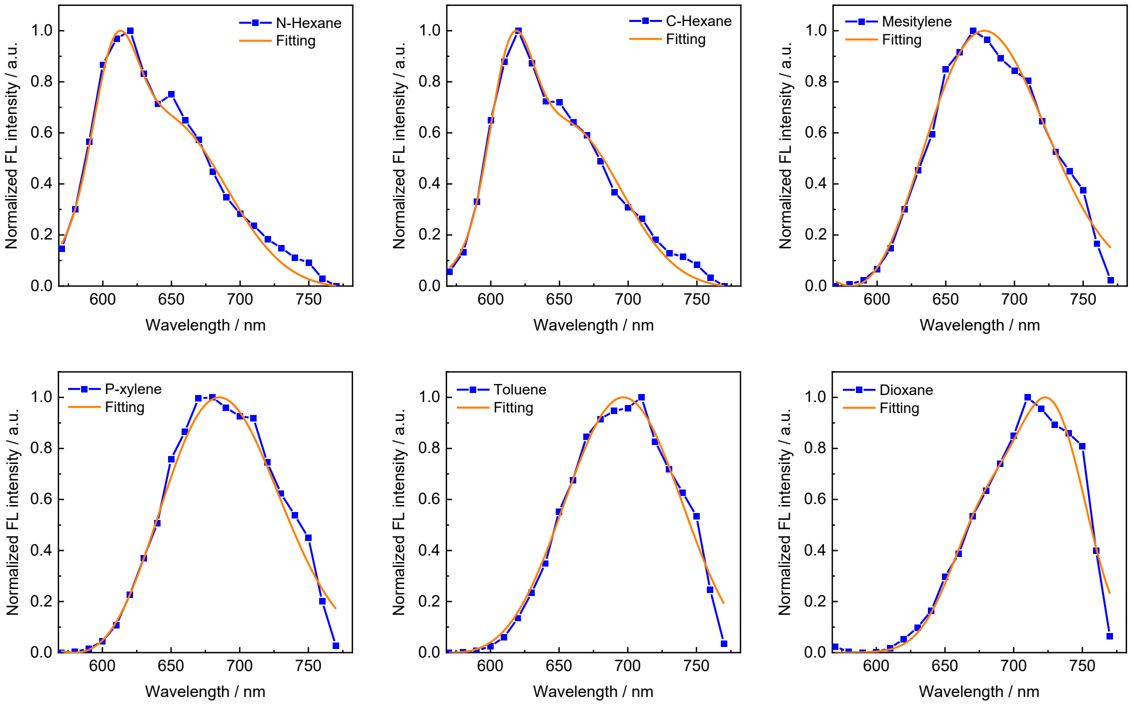

Figure S21. Normalized fluorescence spectra of **Lipi-PS** in various organic solvents measured by Leica SP8 confocal microscopy.

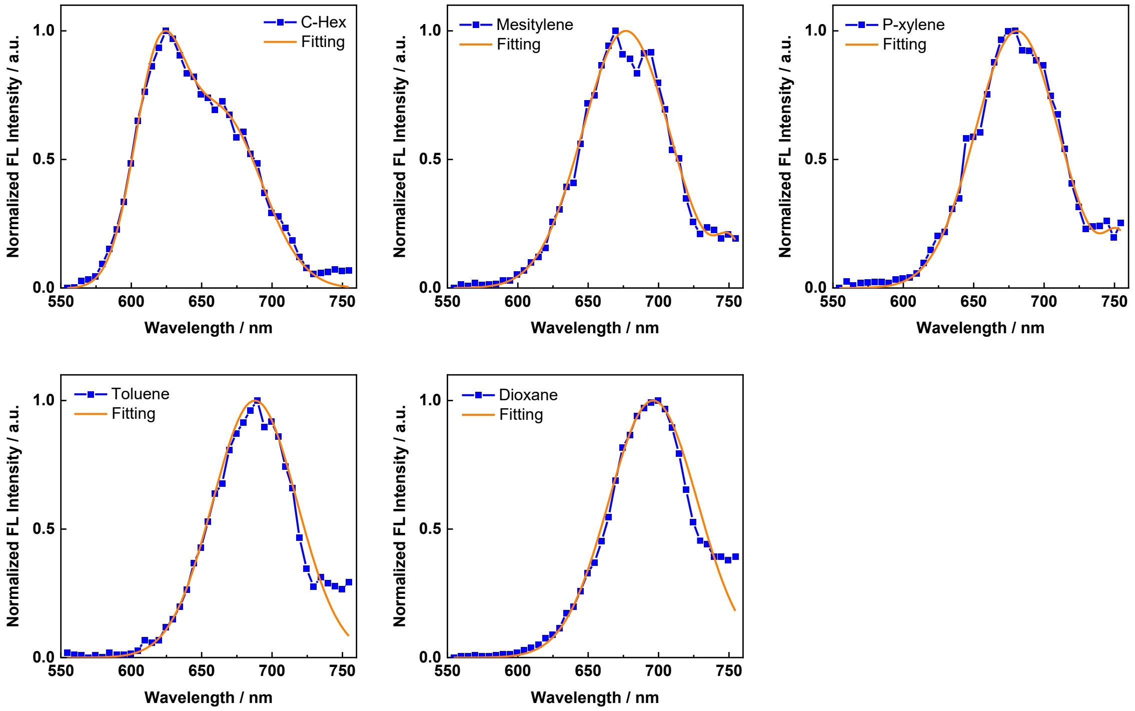

Figure S22. Normalized fluorescence spectra of **Lipi-PS** in various organic solvents measured by Zeiss LSM 980 confocal microscopy.

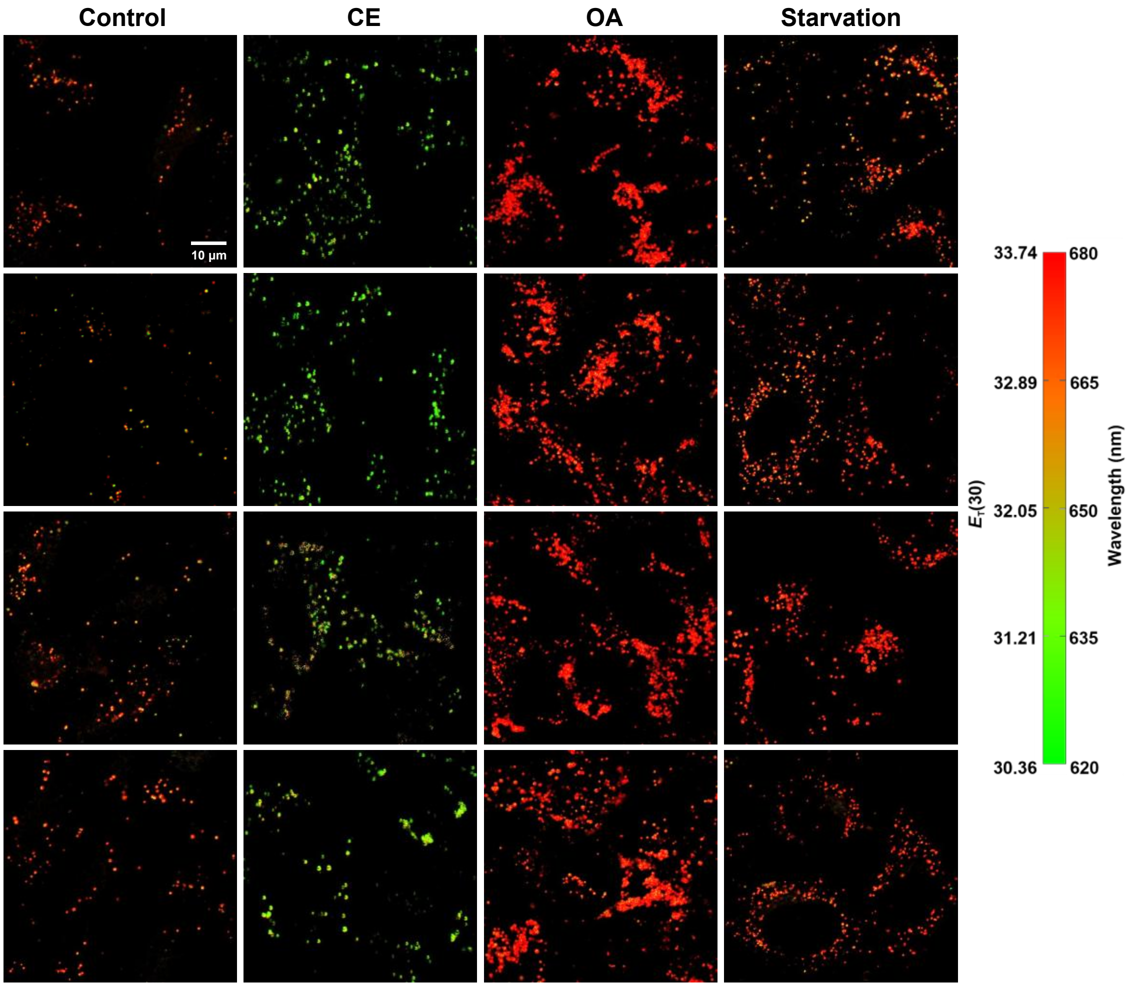

Figure S23. HSFI images of HeLa cells stained with **Lipi-PS** under various stimulations. Scale bar: 10 μm.

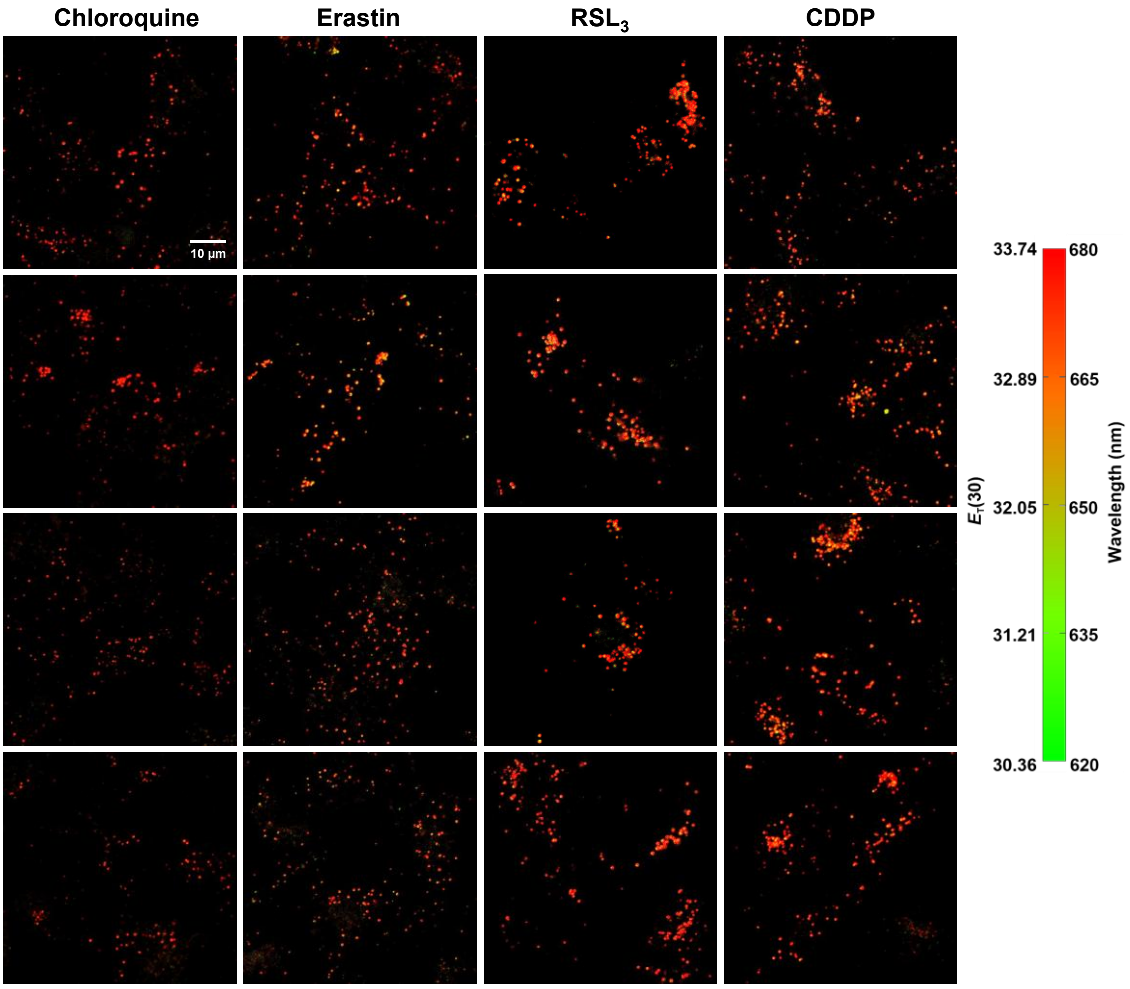

Figure S24. HSFI images of HeLa cells stained with **Lipi-PS** under various stimulations. Scale bar: 10 μm.

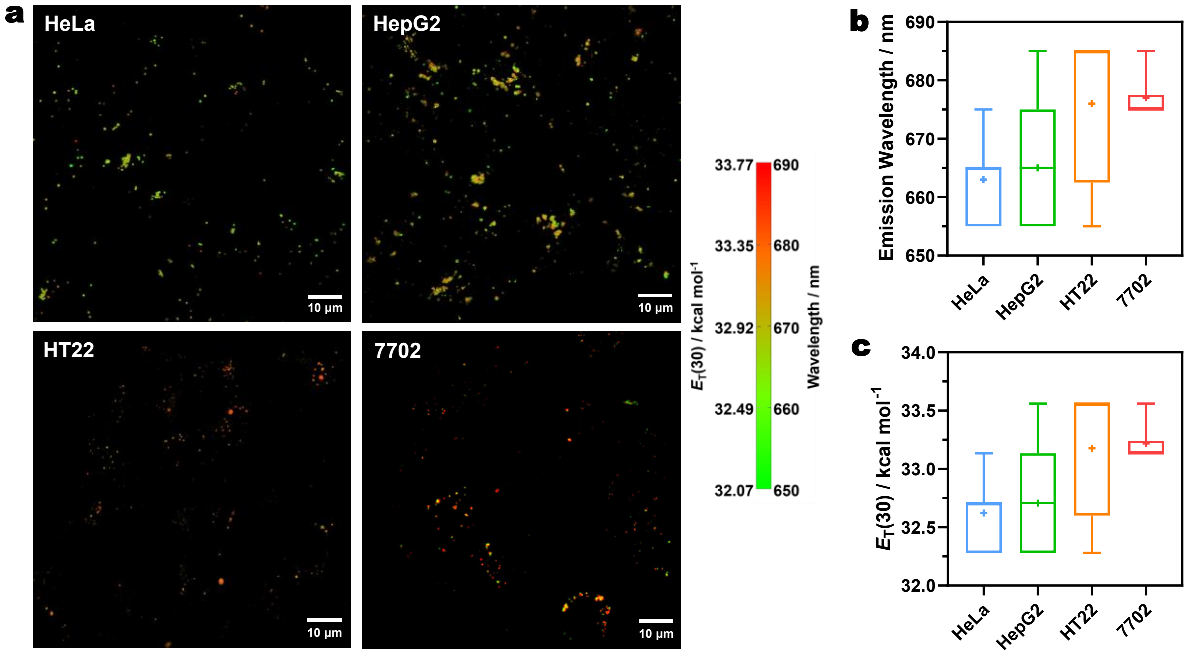

Figure S25. HSFI of various cells stained with **Lipi-PS**: (a) HSFI images of various cells, scale bar: 10 μm; (b) the statistic results of emission wavelength; (c) the statistic results of polarity index $E_T(30)$.

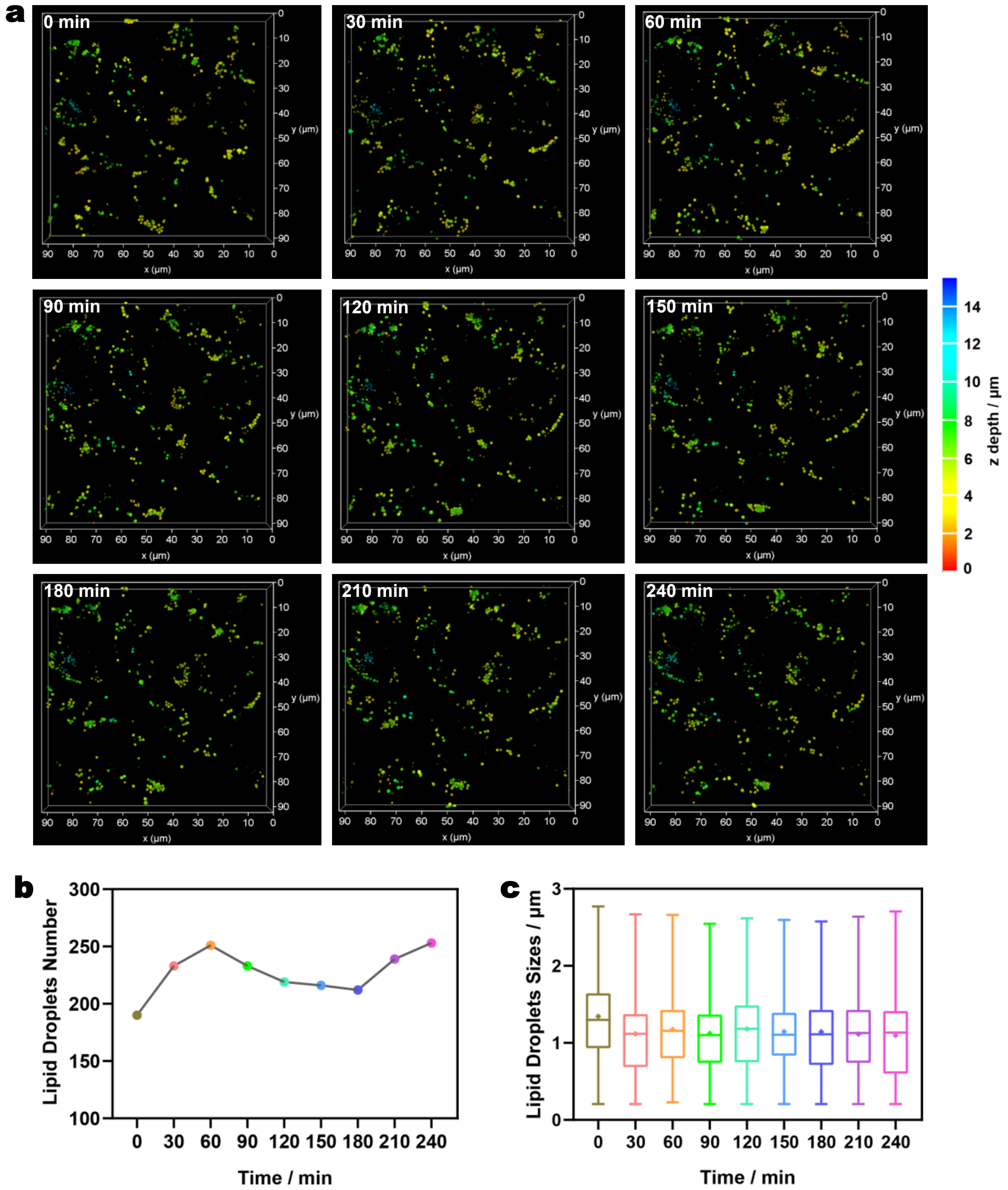

Figure S26. Time-lapse 3D confocal imaging of living HeLa cells labelled with **Lipi-PS** under the stimulation of OA: (a) the 3D images at different time spots (0–240 min); (b) the variation of LDs number with time; (c) the variation of LDs sizes with time.

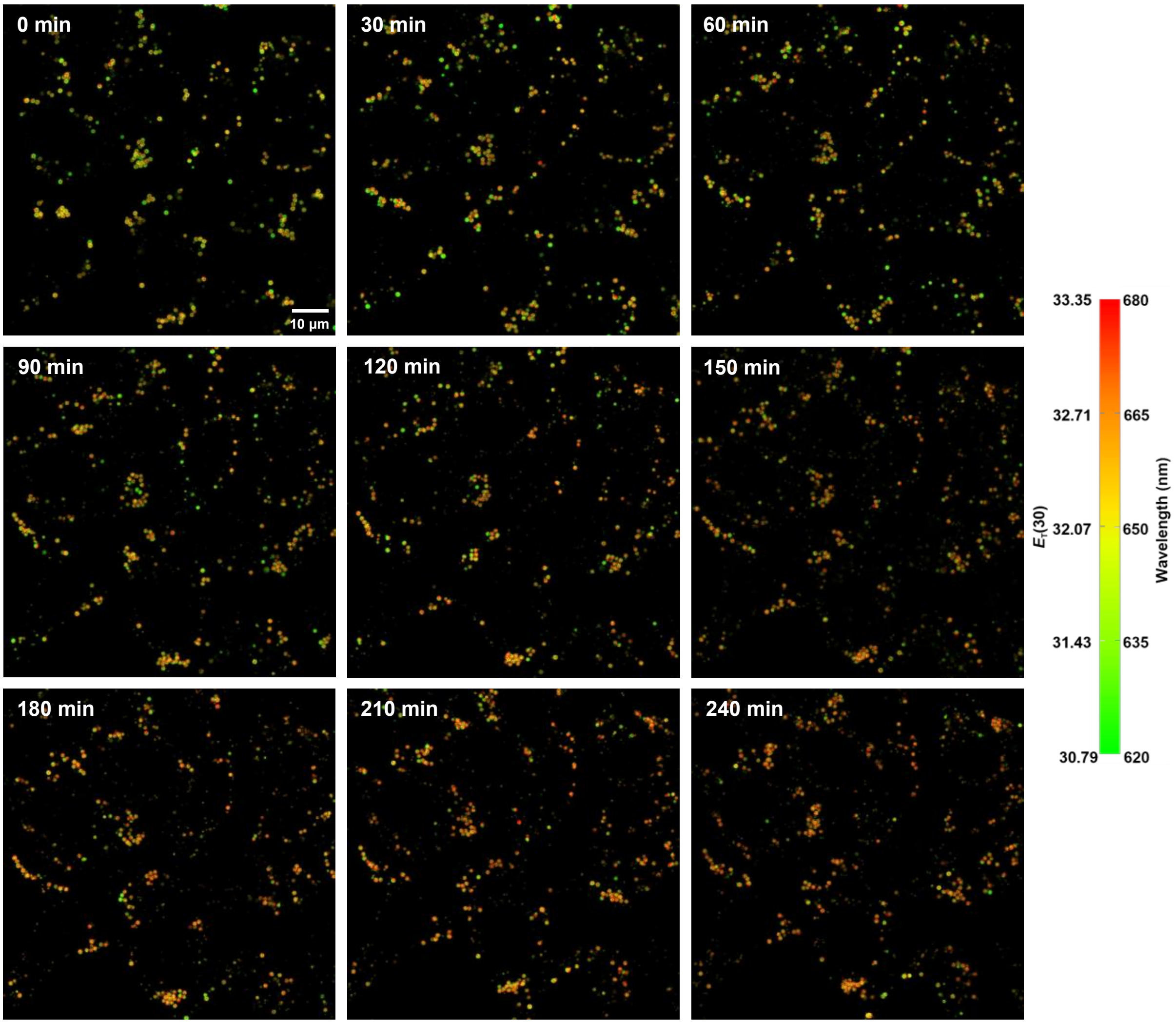

Figure S27. Time-lapse HSFI of living HeLa cells labelled with **Lipi-PS** under the stimulation of OA. Scale bar: 10 μm.

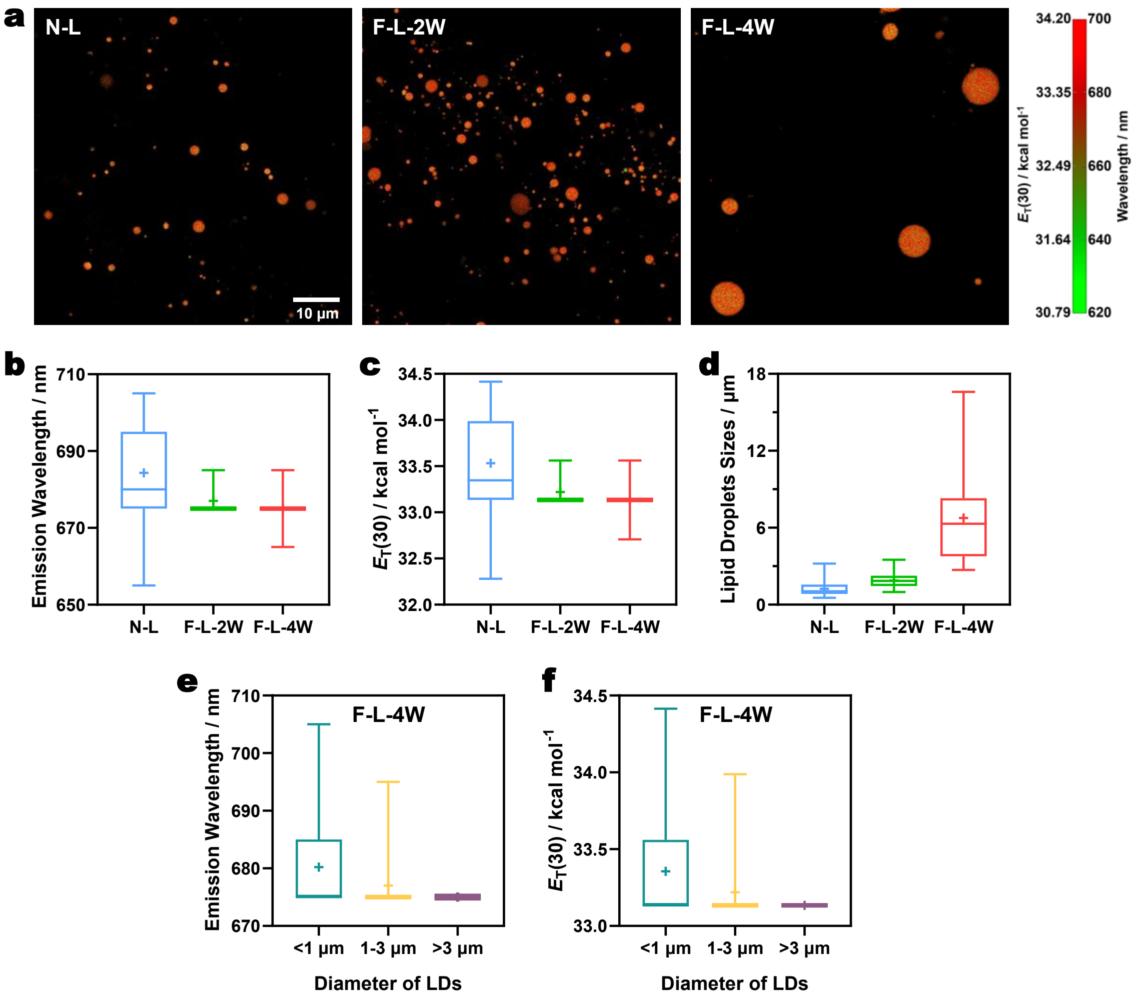

Figure S28. HSFI of mouse liver tissues stained with **Lipi-PS**: (a) HSFI images of normal liver tissues and NAFLD tissues of different time (2-week and 4-week); scale bar: 10 µm; (b–d) the statistic results of emission wavelength, polarity and size of LDs in various liver tissues; (e–f) the statistic results of emission wavelength and polarity of LDs with various diameters in 4-week NAFLD tissues.

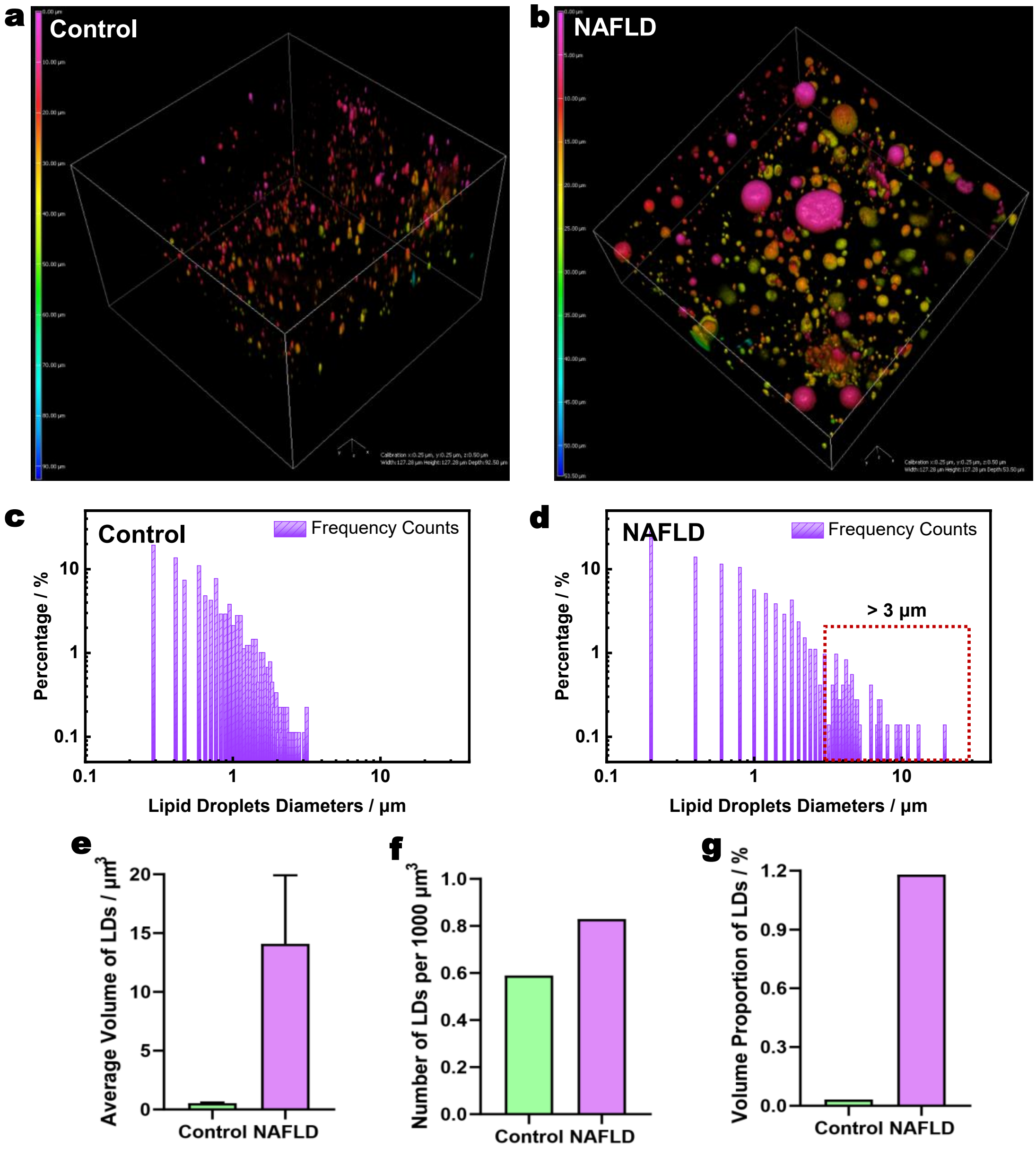

Figure S29. Two-photon 3D imaging of mice liver tissues stained with **Lipi-PS**: (a–b) images of control or NAFLD (4-week) tissues; (c–d) the LDs diameter distribution of two tissues; (e) the average volume of LDs in two tissues; (f) the density of LDs in two tissues in terms of the number of LDs per 1000 μm^3; (g) the volume proportion of LDs in two tissues.

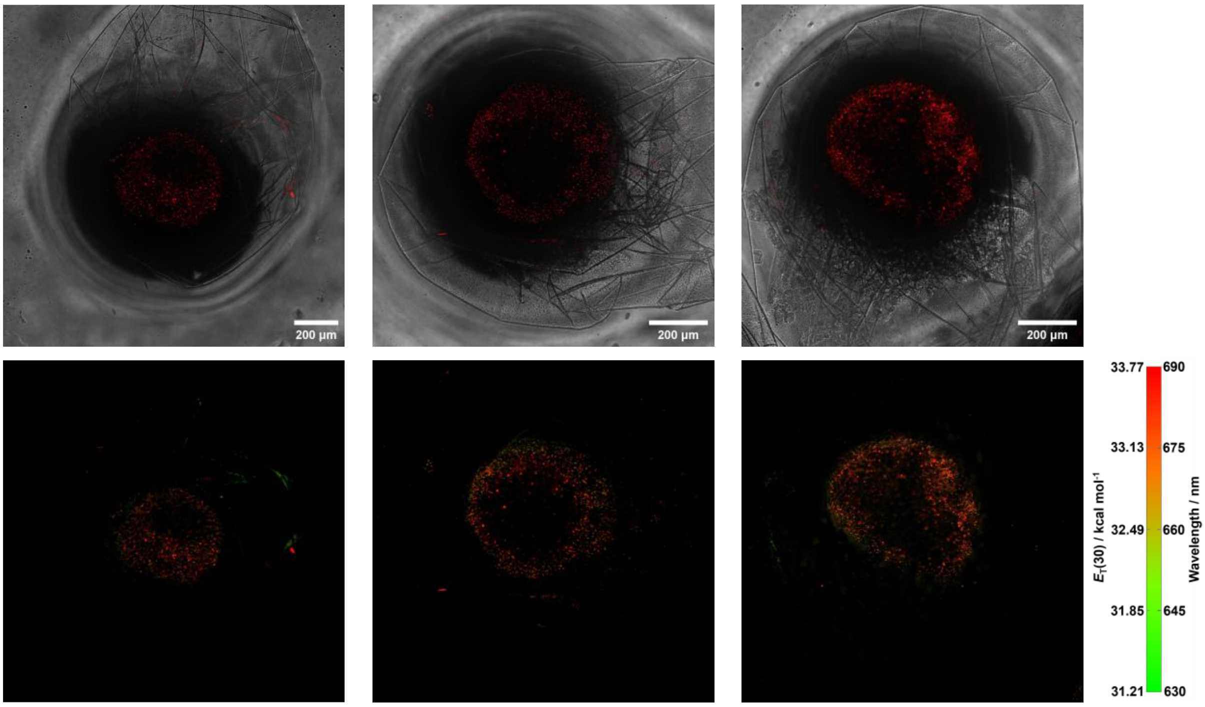

Figure S30. HSFI of zebrafish on 0 dpf (oosperm) stained with **Lipi-PS**: the first row is the merged images of bright field and fluorescence images; the second row is the HSFI images. Scale bar: 200 µm.

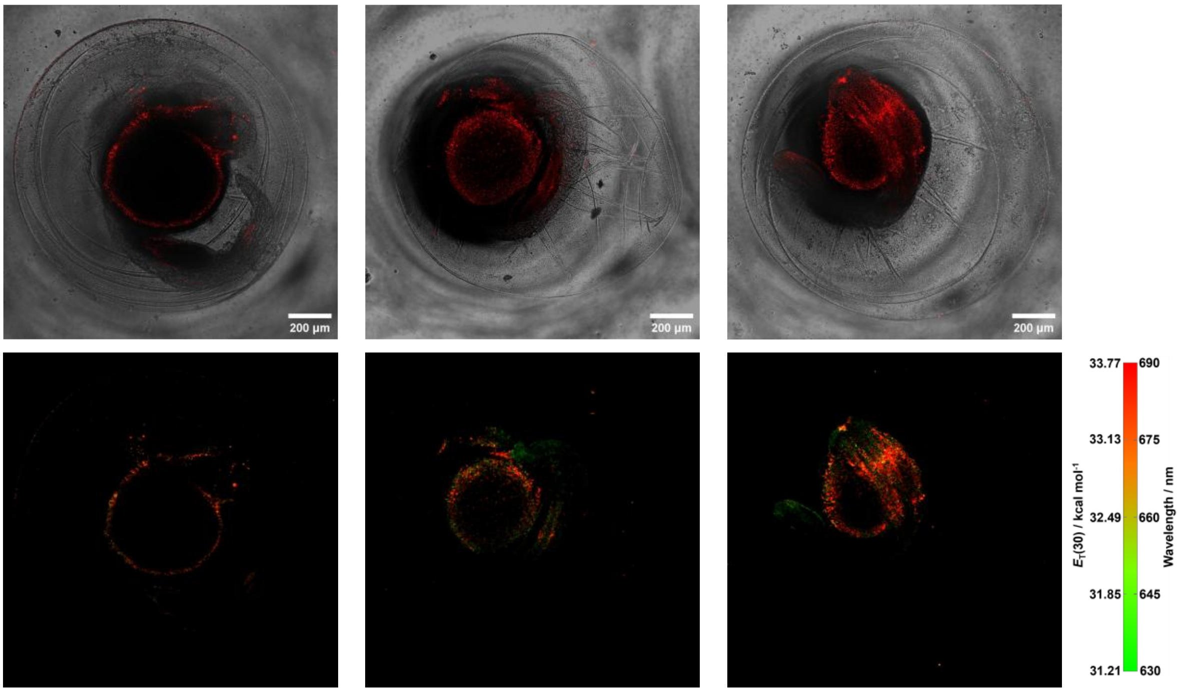

Figure S31. HSFI of zebrafish on 1 dpf stained with **Lipi-PS**: the first row is the merged images of bright field and fluorescence images; the second row is the HSFI images. Scale bar: 200 µm.

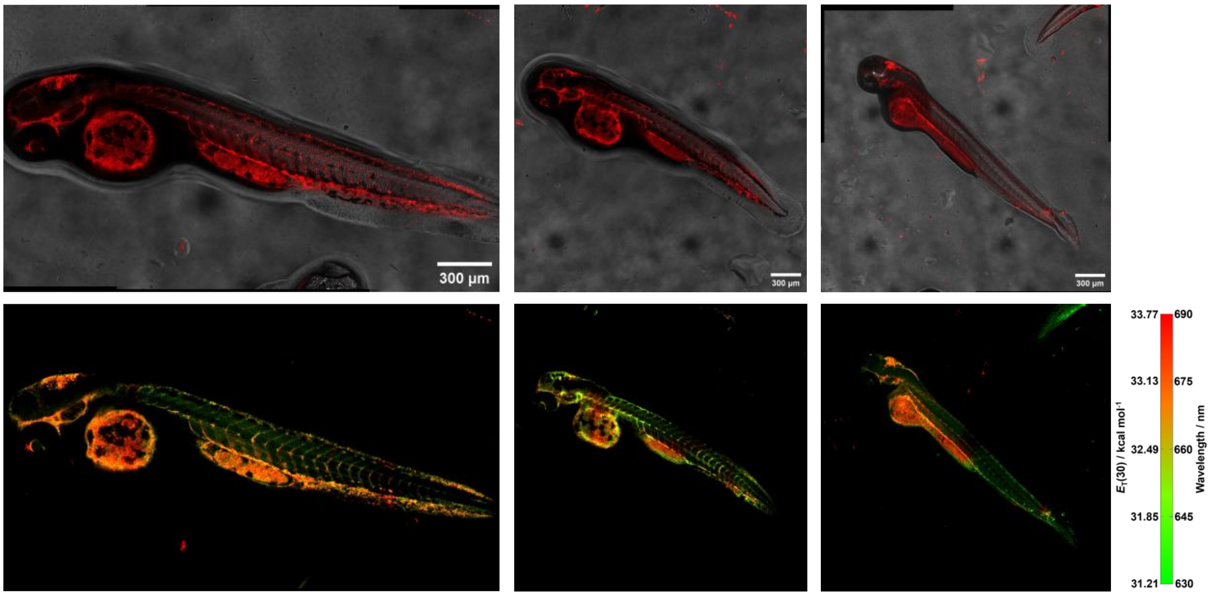

Figure S32. HSFI of zebrafish on 2 dpf stained with **Lipi-PS**: the merged images of bright field and fluorescence images and the corresponding HSFI images. Scale bar: 300 μm.

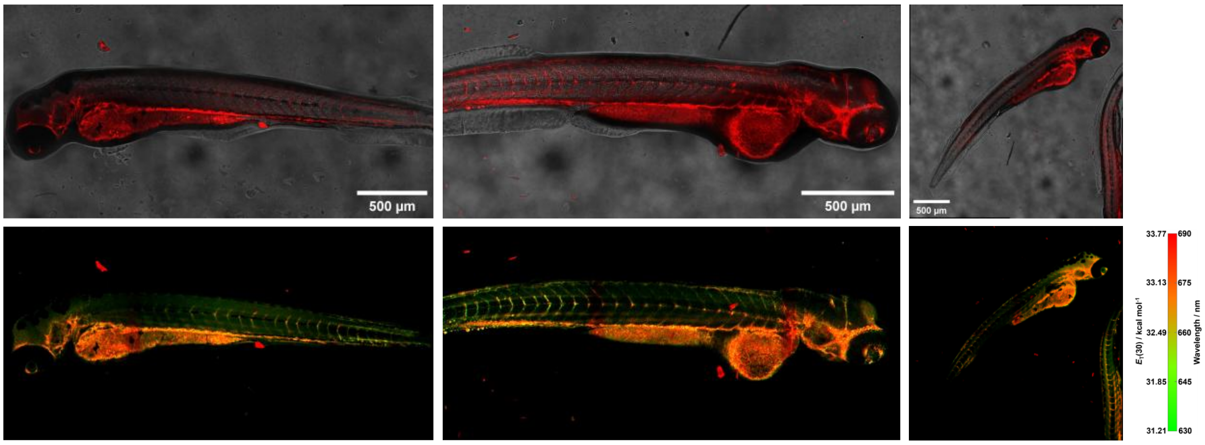

Figure S33. HSFI of zebrafish on 3 dpf stained with **Lipi-PS**: the merged images of bright field and fluorescence images and the corresponding HSFI images. Scale bar: 500 μm.

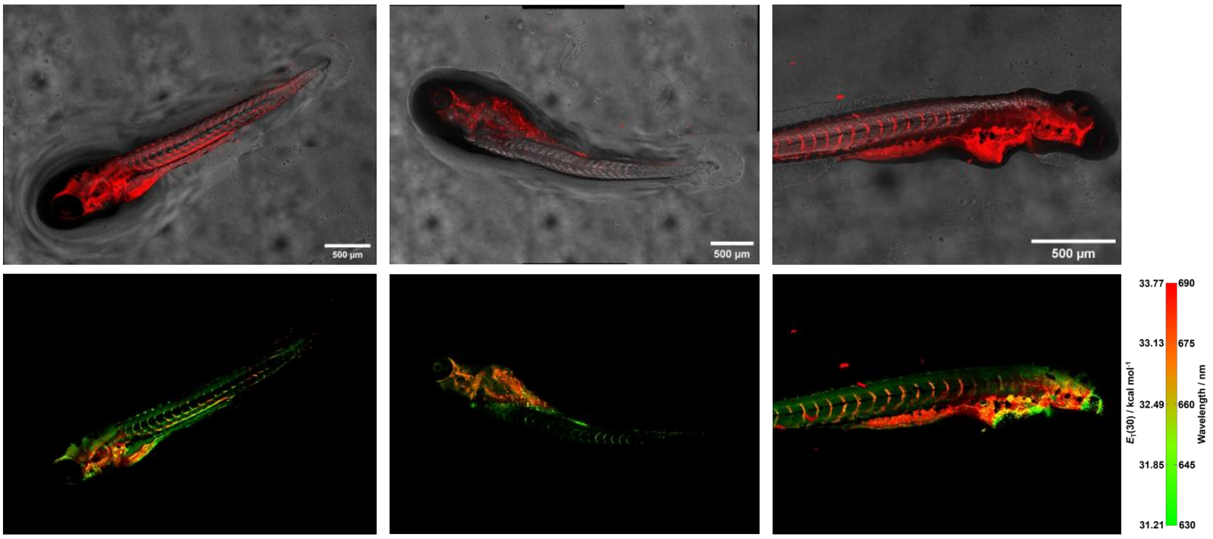

Figure S34. HSFI of zebrafish on 7 dpf stained with **Lipi-PS**: the merged images of bright field and fluorescence images and the corresponding HSFI images. Scale bar: 500 μm.

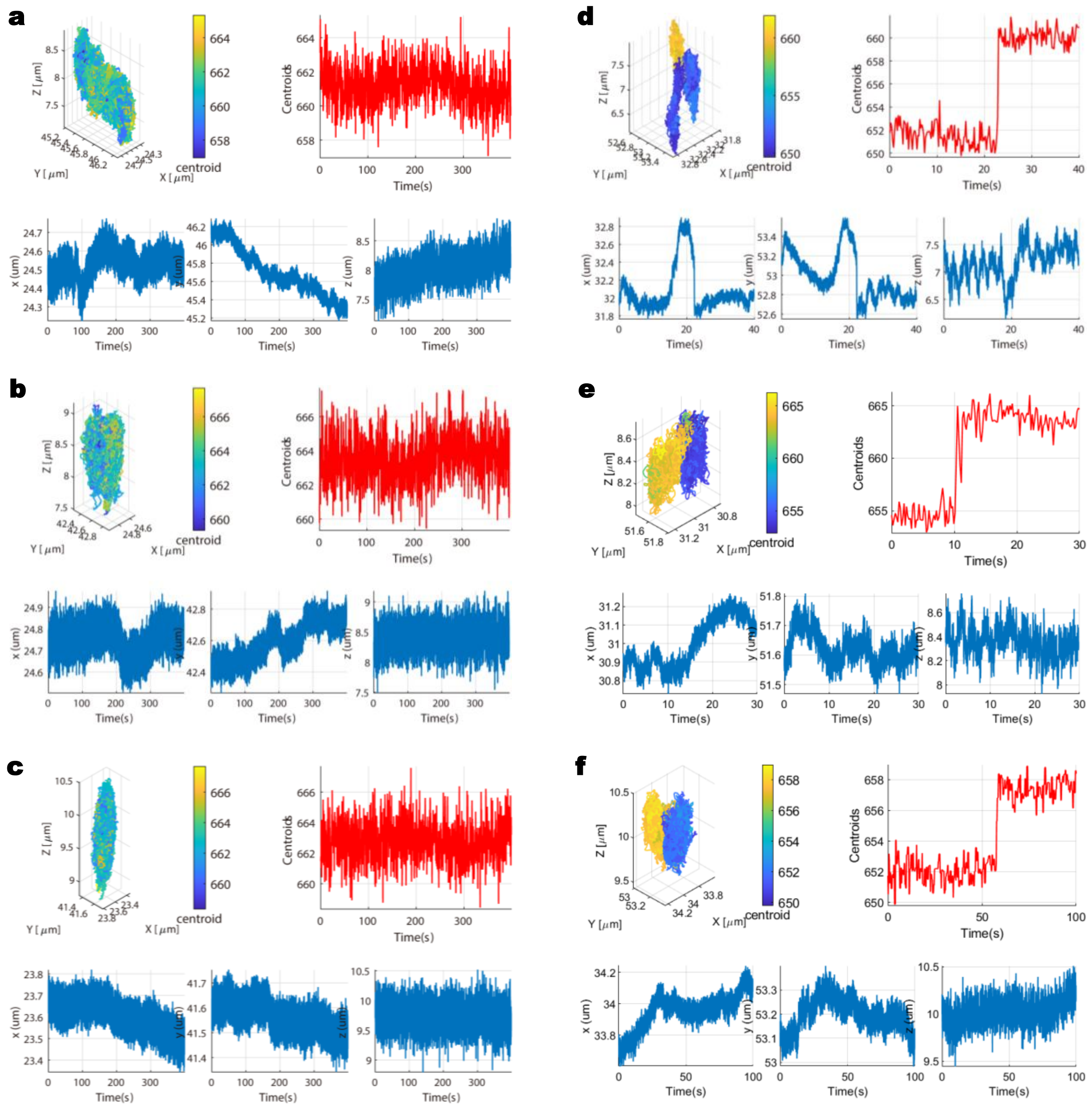

Figure S35. The trajectory and spectral change of 5D dynamic tracking of single LD stained with **Lipi-PS**: (a–c) LDs in control groups; (d–f) LDs in stimulation groups.

7. NMR and MS of Synthesized Molecules

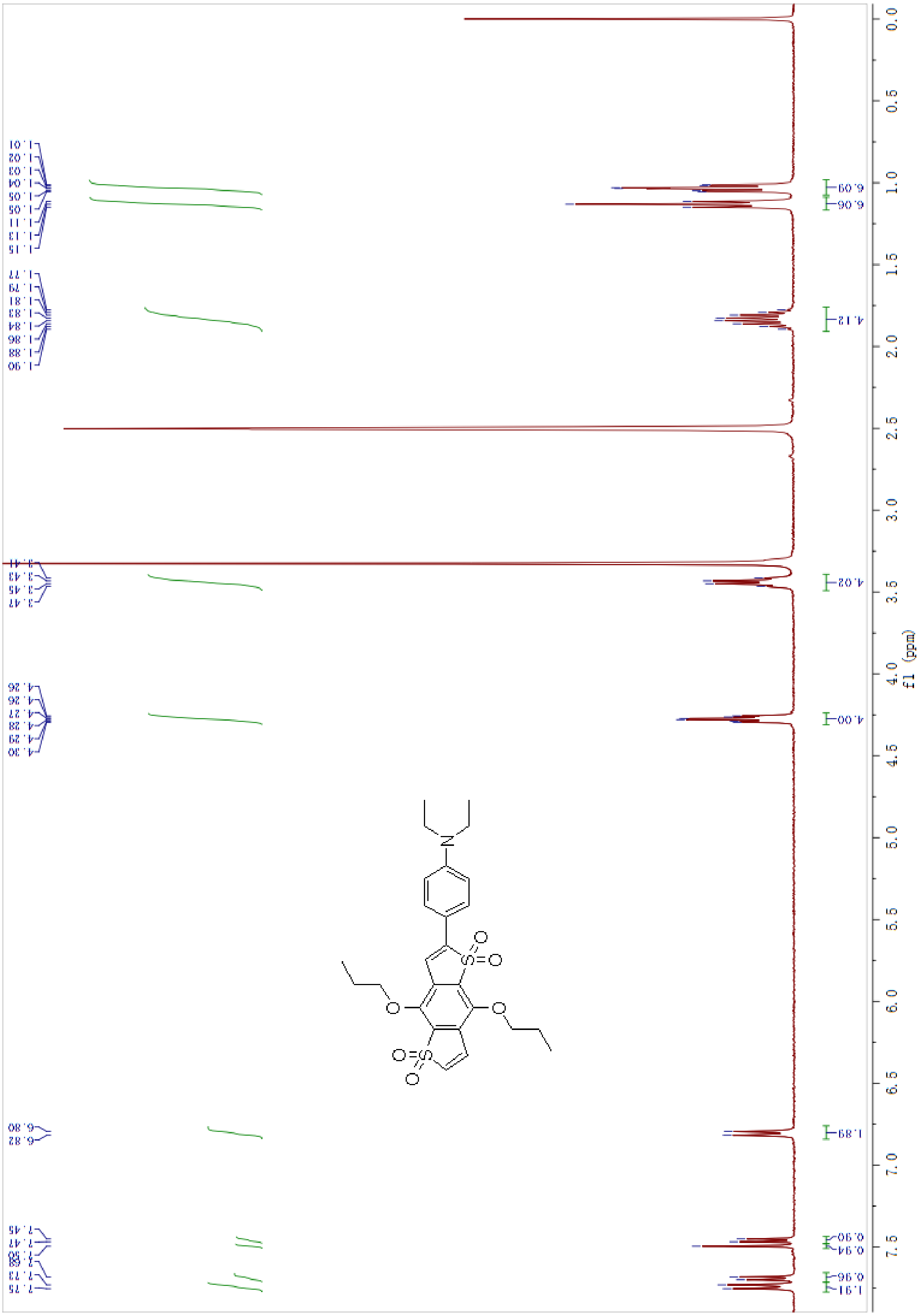

NMR-1. ^{1}H NMR spectrum of compound **1** (400 MHz, DMSO-d_6).

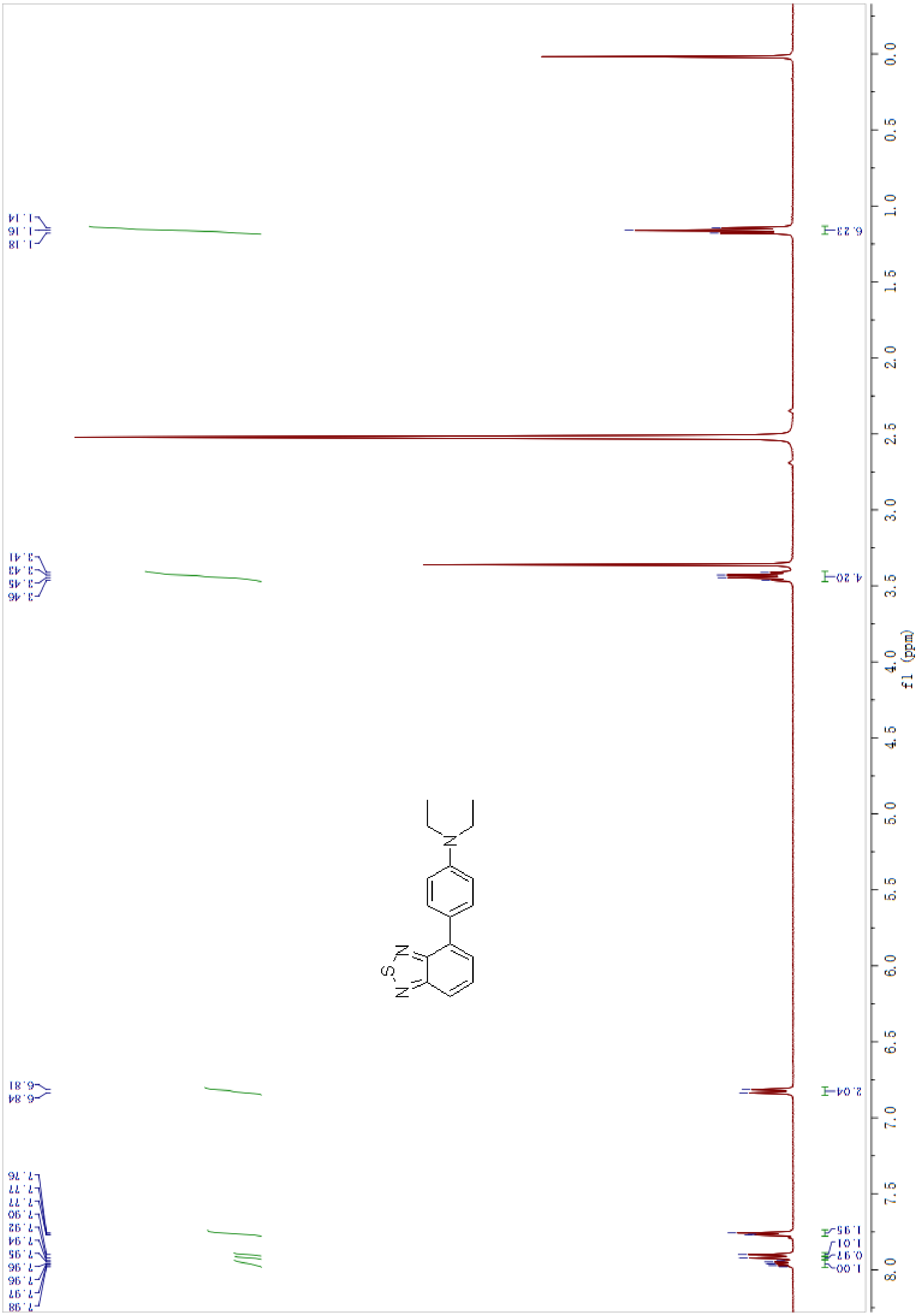

NMR-2. ^{1}H NMR spectrum of compound **2** (400 MHz, DMSO-d_6).

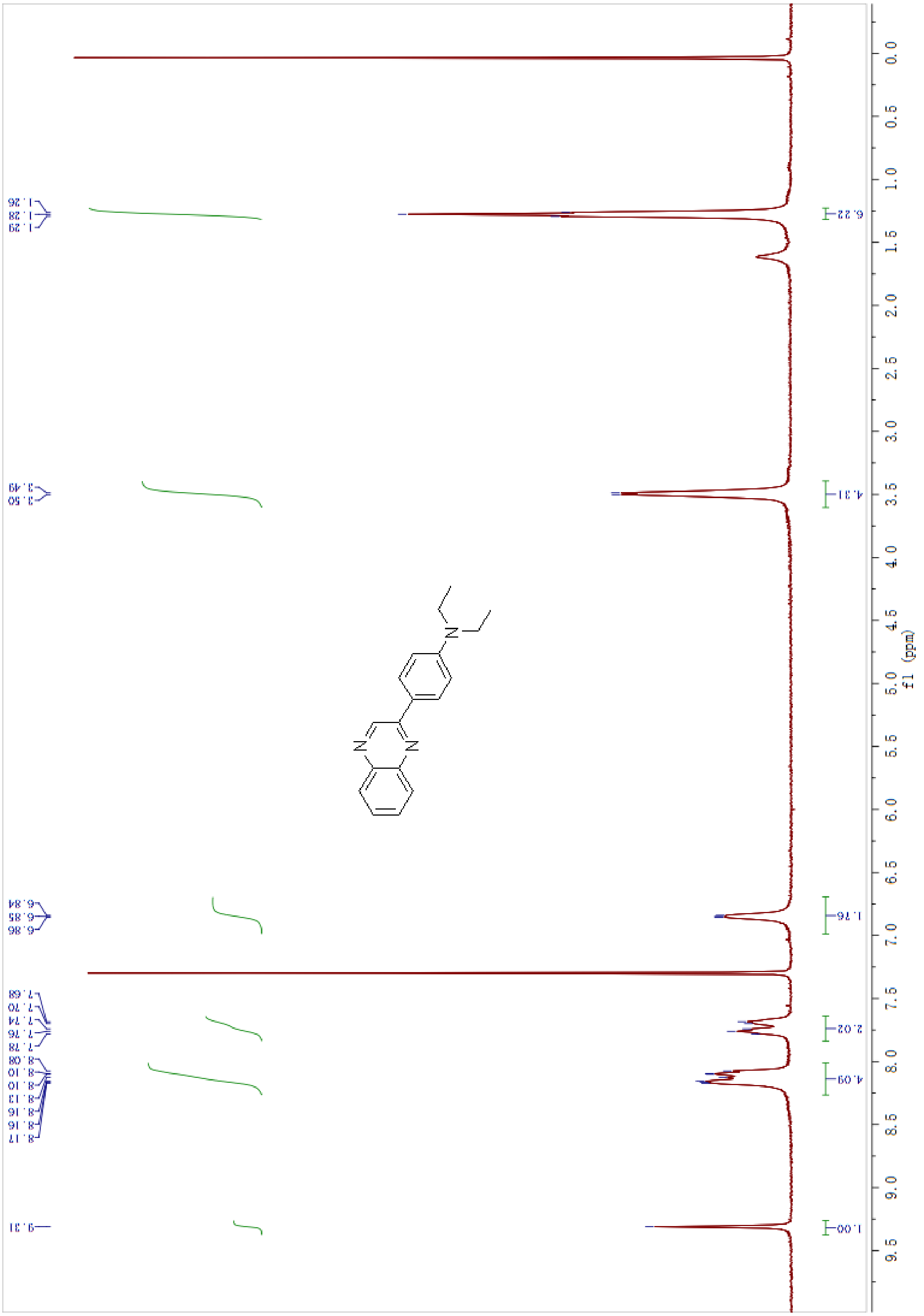

NMR-3. ^{1}H NMR spectrum of compound **3** (400 MHz, $CDCl_3$).

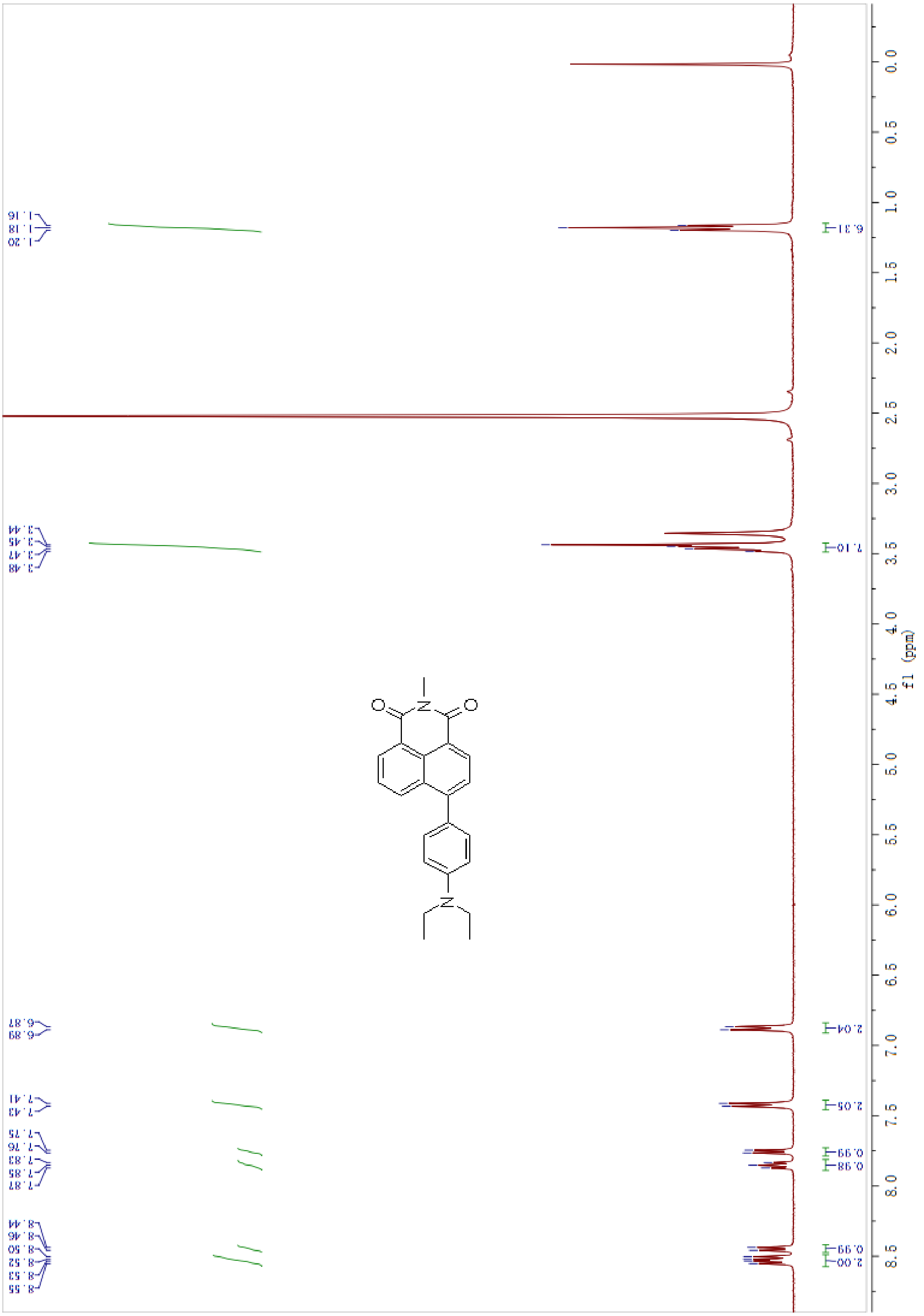

NMR-4. ^{1}H NMR spectrum of compound **4** (400 MHz, DMSO-d_6).

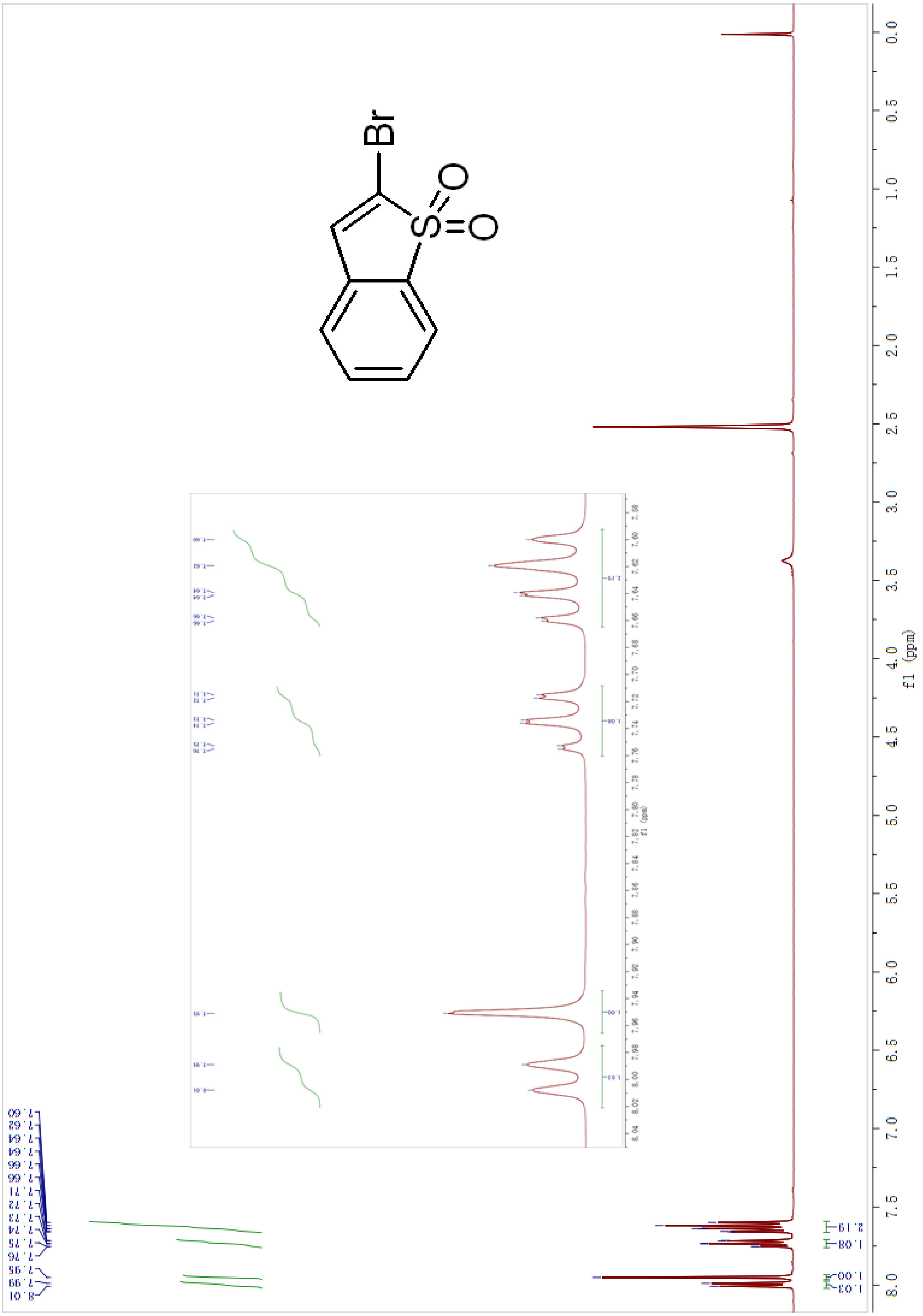

NMR-5. ^{1}H NMR spectrum of compound **5a** (400 MHz, DMSO-d_6).

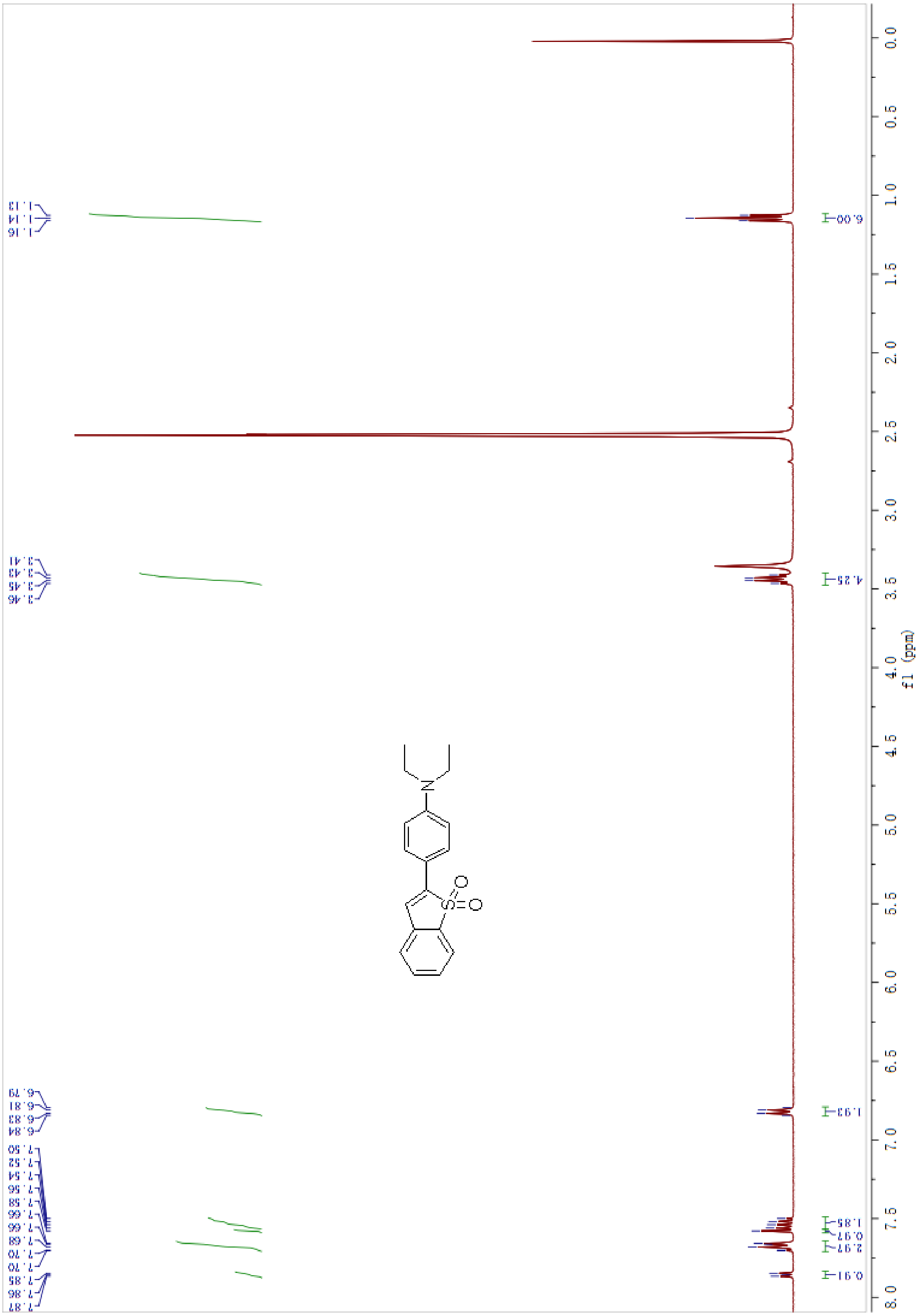

NMR-6. ^{1}H NMR spectrum of compound **5** (400 MHz, DMSO-d_6).

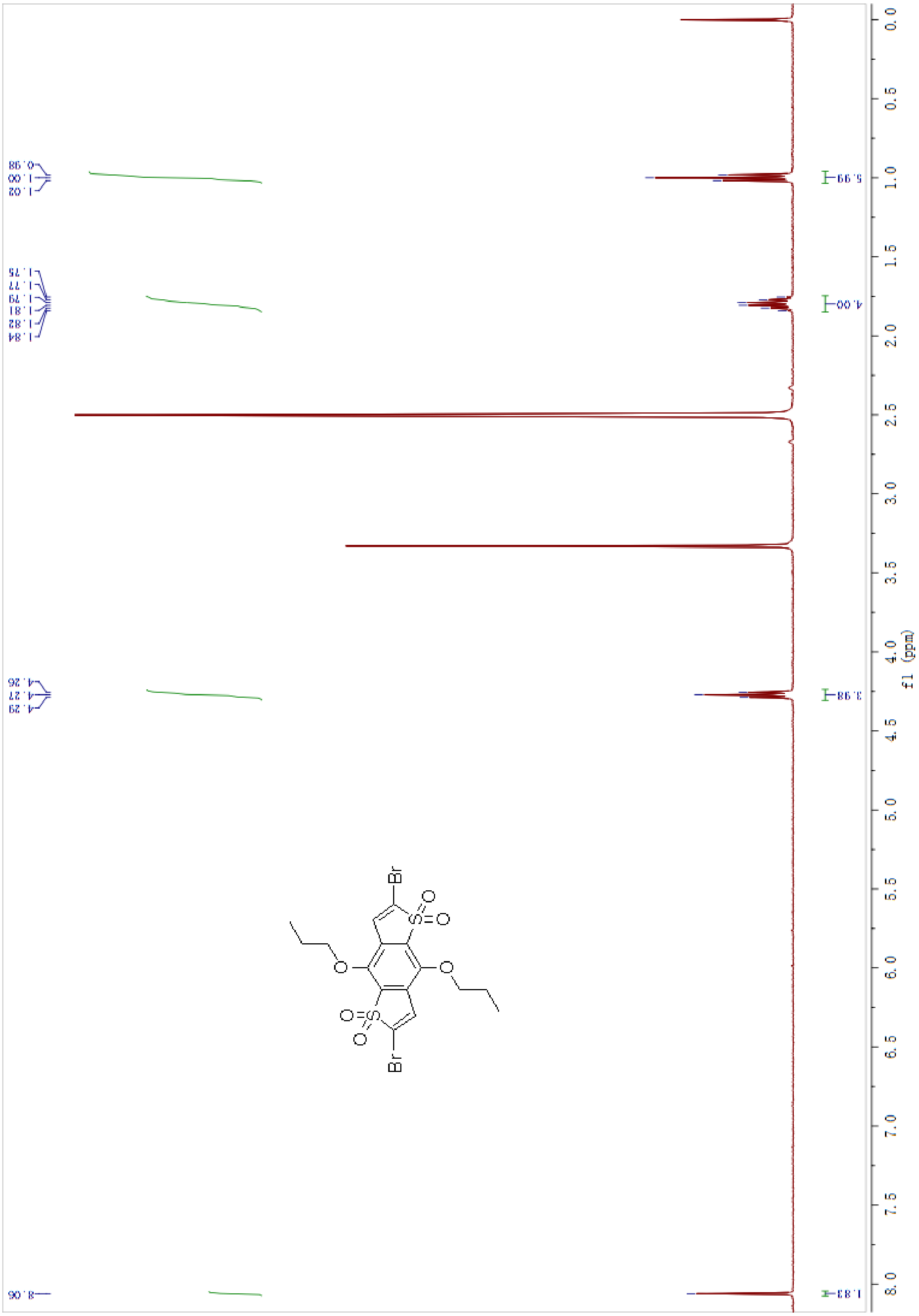

NMR-7. ^{1}H NMR spectrum of compound **C$_1$** (400 MHz, DMSO-d_6).

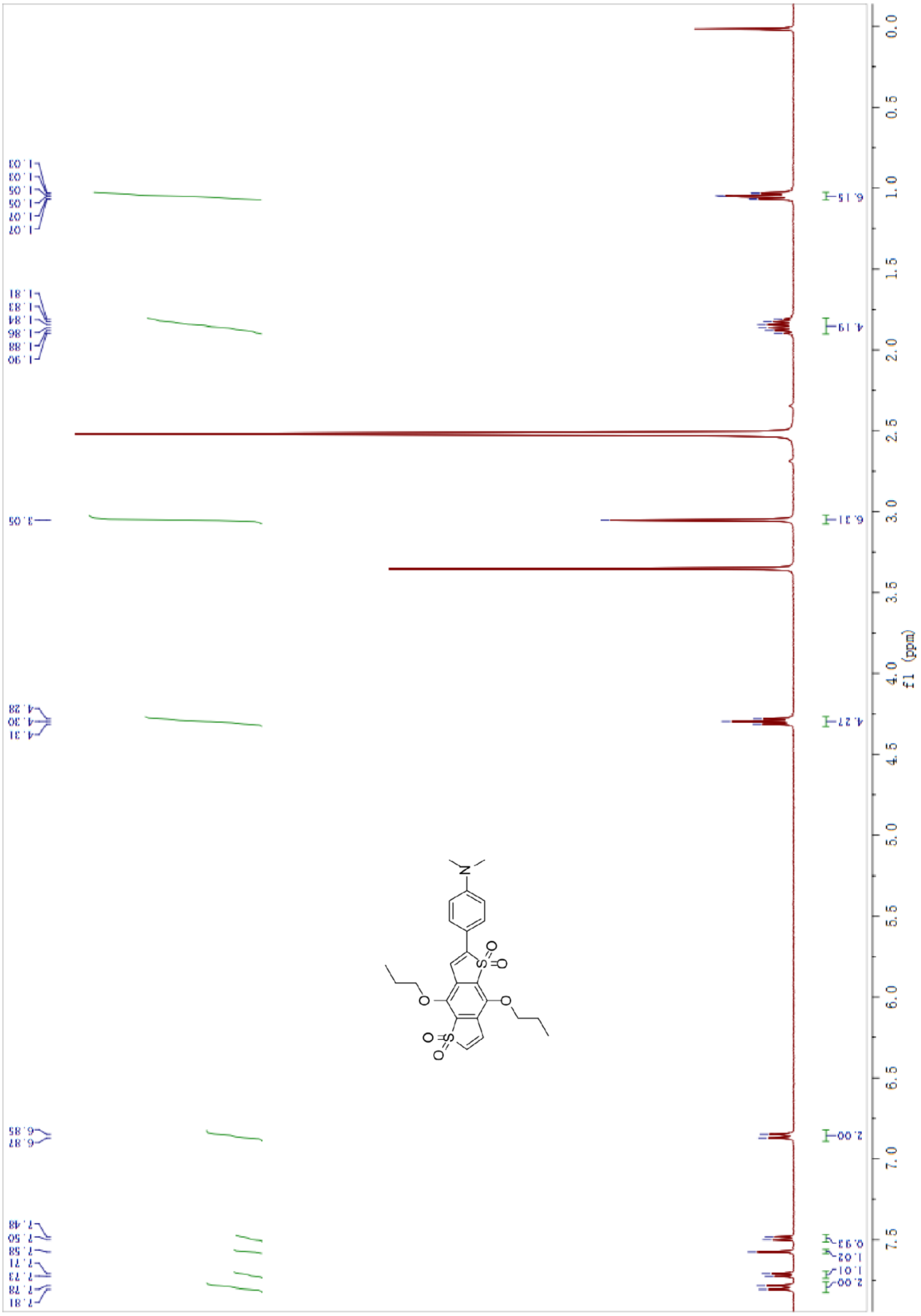

NMR-8. ^{1}H NMR spectrum of compound **6** (400 MHz, DMSO-d_6).

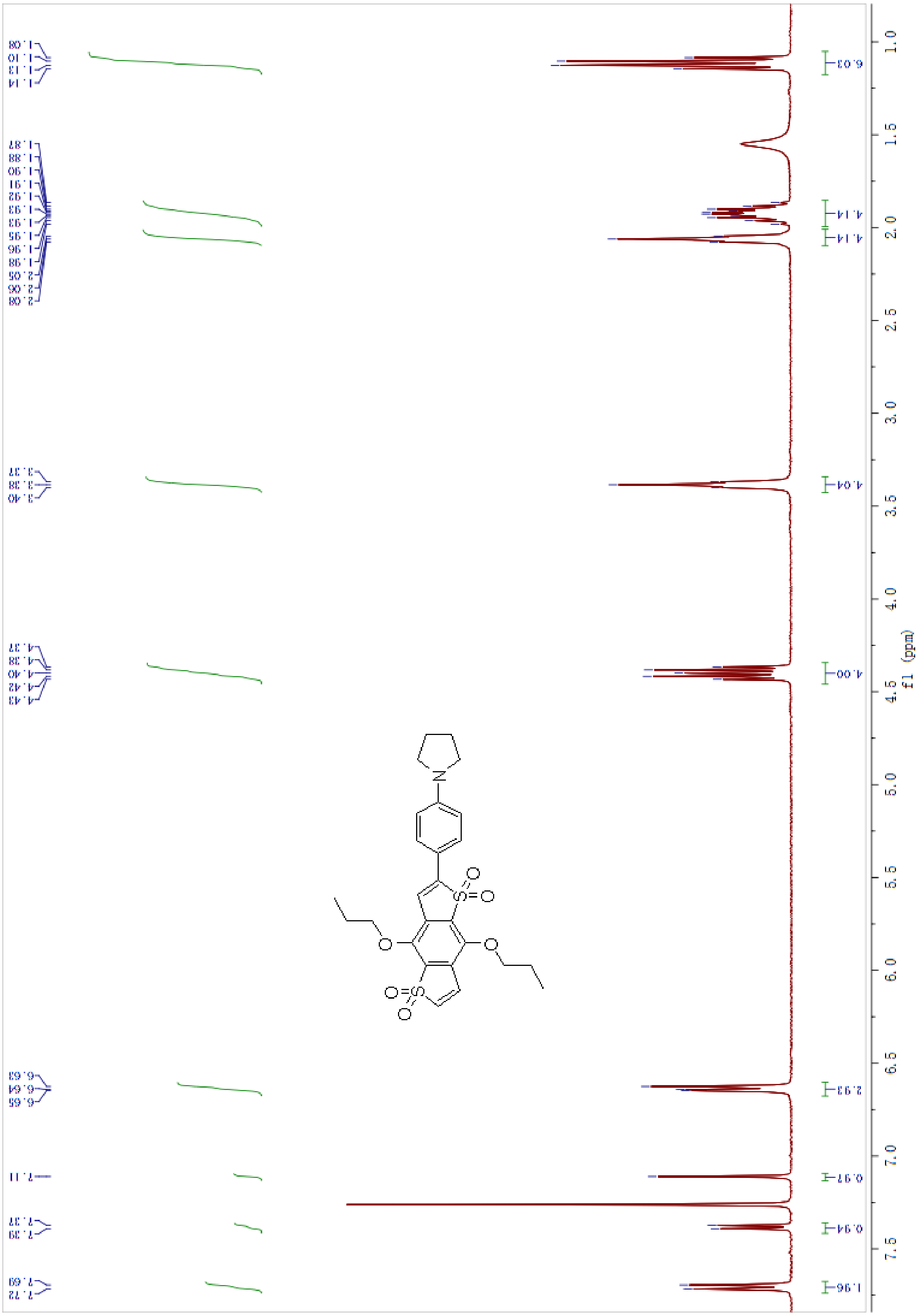

NMR-9. ^{1}H NMR spectrum of compound **7** (400 MHz, $CDCl_3$).

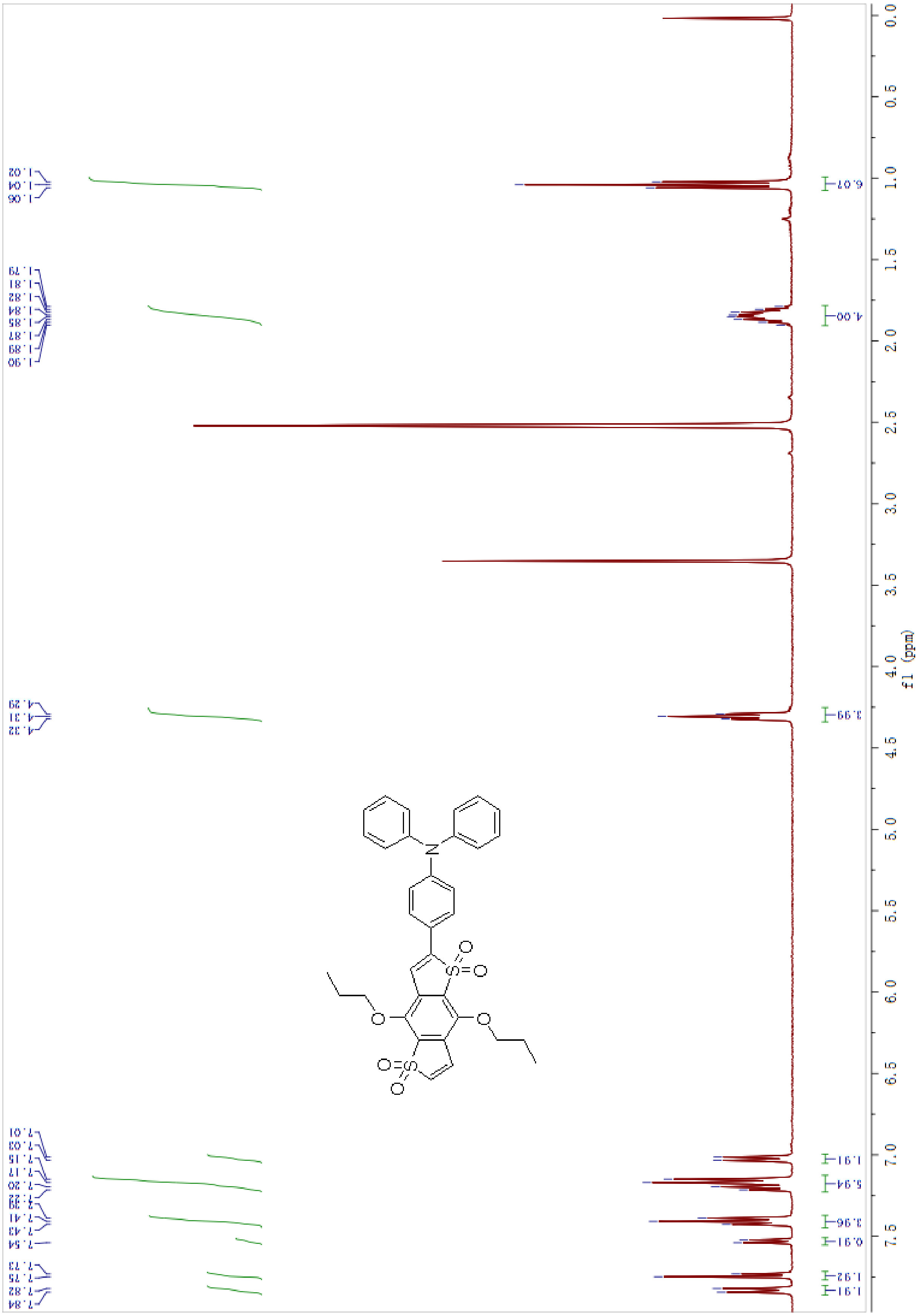

NMR-10. ^{1}H NMR spectrum of compound **8** (400 MHz, DMSO-d_6).

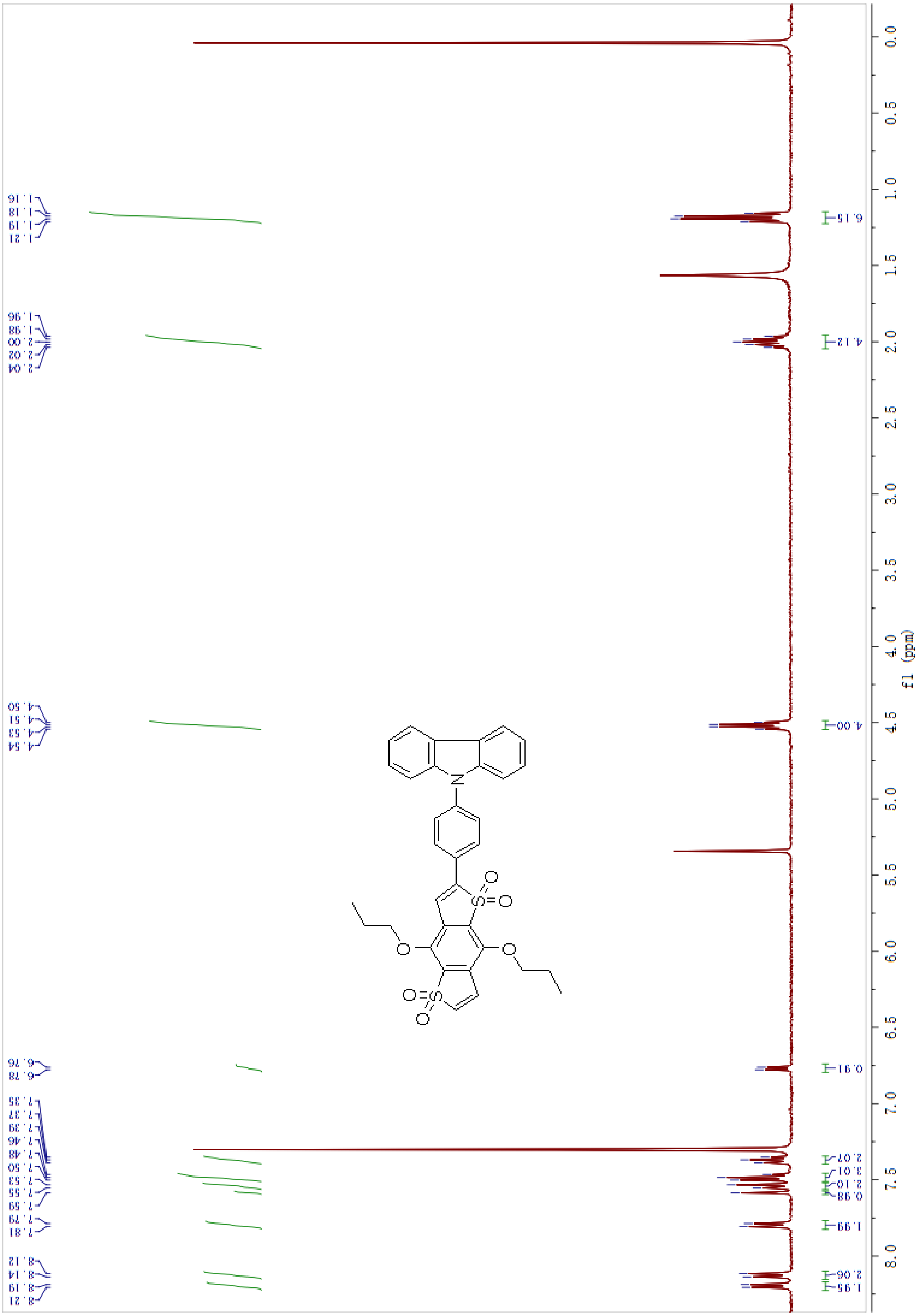

NMR-11. ^{1}H NMR spectrum of compound **9** (400 MHz, $CDCl_3$).

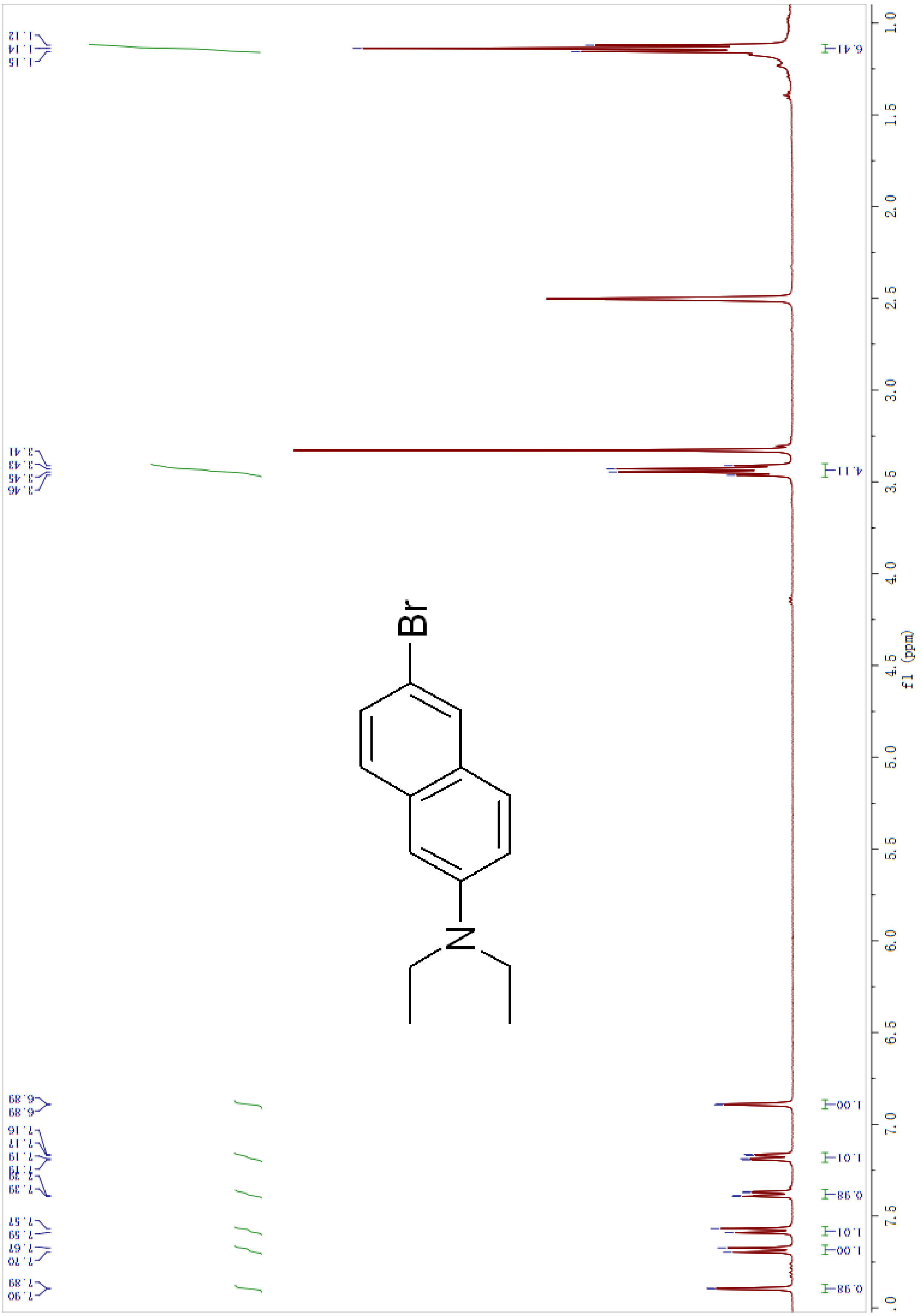

NMR-12. ^{1}H NMR spectrum of compound **10a** (400 MHz, DMSO-d_6).

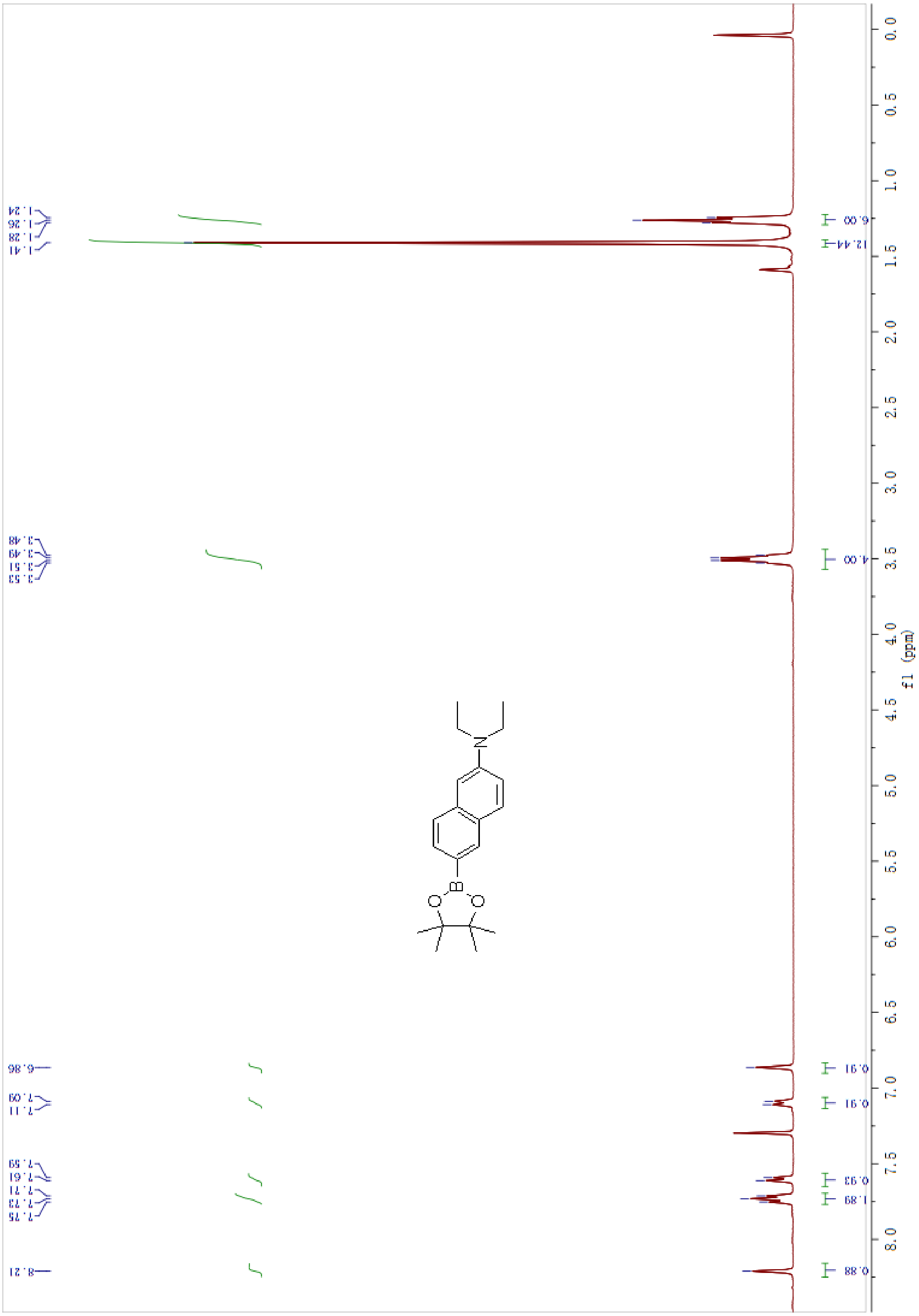

NMR-13. ^{1}H NMR spectrum of compound **10-pin** (400 MHz, $CDCl_3$).

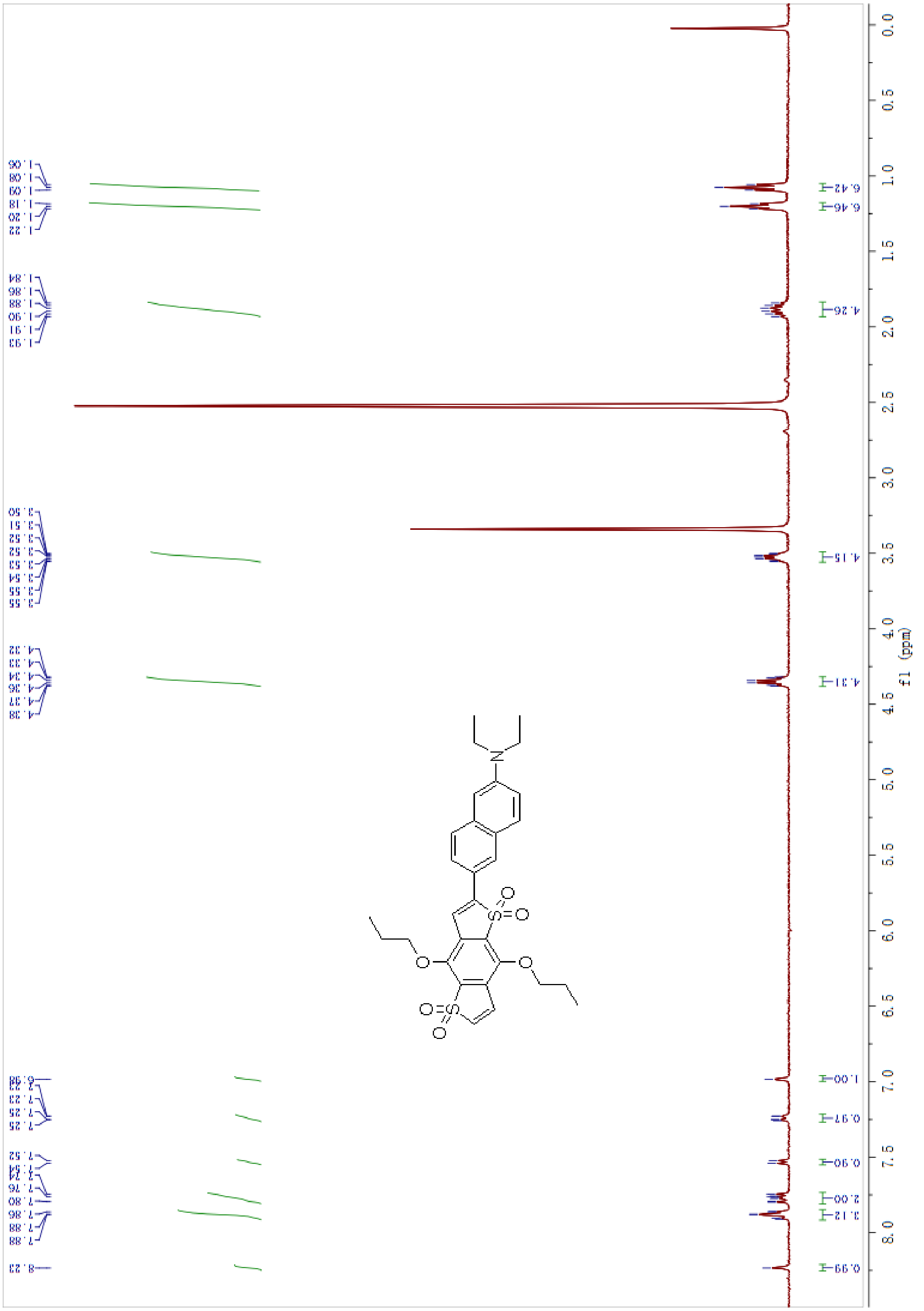

NMR-14. ^{1}H NMR spectrum of compound **10** (400 MHz, DMSO-d_6).

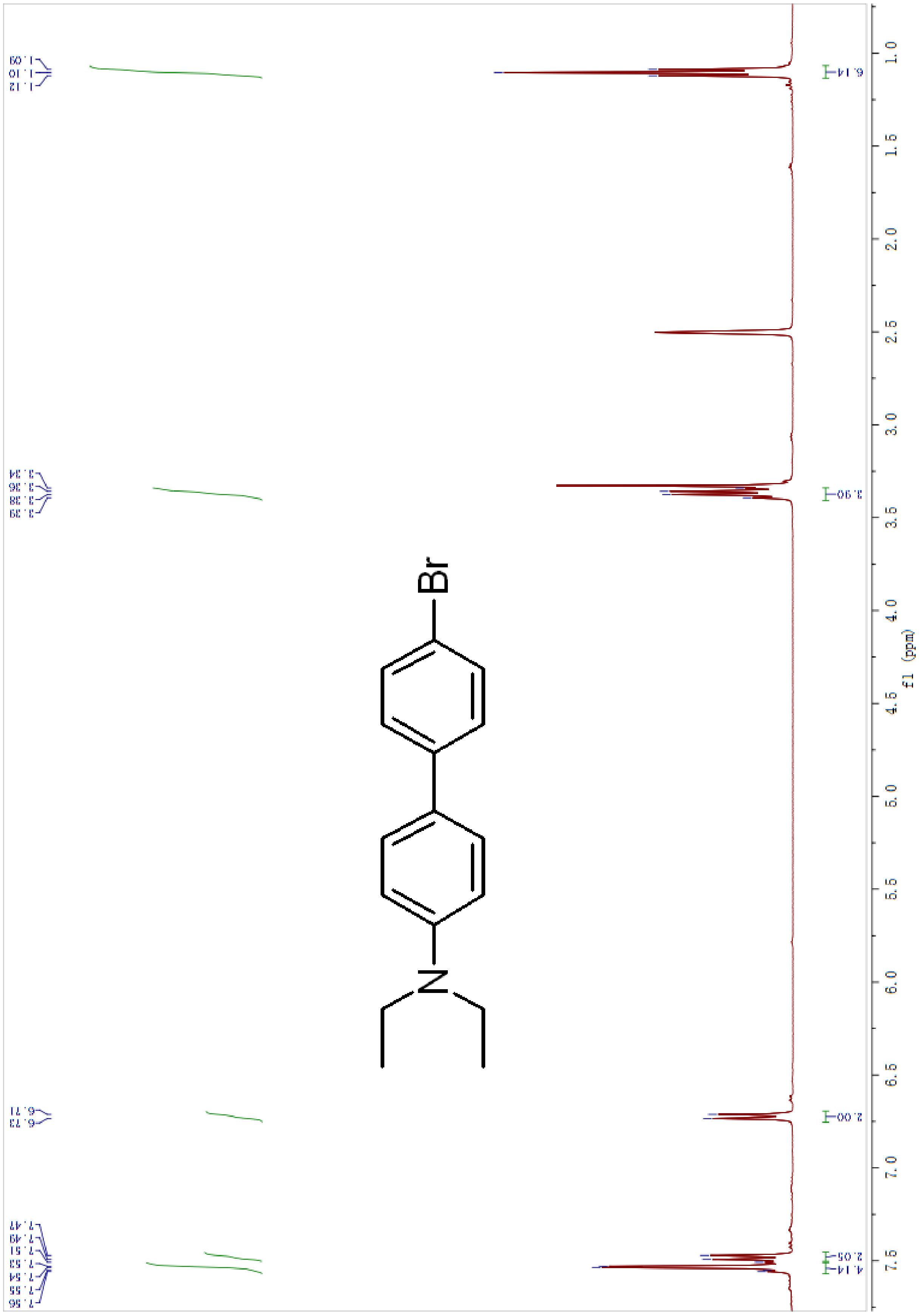

NMR-15. ^{1}H NMR spectrum of compound **11a** (400 MHz, DMSO-d_6).

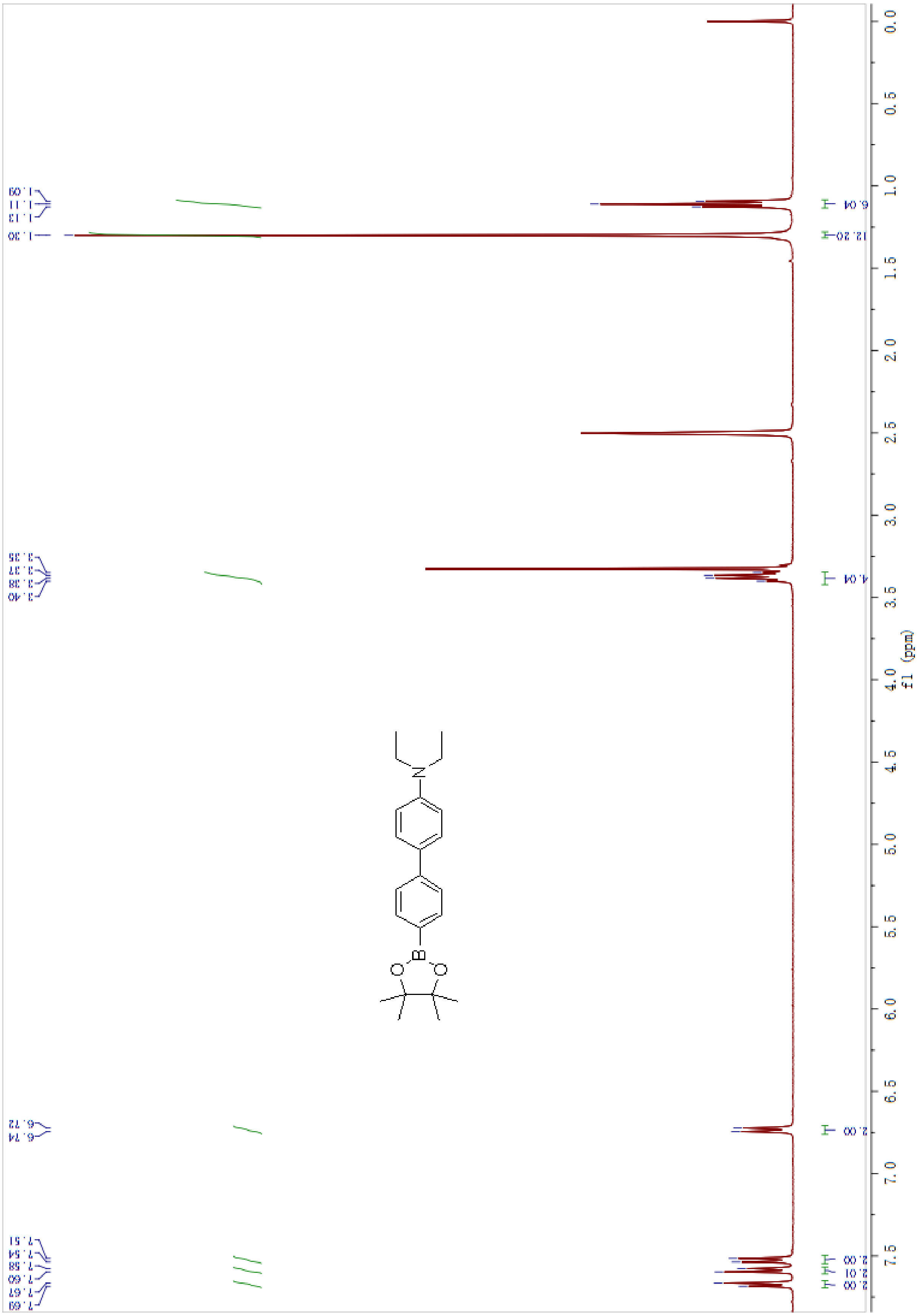

NMR-16. ^{1}H NMR spectrum of compound **11-pin** (400 MHz, DMSO-d_6).

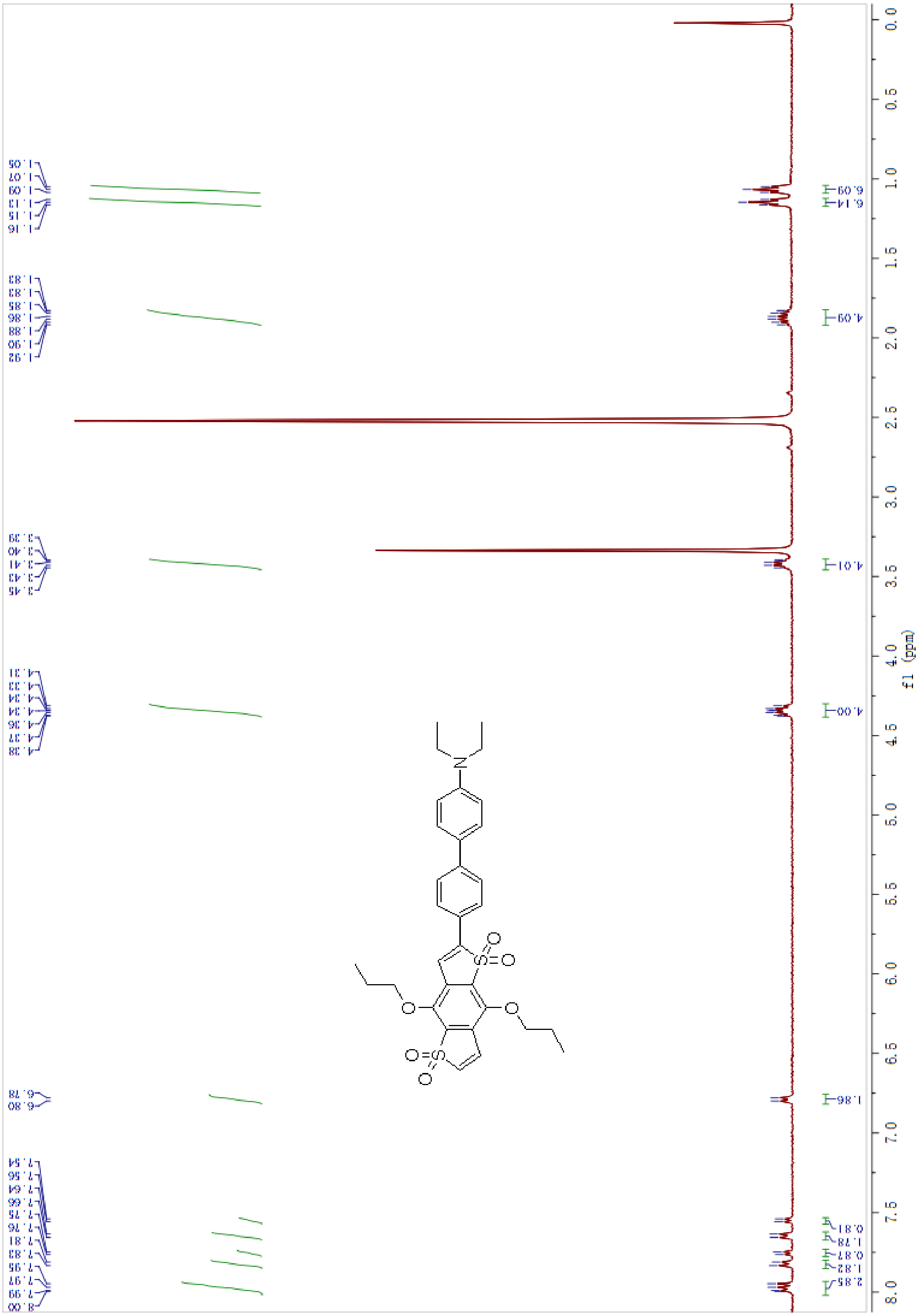

NMR-17. ^{1}H NMR spectrum of compound **11** (400 MHz, DMSO-d_6).

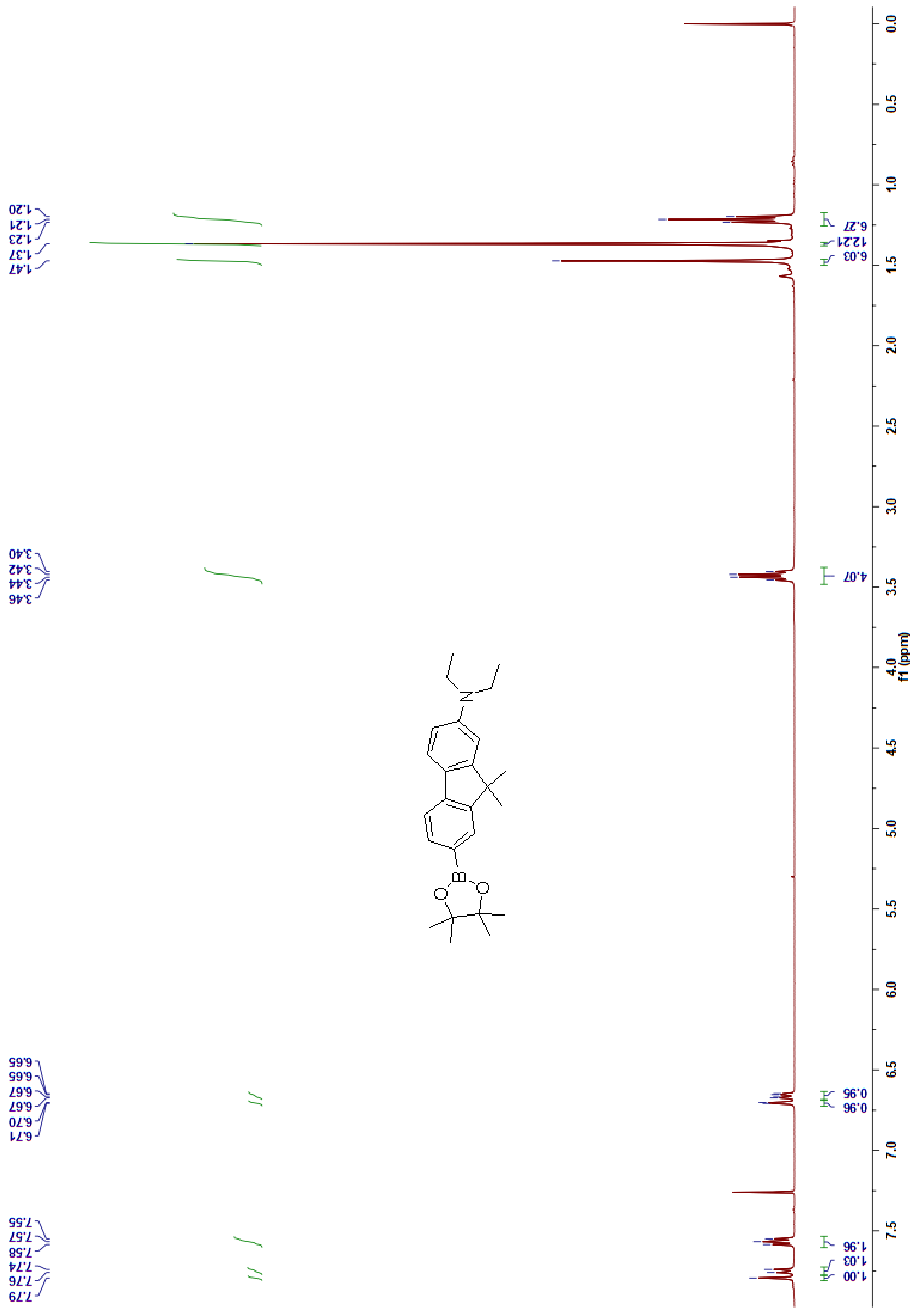

NMR-18. ^{1}H NMR spectrum of compound **12-pin** (400 MHz, $CDCl_3$).

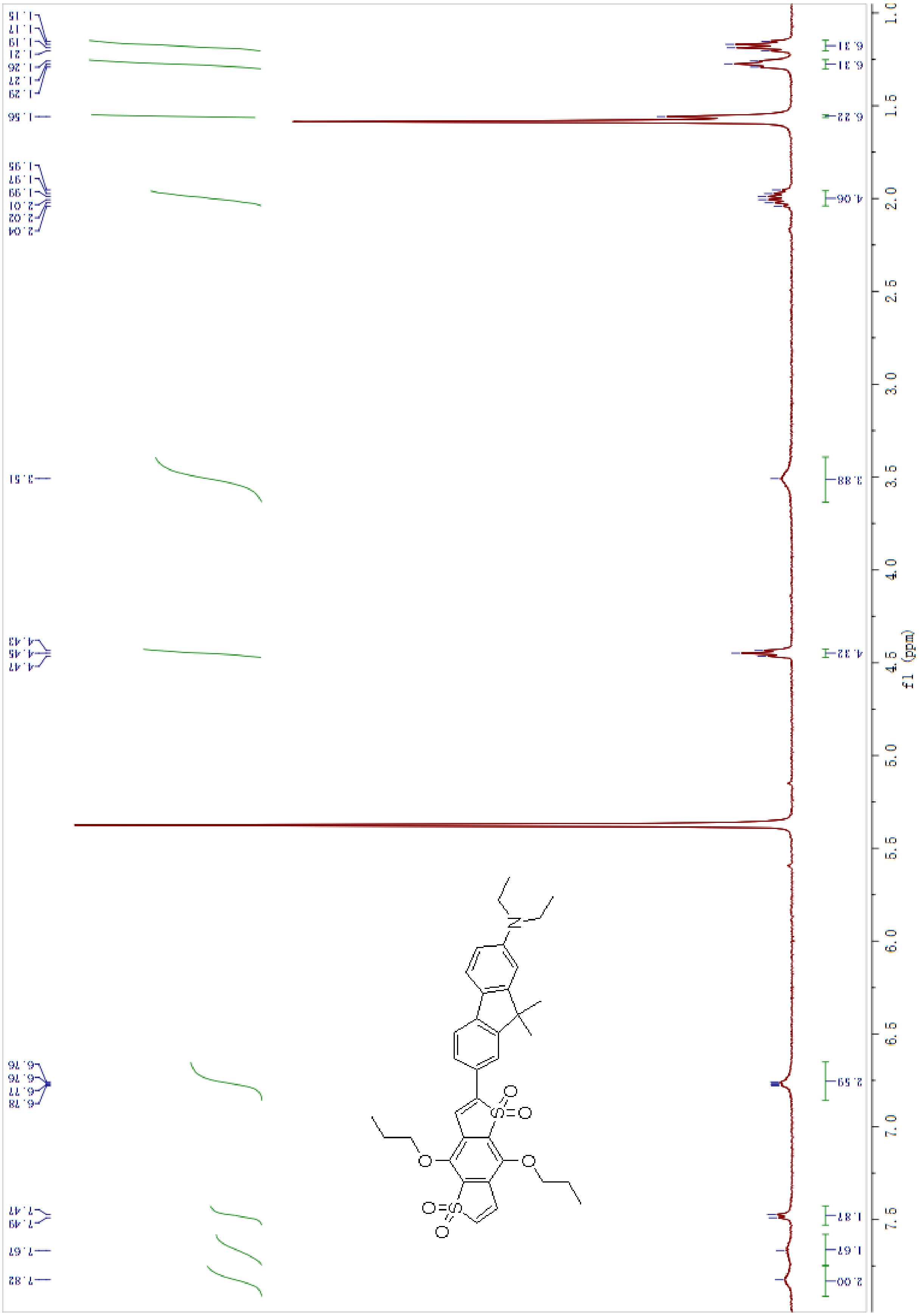

NMR-19. ^{1}H NMR spectrum of compound **12** (400 MHz, CD_2Cl_2).

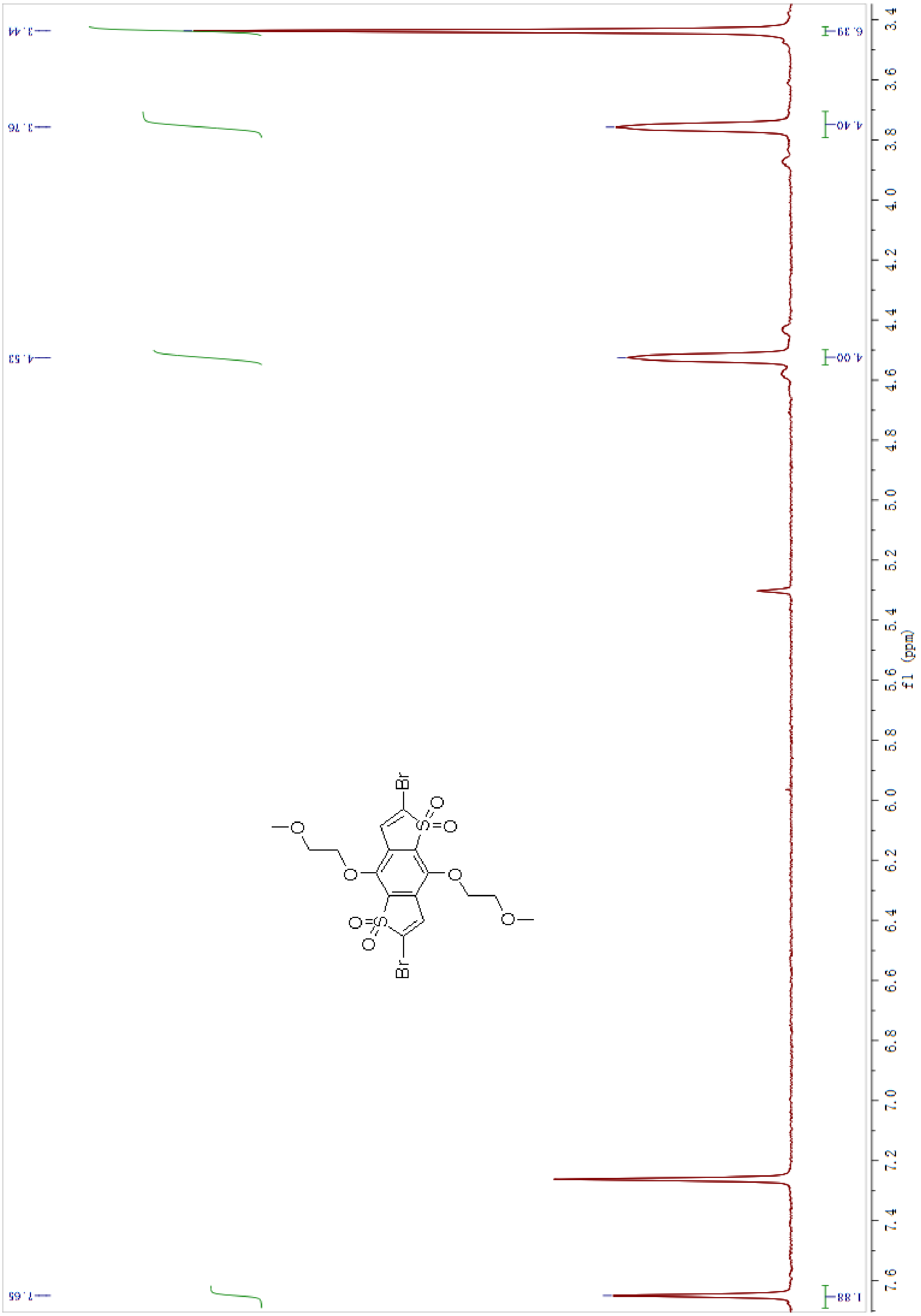

NMR-20. ^{1}H NMR spectrum of compound **C$_2$** (400 MHz, $CDCl_3$).

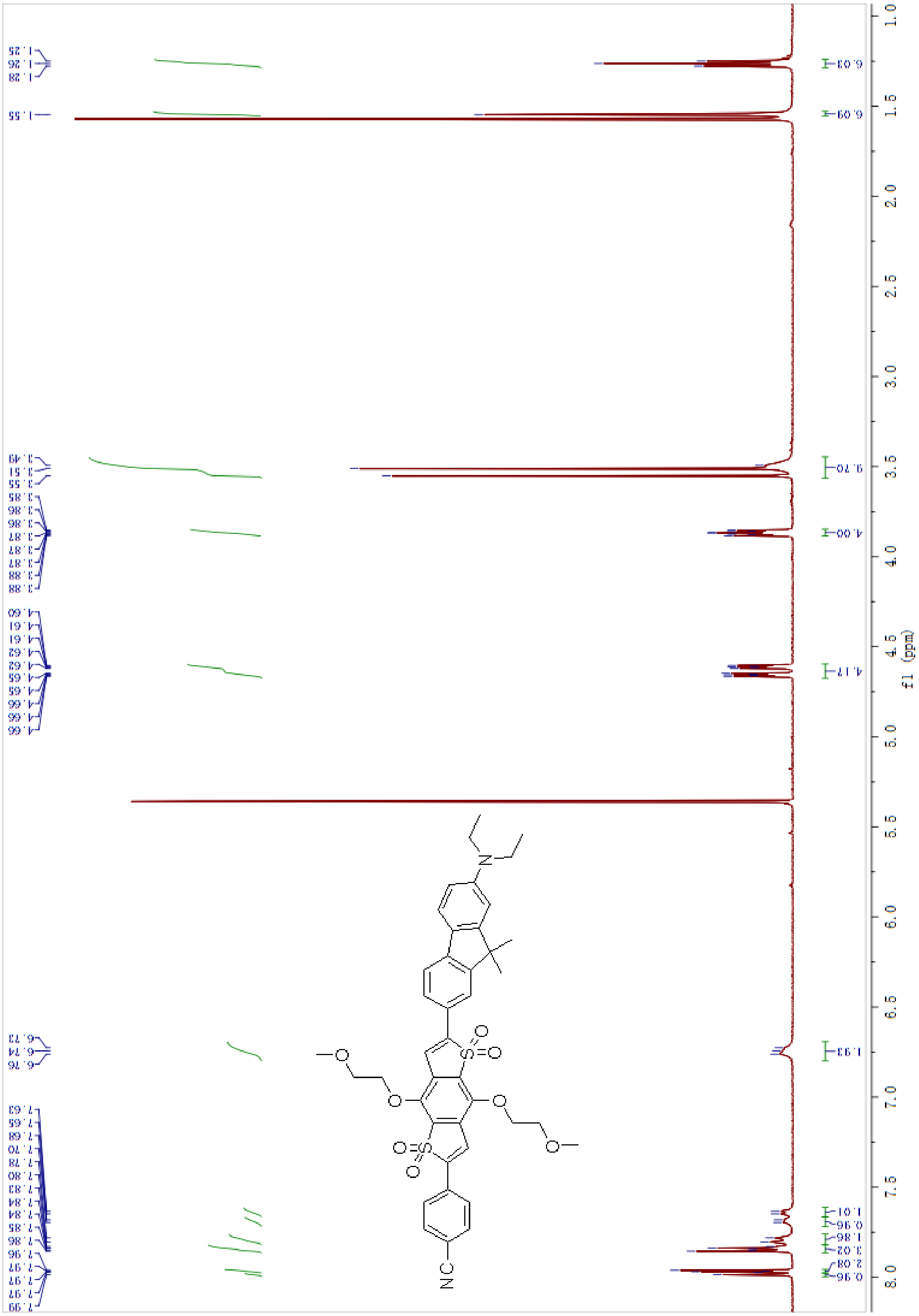

NMR-21. ^{1}H NMR spectrum of **Lipi-PS** (500 MHz, CD_2Cl_2).

Analysis Info

Analysis Name	D:\Data\MALDI\2023\1220\ZR-WU_0_L22_000001.d	Acquisition Date	12/20/2023 6:06:52 PM
Method	MALDI_P_100-3000	Operator	
Sample Name	MURU-N-ESI	Instrument	solariX
Comment			

Acquisition Parameter

Acquisition Mode	Single MS	Acquired Scans	4	Calibration Date	Fri Nov 10 05:21:59 2023
Polarity	Positive	No. of Cell Fills	1	Data Acquisition Size	2097152
Broadband Low Mass	202.1 m/z	No. of Laser Shots	10	Data Processing Size	4194304
Broadband High Mass	1600.0 m/z	Laser Power	16.0 lp	Apodization	Sine-Bell Multiplication
Source Accumulation	0.001 sec	Laser Shot Frequency	0.020 sec		
Ion Accumulation Time	0.010 sec				

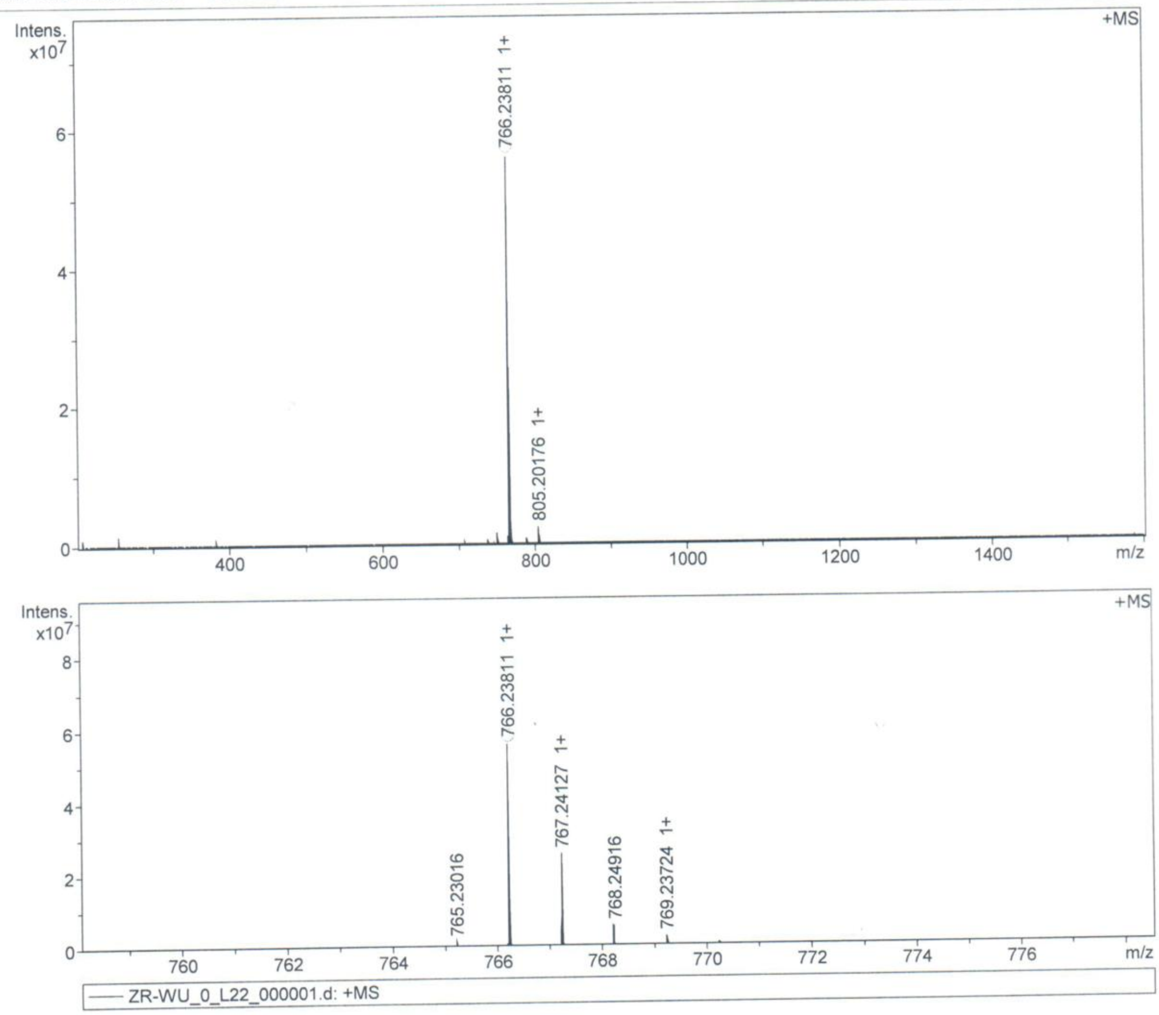

Meas. m/z	#	Ion Formula	Score	m/z	err [ppm]	Mean err [ppm]	mSigma	rdb	e⁻ Conf	N-Rule
766.238114	1	C42H42N2O8S2	100.00	766.237710	0.5	-0.5	88.6	23.0	odd	ok

MS-1. HR-MALDI-TOF mass spectra of **Lipi-PS**.
